# Dark times: iminothioindoxyl-*C*-nucleoside fluorescence quenchers with defined location and minimal perturbation in DNA†

**DOI:** 10.1039/d4sc05175k

**Published:** 2024-09-04

**Authors:** Larita Luma, Judith C. Pursteiner, Tobias Fischer, Rainer Hegger, Irene Burghardt, Josef Wachtveitl, Alexander Heckel

**Affiliations:** a Goethe University Frankfurt, Institute for Organic Chemistry and Chemical Biology Max-von-Laue-Str. 7 60438 Frankfurt Germany heckel@uni-frankfurt.de; b Goethe University Frankfurt, Institute for Physical and Theoretical Chemistry Max-von-Laue-Str. 7 60438 Frankfurt Germany wveitl@theochem.uni-frankfurt.de burghardt@chemie.uni-frankfurt.de

## Abstract

Fluorescence quenchers for application in DNA – like the BHQ family – tend to be large molecules which need to be attached, often post-synthetically, *via* long linkers. In this study, we present two new iminothioindoxyl-*C*-nucleosidic quenchers which are very compact, feature a native backbone and can be introduced into DNA *via* regular solid-phase synthesis. Especially with d*T* as juxtaposed nucleobase, they have a defined location and orientation in a DNA duplex with minimal perturbation of the structure and hence interaction capabilities. Depending on the nature of the fluorophore, they can be used for orientation-(un)specific FRET studies. Their Förster radius is smaller than the one of BHQ-2. This makes these quenchers ideal for sophisticated studies using conditional quenching in the range between 470 and 670 nm in DNA.

## Abstract 2

New small, rigid fluorescence quenchers, absorbing around 580 nm, show minimal perturbation in dsDNA structure.

## Graphical abstract

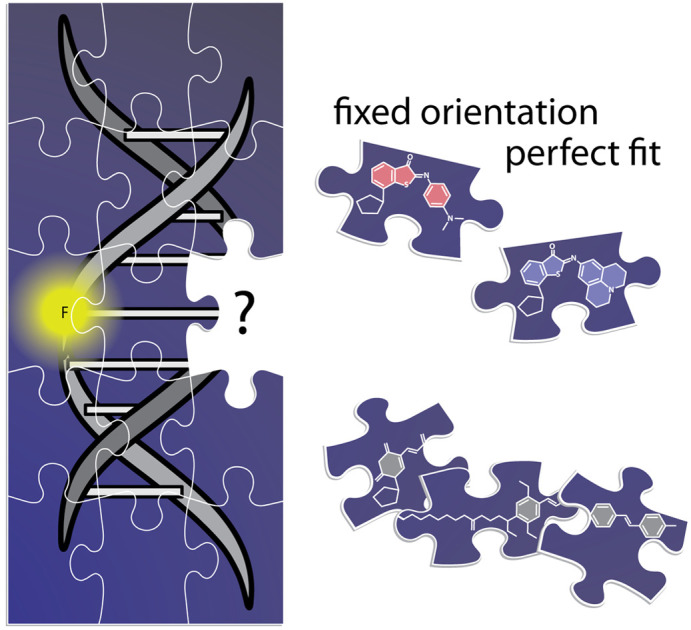

## Introduction

Fluorophores are an essential tool in cell biology and biochemistry.^1^ The use of fluorophores enables the investigation of a wide variety of biological processes in real time with detection limits down to single molecules.^2^ Tracking biomolecules such as oligonucleotides,^3–7^ proteins^8^ or lipids^9^ allows characterization of complex biological functions, the investigation of enzymatic activities and cellular signaling pathways. The versatility of fluorophore-labeled biomolecules extends across the fields of molecular diagnostics,^10^ drug development,^11^ molecular beacons,^12–14^ aptamers^15,16^ and microscopy applications.^17,18^

The application of fluorophores is even more useful, if their fluorescence is conditional – depending on local properties of the sample – by using quencher strategies, *e.g.* in a distance-dependent FRET effect.^8,15–18^ A very prominently-used quencher family is based on azobenzene-derived compounds like DABCYL.^19,20^ By expanding the chromophore, universal dark quencher systems like the BHQ (“black hole quencher”) series became accessible, covering a broad spectral range.^21,22^ As a consequence of this engineering of *λ*_max_ and *ε* for an optimal FRET efficiency, modern systems are rather large molecules. However, in many biologically active systems, small changes in the structure can cause a strong impact.^23,24^ Therefore, in the majority of cases, the established quenchers have to be attached *via* long, flexible linkers – outside of the main structure. This inevitable necessity positions the quencher away from otherwise desired, well-defined locations.

FRET-labels can be implemented *via* solid-phase synthesis or bioconjugation into oligonucleotides. By solid-phase synthesis, quenchers can be introduced terminally using 3′-modified solid support^25^ or 5′-modifiers.^26–28^ Within the sequence, specialized phosphoramidites are used, but only a handful of quencher molecules are directly accessible. In other cases, to avoid side reactions, phosphoramidites with an orthogonal coupling site are used and the quencher is installed post-synthetically in an additional coupling and purification step.^29^ Thus, for the majority of the commercially available quenchers there is no universal strategy for the introduction into oligonucleotides.

For fluorophore-quencher pairs there are many fascinating applications in an oligonucleotide context using several strategies.^30,31^ The development of chromophore-*C*-nucleosides by Kool^32–35^ and Leumann,^36–39^ is of particular importance in the context of this study. Leumann introduced for example hydrophobic biphenyl-*C*-nucleosides as FRET pairs into DNA oligonucleotides.^4,38^ Wilhelmsson developed unique nucleoside analogues as FRET pairs by π-system extension of the nucleobase.^40–42^ They characterized the structure and dynamics of DNA and RNA duplexes, utilizing the well-defined position and fixed orientation of their fluorophore-quencher pairs.^43–47^ Asanuma also introduced pyrene- and perylene-based FRET pairs in a fixed orientation due to π-stacking for characterization of DNA structure photo-dimerization.^48,49^ Sigurdsson showed that fluorescence, quenching and EPR spin label properties can be combined in nucleoside analogues that also have a fixed position in a duplex structure and can be used for investigations of RNA conformation and dynamics.^50^ However, the number of available quencher systems with rigid structures, a native backbone and good photophysical properties, including the absorption coefficient and the absorption maximum, remains very limited.

**Fig. 1:**
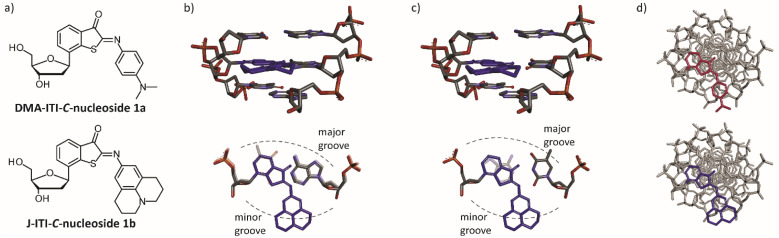
(a) The iminothioindoxyl-(ITI)-derived quencher nucleosides 1a and 1b introduced in this study. (b and c) Side and top views of our initial design idea (not geometry-optimized) in which one nucleoside of a dsDNA is replaced by nucleoside 1b opposite a d*A* or d*T* residue, respectively. For comparison, an unperturbed Watson–Crick–Franklin base pair is shown with faded colors. (d) Positioning of the ITI chromophores of compounds 1a and 1b in the native duplex structure viewed along the helix axis (not geometry-optimized).

In this work, two new quencher systems were established which feature a compact molecular structure in a rigid *C*-nucleosidic design that is compatible with a natural deoxyribose-phosphodiester backbone (Fig. 1a). Iminothioindoxyls (ITIs) are an emerging class of chromophores.^51,52^ In a previous study, we showed that their excited state decays surprisingly fast in a few picoseconds and used ITI-derivatives for time-resolved IR spectroscopy in a peptide context.^53^ Like in the previous study, we chose to use a dimethylamino- (DMA-ITI-*C*-nucleoside 1a) or julolidine- (J-ITI-*C*-nucleoside 1b) modification (“distal aromatic ring”) on the iminothioindoxyl core (“proximal aromatic ring”). Our decision to use *C*-nucleosides was motivated by previous experience, where we engineered photoswitches into oligonucleotide duplexes for reversible photocontrol of hybridization.^54–56^

Fig. 1b–d shows schematic illustrations of our initial design idea where a nucleoside in a dsDNA structure was replaced with compounds 1a and 1b, respectively. Panel 1b shows the (larger) J-ITI-*C*-nucleoside 1b opposite of a d*A* residue and panel 1c opposite of a d*T* residue, both from different, perpendicular, viewing angles. The corresponding pictures for the (smaller) DMA-ITI-*C*-nucleoside 1a are provided in the ESI (Section 5.3). From these illustrations, we expected a medium local influence on the duplex structure with a purine as juxtaposed nucleoside and an even smaller effect in the case of a pyrimidine. These preliminary considerations suggested that the proximal benzothiophenone part of the ITI could potentially be incorporated into the π-stacked base-pair ladder. It also suggested that even the larger of the two distal aromatic rings could still be accommodated in the minor groove.

## Results and discussion

**Scheme 1:**
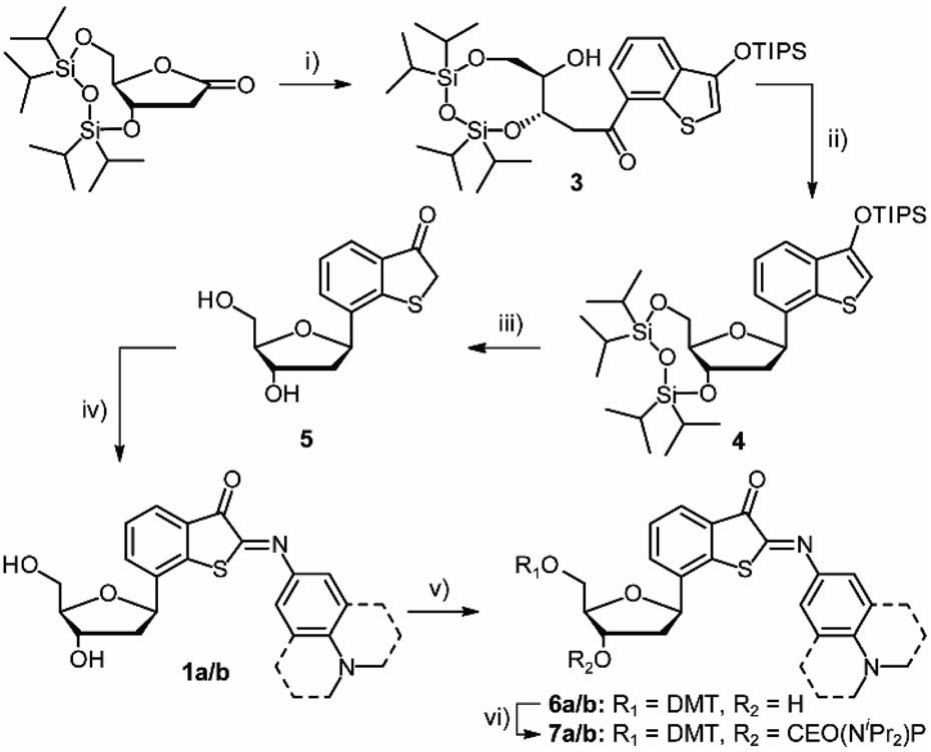
Synthesis of ITI phosphoramidites 7a/b. (i) THF, compound S2 (see Scheme S1, ESI†), *n*BuLi, −78 °C, 1.5 h, 55%. (ii) 1 DCM BF_3_·OEt_2_, Et_3_SiH, −78 °C, 4.5 h, 84%. (iii) THF, TBAF, rt, 2 h, 86%. (iv) EtOH, 1 M NaOH, DMA-/J-NO, rt, 16 h, 60%/58%. (v) DCM, pyridine, DMT-Cl, 0 °C – rt, 24 h, 34%/44%. (vi) DCM, DIPEA, PN(^i^Pr)_2_OCE-Cl, rt, 2.5 h, 44%/92%.

Starting from TIPDS-protected ribonolactone (2),^57,58^ the benzothiophenone (BTP) moiety was introduced by a nucleophilic attack to the 1′-position of a suitable precursor after lithium-halogen-exchange (→3, Scheme 1). Recovery of the ribose scaffold was performed by Lewis acid-catalyzed dehydroxylation (→4).^59^ The desired β-configuration was restored with an anomeric ratio of >90%, shown in ROESY-NMR spectroscopy (ESI, Section 2). The free nucleoside 5 was obtained after TBAF deprotection of the silyl-protecting groups. The distal aromatic ring was introduced utilizing an intermolecular aldol-like condensation with previously prepared nitroso compounds to yield DMA-/J-ITI-nucleosides 1a/b. For incorporation of nucleoside 1a/b into oligonucleotides *via* solid-phase synthesis, the 5′-OH group was tritylated (6a/b) and the 3′-OH group was phosphitylated (7a/b). A detailed description of the synthesis is provided in the ESI (Section 1).

**Fig. 2:**
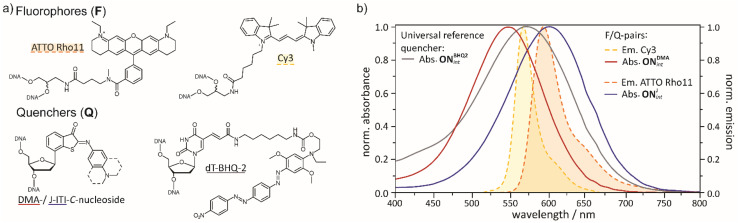
(a) Chemical structures of quencher and fluorophore moieties incorporated for oligonucleotide modification. (b) Overview of the emission spectra of the fluorophores Cy3 and ATTO Rho11 as small molecules (dashed lines and filled areas)^60^ as well as the absorbance spectra of the DMA-ITI-, J-ITI- and BHQ-2-modified oligonucleotides of this study (solid lines).

Apart from the structural properties of quencher molecules, their photochemical properties are of great importance. The molar absorbance spectra of compounds 1a and 1b in MeOH and MeOH/PBS (pH 7.4) are shown in Fig. 2 and Table 1. Despite their small molecular structure, they have relatively high absorption bands in the visible range between 450 and 650 nm with maximal molar absorbance coefficients of 25 900 to 33 200 M^−1^ cm^−1^ (see also ESI, Section 3) – albeit not as high as the ones of the typical azobenzene-based quenchers. In comparison, the absorption coefficients of DABCYL, BHQ-1, BHQ-2 and BHQ-3 are 32 000 M^−1^ cm^−1^, 34 000 M^−1^ cm^−1^, 38 000 M^−1^ cm^−1^ and 42 700 M^−1^ cm^−1^, respectively.^2^ The full widths at half maximum (FWHM) of the adsorption bands are in the range of 97–119 nm, while for example the one of BHQ-2 is 139 nm.^60^ It depends on the type of application, whether wider or more narrow FWHM values are preferred. The latter are for example advantageous in multi-color applications.^61,62^

**Table 1:** Absorption properties of DMA-ITI-1a and J-ITI-*C*-nucleoside 1b

Compound	Solvent	*λ*_max_/nm	*ε*/M^−1^ cm^−1^	FWHM/nm
1a	MeOH	519	27 000	97
1a	MeOH/PBS 1 : 1	539	25 900	111
**ON** ^**DMA**^_**int**_	MeOH/buffer^a^	548	24 400	112
1b	MeOH	563	33 200	105
1b	MeOH/PBS 1 : 1	591	31 000	119
**ON** ^**J**^_**int**_	MeOH/buffer^a^	602	31 300	124

a Water, hexafluoroisopropanol (0.4 M), Et_3_N (16.3 mM), pH 7.9.

In additional ultrafast UV-Vis transient absorption measurements, the relaxation behavior and dynamics of the ITI-derivatives were characterized (ESI, Section 4). Both derivatives are permanently present in their *Z*-conformation, showing no formation of long-lived photoproducts due to photo-isomerization or other productive pathways after excitation. Relaxation takes place within a few picoseconds exclusively *via* vibrational deexcitation.

To test the quenching properties of our new compounds 1a/b, we chose the commercially available Cy3 and ATTO Rho11 as fluorophores (Fig. 2a). Based on the respective emission and absorbance properties (Fig. 2b), we combined Cy3 with DMA-ITI as well as ATTO Rho11 with J-ITI. As reference quencher d*T*-BHQ-2 was selected, which can be used as quencher for either fluorophore.

**Fig. 3:**
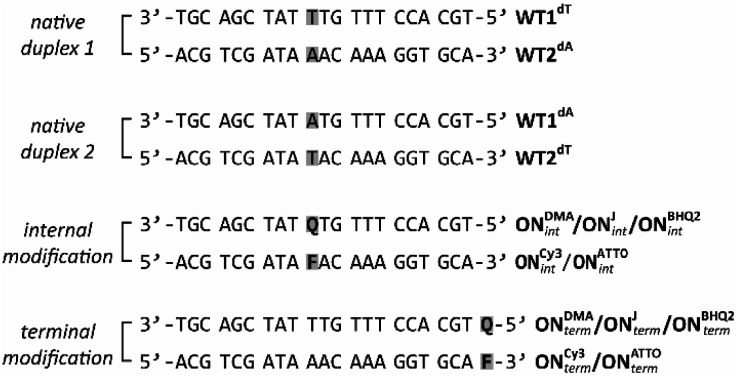
(Modified) oligonucleotides used in this study. The internal or terminal position of the quencher modification is designated with Q, the one of the fluorophore with F.

These modifications were investigated in an oligonucleotide context. To that end, phosphoramidites 7a/b were incorporated using standard solid-phase synthesis procedures (Fig. 3 and ESI Section 5). Potential hydrolysis of the imine in the ITI was only observed in the presence of for example 80% acetic acid, which is why we recommend a DMT-OFF strategy for the solid-phase synthesis. The sequence was selected to have no stable secondary structures and a suitable melting temperature.^63^ Also, we avoided guanine in the immediate vicinity of the fluorophore as the guanosine residue itself has a fluorescence quenching effect based on the photo-induced electron transfer (PET)^64,65^ between the fluorescence dye and the nucleobase.^66–70^ The quenching properties of guanosine have been used for the development of “quencher-free” molecular beacons for example by Sauer and Kim.^64,71,72^ For the internal modification, a residue of the 21-mer was replaced, while the terminal modifications were added to the respective 5′- and 3′-ends so that the same antisense strands could be used for all sequences. The fluorophore-modified antisense strands were synthesized by NHS-labelling of the corresponding amino-functionalized oligonucleotides (ESI, Section 6).

Compared to the free nucleosides 1a/b in MeOH/PBS, in the oligonucleotides, *λ*_max_ of the ITI chromophores' absorbance is slightly red-shifted by approximately 10 nm, the absorption coefficients are slightly reduced but only in the case of **ON**^**DMA**^_**int**_ and the FWHM remains approximately the same. BHQ-2 on the other hand experiences a slight increase in the absorbance coefficient (38 000–40 700 M^−1^ cm^−1^) upon introduction into **ON**^**BHQ2**^_**int**_, while *λ*_max_ (579 nm–574 nm) and the FWHM remain approximately the same (139 nm–144 nm).

**Fig. 4:**
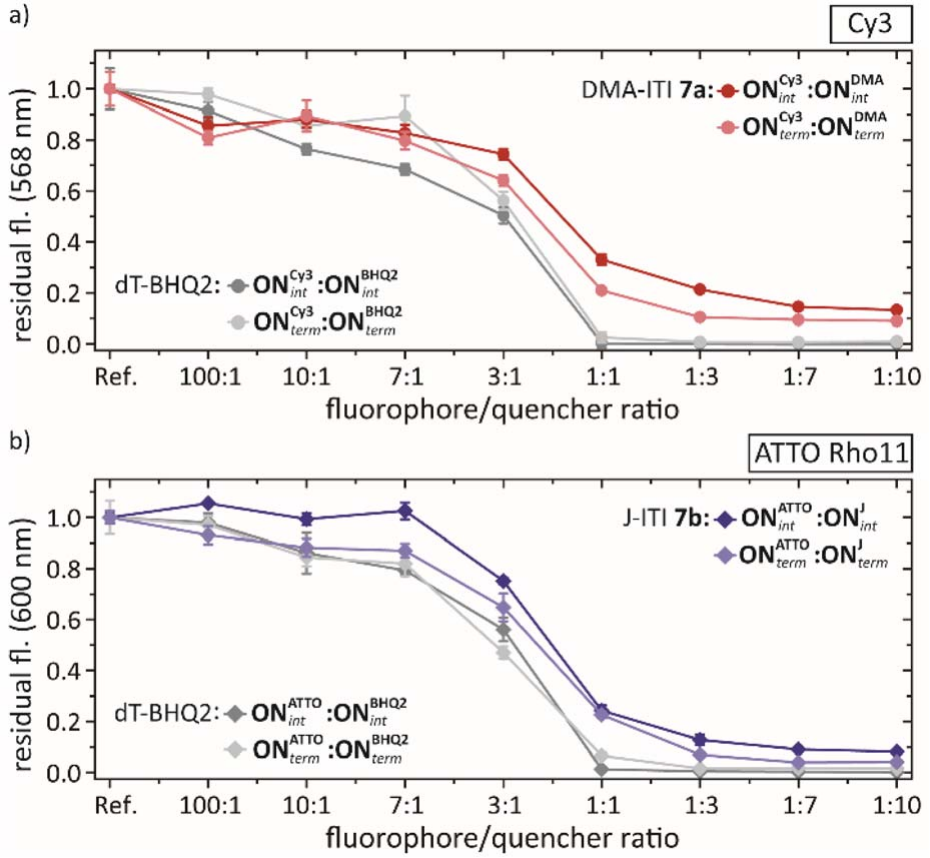
Fluorescence studies of the F/Q-pair-modified oligonucleotide duplexes. The respective F-labelled strand serves as the reference for maximum fluorescence. (a) Fluorescence quenching of Cy3 with DMA-ITI-*C*-nucleoside 7a internal (dark red), terminal (light red). (b) Fluorescence quenching of ATTO Rho11 with J-ITI-*C*-nucleoside 7b internal (dark blue), terminal (light blue). For both fluorophores d*T*-BHQ-2 internal (dark grey), terminal (light grey) was used for comparison.

To evaluate the quenching effects, fluorescence studies were performed with different fluorophore/quencher strand ratios (100 : 1, 10 : 1, 7 : 1, 3 : 1, 1 : 1, 1 : 3, 1 : 7, 1 : 10), keeping the fluorophore concentration constant (Fig. 4 and ESI Section 7). For the fluorophore/quencher pair Cy3 and DMA-ITI, the fluorescence was quenched to 33% (1 : 1) and 13% (1 : 10) for the internal modification, respectively. For the terminal modification, we observed an even lower fluorescence: 21% (1 : 1) and 9% (1 : 10).

With the J-ITI quencher, featuring the larger distal aromatic ring, and with ATTO Rho11 we found 24% (1 : 1) and 8% (1 : 10) for the internal, as well as 12% (1 : 1) and 5% (1 : 10) for the terminal modification. In the direct comparison of these numbers, one has to keep in mind that in the fluorophore/BHQ-2 pairs will most likely perform contact quenching, as both chromophores are attached *via* long linkers in the major groove.^3,61,73^ In the fluorophore-ITI pairs, this is impossible and the quenching is most likely FRET-based. The calculated Förster radius *R*_0_ of **ON**^**Cy3**^_**int**_:**ON**^**DMA**^_**int**_ is 4.7 nm, while the one of **ON**^**Cy3**^_**int**_:**ON**^**BHQ2**^_**int**_ is 5.3 nm. Likewise, *R*_0_ for **ON**^**ATTO**^_**int**_:**ON**^**J**^_**int**_ is 6.1 nm, while the one for **ON**^**ATTO**^_**int**_:**ON**^**BHQ2**^_**int**_ is 6.3 nm. Thus, *R*_0_ for our new quenchers is smaller compared to BHQ-2. This can be advantageous in specific applications.^74,75^

For quantitative FRET measurements, it is also important to consider the influence of the orientation of the two chromophores (*κ*) as evidenced in the work by Sauer and Seidel as well as Wilhelmsson and Asanuma.^47–49,76^ For two freely-rotating chromophores, *κ*^2^ is often approximated as 2/3 (*i.e.* isotropic average). With our quenchers, which have a fixed position in the base pair stack, both scenarios are possible: with a freely rotating donor (as in our examples), the same approximation is still valid, whereas with a fixed donor, experiments with a more complicated *κ*-dependence are possible. We then tested the influence of our quenching residues on the structure by melting temperature studies and CD spectroscopy (Table 2 and ESI, Sections 8 and 9). All measurements were carried out under identical conditions comparing the set of BHQ-2, DMA-ITI and J-ITI. The first measurements were performed with a purine d*A* as the juxtaposed nucleobase (**WT2**^**dA**^ as antisense strand) – which was expected to be still too large for a minimal perturbation. The melting temperature of the native duplex was determined to be 70.3 °C. As expected, terminal modification has little or no effect due to the increased degrees of freedom, although it should be emphasized that BHQ-2 shows the greatest perturbation. With internal modification, a larger Δ*T*_m_ was observed for all three constructs. DMA-ITI shows the largest interference with 7 °C despite its small structure. BHQ-2 and J-ITI induce a significantly lower perturbation with Δ*T*_m_ of 4–4.5 °C. CD spectroscopy showed that the structure of a native B-helix is preserved and that only minor deviations are induced by all the constructs described above, consistent with the results of the melting temperature study.

**Table 2:** Overview of the results of melting temperature studies between the indicated, modified oligonucleotide strands

Antisense strand: **WT2**^**dA**^			Antisense strand: **WT2**^**dT**^		
Sense strand	*T*_m_/ °C	Δ*T*_m_/ °C	Sense strand	*T*_m_/ °C	Δ*T*_m_/ °C
**WT1** ^**dT**^	70.3	—	**WT1** ^**dA**^	67.7	—
**ON** ^**DMA**^_**term**_	70.5	+0.2			
**ON** ^**J**^_**term**_	71.1	+0.8			
**ON** ^**BHQ2**^_**term**_	68.7	−1.6			
**ON** ^**DMA**^_**int**_	63.3	−7.0	**ON** ^**DMA**^_**int**_	65.7	−2.0
**ON** ^**J**^_**int**_	65.8	−4.5	**ON** ^**J**^_**int**_	66.4	−1.3
**ON** ^**BHQ2**^_**int**_	66.2	−4.1	**ON** ^**BHQ2**^_**int**_	66.5	−1.2

The next series of tests was performed with a pyrimidine d*T* as the opposite nucleobase (**WT2**^**dT**^ as antisense strand) from which we expected a much better fit in our initial considerations. Indeed, we observed that the entire trio induces a significantly smaller perturbation of only 1.2–2.0 °C.

Interestingly, the temperature-dependent hyperchromicity of the nucleobase absorption band at 260 nm coincides with the temperature-dependent hypsochromic shift of the quenchers' absorbances (ESI Section 3.2) – further supporting the hypothesis of base stacking of the new quenchers.

**Fig. 5:**
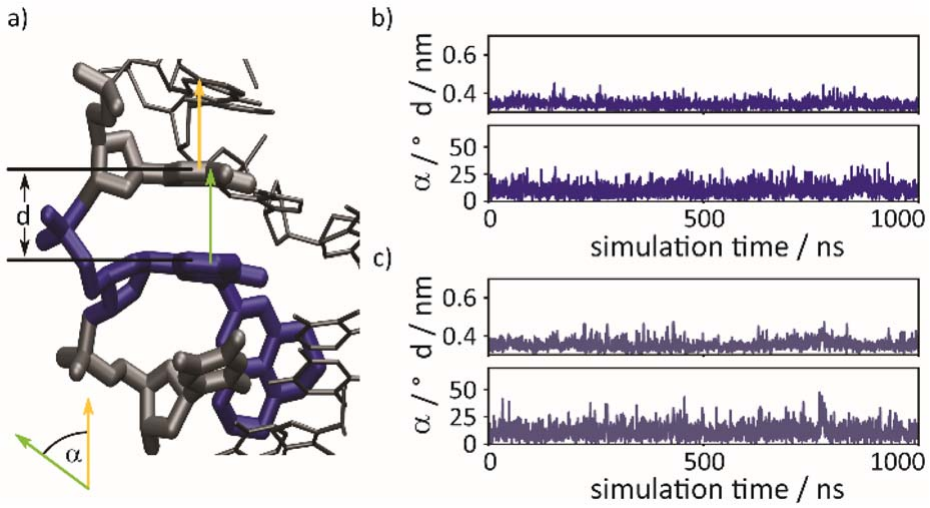
(a) Representative snapshot taken from an all atom MD simulation at *T* = 300 K, with the J-ITI-*C*-nucleoside 1b highlighted in blue. The stacking distance parameter *d* and the tilt angle *α* between normal vectors of the proximal aromatic ring of the quencher and upper nucleobase (marked in green and orange, respectively) measure stacking fluctuations. (b) Time traces for the parameters *d* and *α* for the **WT2**^**dA**^ modified duplex (see Table 2). (c) Corresponding time traces of the parameters *d* and *α* for the **WT2**^**dT**^ duplex.

To probe the local perturbation further, we performed molecular dynamics (MD) simulations. All-atom MD simulations confirm that the proximal aromatic ring of the quencher exhibits π-stacking that is very similar to the one of native interactions. This is illustrated in Fig. 5 – taken from a typical simulation run – which shows the quencher and its local environment (Fig. 5a). The julolidine ring is twisted to follow the path of the minor groove. For the **WT2**^**dA**^-modified duplex, Fig. 5b shows the distance parameter *d* between the proximal aromatic ring of the J-ITI-*C*-nucleoside 1b and the upper neighboring nucleobase as a function of time, along with the tilt angle *α* between the normal vectors pertaining to these moieties. A similar analysis is shown in Fig. 5c for the **WT2**^**dT**^ duplex. The deviations of both the *d* and *α* quantities from their average values are similar to the ones found in native π-stacking, as illustrated in Section 10 of the ESI. However, the π-stacking interaction with the lower nucleobase is less tight and prone to stronger fluctuations, as also shown in Section 10 of the ESI. Our simulations further suggest that the distal aromatic ring of the J-ITI-*C*-nucleoside 1b is rotated with respect to the plane of the proximal aromatic ring, following the path of the minor groove, and is perfectly accommodated in the latter. These results all support our initial design ideas (Fig. 1b and c) regarding the perturbation potential of our modifications with a purine or pyrimidine as the adjacent nucleobase. Previous quencher systems require long spacers to position the sterically demanding chromophore outside of the base stack. In contrast, our DMA- and J-ITI-*C*-nucleosides use a deoxyribose with β-configuration at the anomeric center as the smallest, most native point of attachment to the oligonucleotide backbone.

## Conclusion

We present two new *C*-nucleosidic quenchers based on an iminothioindoxyl (ITI) chromophore system. They have a relatively small, rigid structure compared to other quenchers like the BHQ family and were designed to fit with minimal perturbation of the native structure as nucleotide replacement in a DNA duplex opposite a pyrimidine nucleoside. Thermal melting studies and MD simulations support the idea that the quenchers are part of the base-pair stack in a fixed position. This is in contrast to for example the BHQ family of quenchers, which are too large for a DNA duplex and are usually attached *via* a flexible linker in the major groove, where proteins are known to interact with a DNA duplex. Since our quenchers have a native deoxyribose backbone, they can be introduced *via* solid-phase synthesis in any position of the oligonucleotide.

The molar absorption coefficients of our quenchers are smaller than the ones of the BHQ family but still in the same order of magnitude – despite their small size. Importantly, their Förster radius is smaller than the ones of the BHQ derivatives. Also, their absorption is surprisingly far in the green/red part of the spectrum. Choosing either a conformationally restricted or a flexible FRET donor, different types of orientation-(un)specific spectroscopic investigations are possible.

While quenchers and fluorophores attached to dsDNA *via* long linkers offer the potential for contact quenching and hence a minimal fluorescent background, we believe that the strength of our quenchers lies in different directions of application: superresolution microscopy could profit from the small Förster radius.^74,75^ Spectroscopic studies on the dynamic folding of aptamers and riboswitches will profit from the small size, minimal perturbation and well-defined position and do not require full quenching.^77^ Also, functional biological studies may profit from the small size and fixed location of our quenchers.^78^

## Author contributions

L. L.: conceptualization, data curation, investigation, resources, formal analysis, visualization, writing – original draft, writing – review & editing. J. P.: data curation, investigation, resources, formal analysis. T. F.: data curation, investigation, formal analysis, visualization. R. H.: simulations, investigation, formal analysis, visualization. I. B.: funding acquisition, project administration, writing – review & editing. J. W.: funding acquisition, project administration, writing – review & editing. A. H.: conceptualization, funding acquisition, project administration, writing – original draft, writing – review & editing.

## Conflicts of interest

There are no conflicts to declare.

## Supplementary Material

SC-015-D4SC05175K-s001

## Data Availability

The data supporting this article have been included as part of the ESI.†
