# Supplementary material for: Dark times: iminothioindoxyl-C-nucleoside fluorescence quenchers with defined location and minimal perturbation in DNA
Source: Chem Sci. 2024 Sep 4;15(38):15783–9. doi: 10.1039/d4sc05175k (PMC11388086; doi:10.1039/d4sc05175k)
Supplement: SC-015-D4SC05175K-s001 [file SC-015-D4SC05175K-s001.pdf]

Supplementary Information (SI) for Chemical Science.
This journal is © The Royal Society of Chemistry 2024

Electronic Supplementary Information (ESI)

Dark Times: Iminothioindoxyl-*C*-Nucleoside Fluorescence Quenchers with Defined Location and Minimal Perturbation in DNA

Larita Luma,[a] Judith C. Pursteiner,[a] Tobias Fischer,[b] Rainer Hegger,[b] Irene Burghardt,*[b] Josef Wachtveitl,*[b] and Alexander Heckel *[a]

Abstract: Fluorescence quenchers for application in DNA – like the BHQ family - tend to be large molecules which need to be attached, often postsynthetically, via long linkers. In this study, we present two new iminothioindoxyl-*C*-nucleosidic quenchers which are very compact, feature a native backbone and can be introduced into DNA via regular solid-phase synthesis. Especially with dT as juxtaposed nucleobase, they have a defined location and orientation in a DNA duplex with minimal perturbation of the structure and hence interaction capabilities. Depending on the nature of the fluorophore, they can be used for orientation-(un)specific FRET studies. Their Förster radius is smaller than the one of BHQ-2. This makes these quenchers ideal for sophisticated studies using conditional quenching in the range between 470 and 670 nm in DNA.

DOI:

Table of Contents

1. Chemical Synthesis 1
   1.1. Material and Methods 1
   1.2. TIPS-protected Chromophore Core Structure S2 1
   1.3. DMA- / and J-ITI-Phosphoramidites 7a/b 2
2. Anomeric Configuration of the Modified Nucleoside 6
3. Photochemical Experiments (UV/Vis) 7
   3.1. Absorption Spectra / Extinction Coefficients 7
   3.2. Hypochromicity of DMA- and J-ITI Chromophores in double stranded DNA 8
   3.3. Determination of the Förster Distance R_0 9
4. Ultrafast Vis Measurements 10
   4.1. Materials and Methods 10
   4.2. Results and Discussion 11
5. Oligonucleotide Synthesis 12
   5.1. Materials and Methods 12
   5.2. Purification 13
   5.3. Hypothesis – Structural Orientation of the ITI-Chromophore within the DNA Duplex 14
6. Fluorophore NHS-Labeling 14
7. Fluorescence Studies 15
   7.1. Materials and Methods 15
   7.2. Results 15
   7.3. Primary Data 16
8. Melting Temperature Studies 17
   8.1. Materials and Methods 17
   8.2. Results 17
   8.3. Primary Data 19
9. CD Spectroscopy 21
   9.1. Materials and Methods 21
   9.2. Results 21
10. MD Simulations 22
11. NMR Spectroscopy 23
12. Mass Spectra 36
13. Oligonucleotide Mass Spectra 41
14. List of Abbreviations 47
15. References 48

1. Chemical Synthesis

1.1. Material and Methods

Unless stated otherwise, all reactions were performed under a protective argon atmosphere using dry solvents. All reagents and solvents were purchased from commercial sources and used without further purification. Dry solvents were purchased over molecular sieves from *Acros Organics*. Reactions were monitored using silica gel 60-coated TLC sheets with fluorescence indicator UV_{254} (*ALUGRAM Xtra SIL G/UV*) from *Macherey-Nagel*. Crude products were purified by manual column chromatography using silica gel 60 (0.04 – 0.063 mm) from *Macherey-Nagel*.

If not stated otherwise, NMR spectra were recorded in chloroform ($CDCl_3$-*d*), and dimethyl sulfoxide (DMSO-d_6) from *euroiso-top* using a *Bruker Avance AV 400 MHz*, *Bruker 400'54 Ascend* or a *Bruker Avance III HD AV500 MHz* spectrometer at room temperature. All shifts are reported in ppm using the solvent signal as an internal reference (^{1}H: 7.26 ppm $CDCl_3$-*d*, ^{1}H: 2.50 ppm DMSO-d_6; ^{13}C: 77.16 ppm $CDCl_3$-*d*, ^{13}C: 39.52 ppm DMSO-d_6). $CDCl_3$-*d* was filtered through basic Al_2O_3 before dipeptide spectra were recorded. The following abbreviations were used to describe multiplicities: s = singlet, d = doublet, t = triplet, q = quartet, m = multiplet, (b) = broad signal. Coupling constants are reported in Hertz (Hz).

Electrospray ionization (ESI) mass spectra were obtained on a *Thermo Fisher Surveyor MSQ device* and high-resolution mass spectrometry (HRMS) was conducted on a *LTQ Orbitrap XL* by *Thermo Fisher* (MALDI-HRMS), a *Bruker* MicroTof-qII (ESI-HRMS) or a *Waters* Synapt G2s (ESI-HRMS).

1.2. TIPS-protected Chromophore Core Structure S2

Starting from 2-bromothiophenone, the synthesis of the chromophore core structure bromobenzothiophenone **BTP** was carried out over two steps following Yamaguchi *et al.*[1] with some adjustments, as previously described.[2] The silyl-protection of the keto group of **BTP** was carried out using NaH and triisopropylsilylchloride (TIPSCl) to yield compound **S2**. The reaction scheme is shown in Scheme S1. Nitroso compounds used in the course of this project were synthesizes according to the mild procedure in our previous work.[2]

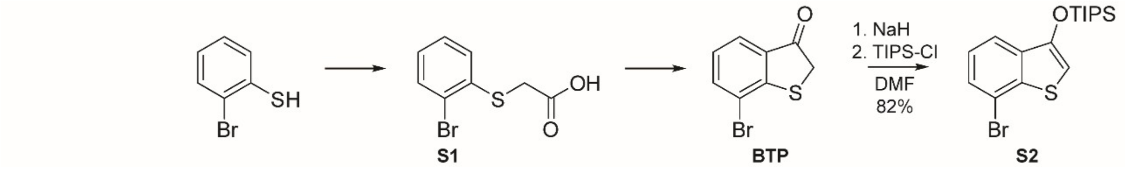

Scheme S1 Synthesis of the TIPS-protected chromophore core structure **S2.**

((7-Bromobenzo[b]thiophen-3-yl)oxy)triisopropylsilane (S2)

Compound **3** (16.68 g, 72.80 mmol, 1.0 eq) was dissolved in 300 mL dry DMF. The addition of NaH (60% in mineral oil) (3.20 g, 80.1 mmol, 1.1 eq) was carried out at room temperature, and the reaction mixture was stirred for 30 minutes. Subsequently, TIPSCl (17.0 mL, 80.1 mmol, 1.1 eq) was added at room temperature, and the reaction mixture was stirred for additional 30 minutes. The reaction was quenched by addition of 20 mL of MeOH, followed by removal of the solvent under reduced pressure. The residue was extracted with DCM, the combined organic layers were dried over Na_2SO_4, filtered and the solvent was removed under reduced pressure. The crude product was purified by column chromatography (Cy:EA 99:1 – 49:1). Compound **S2** was obtained as a yellow-green oil (23.06 g, 59.83 mmol, 82%).

TLC: R_f = 0.75 (cyclohexane/ethyl acetate 3:1)

^{1}H-NMR: (500 MHz, $CDCl_3$-*d*) δ (ppm) = 7.76 (dd, *J* = 7.9, 0.7 Hz, 1H, Ar *H*-6), 7.51 (d, *J* = 7.5 Hz, 1H, Ar *H*-4), 7.25 (t, *J* = 7.8 Hz, 1H, Ar *H*-5), 6.49 (s, 1H, Ar C*H*), 1.40 – 1.30 (m, 3H, TIPS-C*H*), 1.14 (d, *J* = 7.5 Hz, 18H, TIPS-CH_3).

^{13}C{^{1}H}-NMR: (126 MHz, $CDCl_3$-*d*) δ (ppm) = 147.49, 138.78, 135.81, 127.79, 125.23, 120.39, 116.79, 104.01, 18.08, 12.75.

^{29}Si-INEPT-NMR: (99 MHz, $CDCl_3$-*d*) δ (ppm) = 18.42.

ESI-HRMS: *m/z* calculated for $C_{17}H_{25}BrOSSi$ $[M+H]^+$ 385.0652; found 385.0654 (Δ_m = 0.0002, Δ_m/m = 0.6 ppm).

1.3. DMA- / and J-ITI-Phosphoramidites 7a/b

2-((6*S*,7*R*)-7-hydroxy-2,2,4,4-tetraisopropyl-1,3,5,2,4-trioxadisilocan-6-yl)-1-(3-((triisopropylsilyl)oxy)benzo[b]thiophen-7-yl) ethan-1-one (3)

In a first flask, compound **2** (28.02 g, 74.79 mmol, 1.0 eq) was dissolved in 100 mL dry THF. In a second flask, compound **S2** (28.73 g, 82.27 mmol, 1.1 eq) was dissolved in 150 mL dry THF. Both flasks were cooled to -80 °C, and 2.5 M *n*BuLi (32.91 mL, 82.27 mmol, 1.1 eq) was added to the second flask. The reaction mixture was stirred for 45 minutes at -80 °C. Subsequently, the contents of flask 1 were added to flask 2. The reaction mixture was stirred for additional 3 hours at -80 °C. The reaction mixture was quenched by fast addition to saturated NH_4Cl solution. The extraction was carried out with DCM, the combined organic layers were dried over Na_2SO_4 and the solvent was removed under reduced pressure. The residue was purified by column chromatography (Cy:EA 19:1 – 9:1 – 4:1). Compound **3** was obtained as a yellow-green oil (27.80 g, 40.81 mmol, 55%).

TLC: R_f =0.24 (cyclohexane/ethyl acetate 9:1)

^{1}H-NMR: (500 MHz, DMSO-*d6*) δ (ppm) = 8.29 (d, *J* = 7.4 Hz, 1H, Ar *H*-6), 7.98 (d, *J* = 7.8 Hz, 1H, Ar *H*-4), 7.59 (t, *J* = 7.7 Hz, 1H, Ar *H*-5), 6.87 (s, 1H, Ar C*H*), 5.08 (d, *J* = 5.8 Hz, 1H, 4'-O*H*), 4.46 (td, *J* = 8.8, 2.6 Hz, 1H, 3'-*H*), 4.03 (d, *J* = 10.7 Hz, 1H, 5'-C*H2*), 3.77 (dd, *J* = 11.8, 1.5 Hz, 1H, 5'-C*H2*), 3.55 (dd, *J* = 15.0, 2.7 Hz, 1H, 2'-C*H2*), 3.40 – 3.35 (m, 1H, 4'-*H*), 3.26 (dd, *J* = 15.1, 8.2 Hz, 1H, 2'-C*H2*), 1.34 (dt, *J* = 14.8, 7.4 Hz, 3H, TIPS-C*H*), 1.10 – 1.07 (m, 18H, TIPS-C*H3*), 1.03 – 0.97 (m, 16H, TIPDS-C*H3*), 0.93 – 0.88 (m, 8H, TIPDS-C*H3*), 0.79 (d, *J* = 7.4 Hz, 2H, TIPDS-C*H*), 0.54 (d, *J* = 7.3 Hz, 2H, TIPDS-C*H3*).

^{13}C{^{1}H}-NMR: (126 MHz, DMSO-*d6*) δ (ppm) = 198.73, 145.39, 135.29, 134.95, 130.19, 128.72, 125.48, 124.09, 107.39, 74.07, 68.64, 62.59, 54.90, 43.65, 26.33, 17.74, 17.29, 17.22, 17.15, 17.14, 17.11, 17.08, 17.00, 16.75, 12.75, 12.66, 12.13, 12.06, 11.93.

^{29}Si{^{1}H}-NMR: (99 MHz, DMSO-*d6*) δ (ppm) = 18.04, -12.87, -15.29.

MALDI-HRMS: *m/z* calculated for $C_{34}H_{60}O_6SSi_3$ [M+Na]$^+$ 703.33106; found 703.32980 (Δ_m = 0.00126, Δ_m/m = 1.8 ppm).

(6a*R*,9a*S*)-2,2,4,4-tetraisopropyl-8-(3-((triisopropylsilyl)oxy)benzo[b]thiophen-7-yl)tetrahydro-6H-furo[3,2-f][1,3,5,2,4] trioxadisilocine (4)

Compound **3** (16.41 g, 24.09 mmol, 1.0 eq) was dissolved in 200 mL dry DCM and cooled to -80 °C. Subsequently, $BF_3{\cdot}OEt_2$ (9.2 mL, 72 mmol, 3.0 eq) was rapidly added, followed by the slow addition of Et_3SiH (19.4 mL, 121 mmol, 5.0 eq) using a dropping funnel over a period of 30 minutes. After 2 hours at -80 °C, $BF_3{\cdot}OEt_2$ (9.2 mL, 72 mmol, 3.0 eq) was added, followed by the slow addition of Et_3SiH (11.5 mL, 72 mmol, 3.0 eq). The reaction mixture was stirred for additional 2.5 hours at -80 °C. After the reaction was completed, the extraction was carried out with DCM, the combined organic layers were dried over Na_2SO_4 and the solvent was removed under reduced pressure. The residue was purified by column chromatography (Cy:EA 19:1 – 4:1 – 1:1 with 0.5% Et_3N). Compound **4** was obtained as a purple oil (13.41 g, 20.16 mmol, 84%).

TLC: R_f = 0.89 (cyclohexane/ethyl acetate 3:1)

^{1}H-NMR: (500 MHz, $CDCl_3$-*d*) δ (ppm) = 7.71 (dd, *J* = 7.8, 1.2 Hz, 1H, Ar *H*-6), 7.39 (d, *J* = 7.1 Hz, 1H, Ar *H*-4), 7.35 – 7.31 (m, 1H, Ar *H*-5), 6.38 (s, 1H, Ar C*H*), 5.36 (t, *J* = 7.5 Hz, 1H, 1'-*H*), 4.61 – 4.55 (m, 1H, 3'-*H*), 4.22 (dd, *J* = 11.5, 3.5 Hz, 1H, 5'-C*H2*), 4.04 (dd, *J* = 11.5, 7.6 Hz, 1H, 5'-C*H2*), 3.97 (ddd, *J* = 7.6, 5.3, 3.5 Hz, 1H, 4'-*H*), 2.44 (ddd, *J* = 12.7, 7.2, 4.6 Hz, 1H, 2'-C*H2*), 2.32 (dt, *J* = 12.9, 7.9 Hz, 1H, 2'-C*H2*), 1.35 (dt, *J* = 14.9, 7.5 Hz, 3H, TIPS-C*H*), 1.16 – 1.01 (m, 49H, TIPS-C*H3*+TIPDS-C*H*+TIPDS-C*H3*).

^{13}C{^{1}H}-NMR: (126 MHz, DMSO-*d6*) δ (ppm) = 147.38, 136.20, 134.99, 133.97, 124.02, 122.06, 120.73, 102.50, 86.63, 78.34, 73.69, 68.13, 63.87, 41.55, 25.76, 18.12, 17.76, 17.66, 17.60, 17.59, 17.41, 17.27, 17.24, 17.16, 13.63, 13.55, 13.20, 12.78, 12.75.

^{29}Si-INEPT-NMR: (99 MHz, DMSO-*d6*) δ (ppm) = 17.66, -12.42.

MALDI-HRMS: *m/z* calculated for $C_{34}H_{60}O_5SSi_3$ [M+Na]$^+$ 687.33615; found 687.33540 (Δ_m = 0.00075, Δ_m/m = 1.1 ppm).

7-((4*S*,5*R*)-4-hydroxy-5-(hydroxymethyl)tetrahydrofuran-2-yl)benzo[b]thiophen-3(2*H*)-one (5)

Compound **4** (13.00 g, 19.54 mmol, 1.0 eq) was dissolved in 10 mL dry THF. Subsequently, 1 M TBAF solution in THF (58.6 mL, 58.6 mmol, 3.0 eq) was added, and the reaction mixture was stirred for 3 hours at room temperature. $CaCO_3$ (1.38 g), DOWEX (4.14 g) and 2 mL of dry MeOH were added under continuous stirring, and the reaction mixture was stirred for additional 1.5 hours at room temperature. The reaction was stopped by addition of EA followed by the filtration over Celite and the removal

of the solvent under reduced pressure. The residue was extracted with EA, the combined organic layers were washed 5 times with saturated NH_4Cl solution, dried over Na_2SO_4 and the solvent was removed under reduced pressure. The crude product was purified by column chromatography (EA:MeOH 19:1 with 0.5% Et_3N). Compound **5** was obtained as a purple foam (3.58 g, 13.43 mmol, 86%).

TLC: R_f = 0.55 (ethyl acetate/methanol 9:1)

^{1}H-NMR: (500 MHz, DMSO-*d$_6$*) δ (ppm) = 10.06 (s, 1H, Enol-O*H*), 7.64 (dd, *J* = 7.9, 1.2 Hz, 1H, Ar *H*-6), 7.43 (d, *J* = 7.2 Hz, 1H, Ar *H*-4), 7.34 (t, *J* = 7.6 Hz, 1H, Ar *H*-5), 6.44 (s, 1H, Ar-C*H*), 5.26 (dd, *J* = 10.4, 5.5 Hz, 1H, 1'-*H*), 5.15 (d, *J* = 3.7 Hz, 1H, 3'-O*H*), 4.79 (d, *J* = 5.4 Hz, 1H, 5'-O*H*), 4.23 (d, *J* = 2.0 Hz, 1H, 3'-*H*), 3.88 – 3.82 (m, 1H, 4'-*H*), 3.58 (dd, *J* = 10.7, 5.6 Hz, 1H, 5'-C*H$_2$*), 3.50 (dd, *J* = 11.3, 5.6 Hz, 1H, 5'-C*H$_2$*), 2.21 (ddd, *J* = 12.7, 5.5, 1.6 Hz, 1H, 2'-C*H$_2$*), 1.89 (ddd, *J* = 12.7, 10.4, 5.7 Hz, 1H, 2'-C*H$_2$*).

^{13}C{^{1}H}-NMR: (126 MHz, DMSO-*d$_6$*) δ (ppm) = 148.65, 136.69, 134.25, 132.85, 123.81, 121.24, 119.69, 97.82, 87.90, 77.69, 72.35, 62.42, 41.64.

ESI-HRMS: *m/z* calculated for $C_{13}H_{14}O_4S$ $[M+H]^+$ 267.06856; found 267.0673 (Δ_m = 0,00126, Δ_m/m = 4.8 ppm).

(*Z*)-2-((4-(dimethylamino)phenyl)imino)-7-((4*S*,5*R*)-4-hydroxy-5-(hydroxymethyl)tetrahydrofuran-2-yl)benzo[b]thiophen-3(2*H*)-one (1a)

Compound **5** (1.09 g, 4.09 mmol, 1.0 eq) and *N,N*-dimethyl-4-nitrosoanilin (DMA-NO) (0.63 g, 4.2 mmol, 1.05 eq) were dissolved in 19 mL ethanol. The addition of 17 mL 1M NaOH was carried out at room temperature and the reaction mixture was stirred for 19 hours. The reaction was stopped by removal of the solvent under reduced pressure. The residue was extracted with DCM, the combined organic layers were washed with brine and subsequently dried over Na_2SO_4 and the solvent was removed under reduced pressure. The crude product was purified by column chromatography (DCM:MeOH 40:1 – 10:1 with 0.5% Et_3N). Compound **1a** was obtained as a dark red solid (0.98 g, 2.5 mmol, 60%).

TLC: R_f = 0.39 (dichloromethane/methanol 40:1)

^{1}H-NMR: (400 MHz, DMSO-*d$_6$*) δ (ppm) = 7.86 (d, *J* = 7.4 Hz, 1H, Ar *H*-6), 7.80 – 7.75 (m, 1H, Ar *H*-4), 7.48 – 7.40 (m, 3H, Ar *H*-5+DMA Ar-C*H*), 6.90 (dd, *J* = 7.3, 5.2 Hz, 2H, DMA Ar-C*H*), 5.21 (d, *J* = 4.0 Hz, 1H, 3'-O*H*), 5.11 (dd, *J* = 10.4, 5.5 Hz, 1H, 1'-*H*), 4.84 (t, *J* = 5.5 Hz, 1H, 5'-O*H*), 4.25 (s, 1H, 3'-*H*), 3.86 (td, *J* = 5.3, 2.0 Hz, 1H, 4'-*H*), 3.62 – 3.45 (m, 2H, 5'-C*H$_2$*), 3.04 (s, 6H, DMA-C*H$_3$*), 2.18 (dd, *J* = 12.2, 6.0 Hz, 1H, 2'-C*H$_2$*), 1.84 (ddd, *J* = 12.6, 10.4, 5.5 Hz, 1H, 2'-C*H$_2$*).

^{13}C{^{1}H}-NMR: (101 MHz, DMSO-*d$_6$*) δ (ppm) = 185.34, 151.21, 146.05, 140.89, 138.66, 135.30, 133.83, 128.13, 127.26, 126.77, 126.18, 112.77, 88.67, 77.20, 72.80, 62.64, 42.11.

ESI-HRMS: *m/z* calculated for $C_{21}H_{22}N_2O_4S$ $[M+H]^+$ 399.1373; found 399.1380 (Δ_m = 0.0007, Δ_m/m = 1.8 ppm).

(*Z*)-7-((4*S*,5*R*)-4-hydroxy-5-(hydroxymethyl)tetrahydrofuran-2-yl)-2-((2,3,6,7-tetrahydro-1*H*,5*H*-pyrido[3,2,1-*ij*]quinolin-9-yl)imino)benzo[*b*]thiophen-3(2*H*)-one (1b)

Compound **5** (0.55 g, 2.1 mmol, 1.0 eq) and 9-Nitroso-2,3,6,7-tetrahydro-1*H*,5*H*-pyrido[3,2,1-*ij*]quinoline (J-NO) (0.42 g, 2.1 mmol, 1.0 eq) were dissolved in 19 mL ethanol. The addition of 1.9 mL 1 M NaOH was carried out at room temperature, and the reaction mixture was stirred for 1.5 hours. The solvent was removed under reduced pressure. The residue was extracted with DCM, the combined organic layers were washed with brine and subsequently dried over Na_2SO_4 and the solvent was removed under reduced pressure. The crude product was purified by column chromatography (DCM:MeOH 19:1 – 9:1 with 0.5% Et_3N). Compound **1b** was obtained as a deep blue solid (0.54 g, 1.2 mmol, 58%).

TLC: R_f = 0.20 (dichloromethane/methanol 19:1)

^{1}H-NMR: (500 MHz, DMSO-*d$_6$*) δ (ppm) = 7.81 (d, *J* = 7.6 Hz, 1H, Ar *H*-6), 7.75 (d, *J* = 7.5 Hz, 1H, Ar *H*-4), 7.41 (t, *J* = 7.6 Hz, 1H, Ar *H*-5), 7.04 (s, 2H, J Ar-C*H$_2$*), 5.21 (d, *J* = 3.9 Hz, 1H, 3'-O*H*), 5.14 (dd, *J* = 10.4, 5.5 Hz, 1H, 1'-*H*), 4.83 (t, *J* = 5.6 Hz, 1H, 5'-O*H*), 4.25 (s, 1H, 3'-*H*), 3.87 (dd, *J* = 7.0, 3.4 Hz, 1H, 4'-*H*), 3.63 – 3.48 (m, 2H, 5'-C*H$_2$*), 3.30 – 3.24 (m, 4H, J-C*H$_2$*), 2.75 (t, *J* = 6.2 Hz, 4H, J-C*H$_2$*), 2.17 (dd, *J* = 12.9, 5.5 Hz, 1H, 2'-C*H$_2$*), 1.93 – 1.85 (m, 5H, J-C*H$_2$*+2'-C*H$_2$*).

^{13}C{^{1}H}-NMR: (126 MHz, DMSO-*d$_6$*) δ (ppm) = 184.72, 144.13, 143.18, 140.32, 137.92, 133.36, 133.33, 127.99, 126.55, 125.54, 124.44, 121.27, 88.32, 77.07, 72.31, 62.20, 49.30, 45.72, 41.69, 27.22, 20.90.

MALDI-HRMS: *m/z* calculated for $C_{25}H_{26}N_2O_4S$ $[M+H]^+$ 451.16860; found 451.16720 (Δ_m = 0.00140, Δ_m/m = 3.1 ppm).

(*Z*)-7-((4*S*,5*R*)-5-((bis(4-methoxyphenyl)(phenyl)methoxy)methyl)-4-hydroxytetrahydrofuran-2-yl)-2-((4-(dimethylamino) phenyl)imino)benzo[*b*]thiophen-3(2*H*)-one (6a)

Compound **1a** (0.20 g, 0.50 mmol, 1.0 eq) was initially coevaporated 3 times using dry pyridine and subsequently dissolved in 6 mL dry pyridine. A spatula tip of DMAP was added and the solution was cooled to 0 °C. DMTCl (0.19 g, 0.55 mmol, 1.1 eq) in 5 mL dry pyridine was slowly added at 0 °C over 30 minutes. The reaction mixture was stirred for 2 days at room temperature in which subsequently DMTCl (0.43 g, 1.7 mmol, 2.5 eq) was added at 0°C and stirred again at room temperature for 18 hours. The reaction was stopped by addition of 2 mL of MeOH and the reaction mixture was coevaporated with toluene. The residue was extracted with DCM, the combined organic layers were dried over Na_2SO_4 and the solvent was removed under reduced pressure. The crude product was purified by column chromatography (EA:Cy 3:1 – 100% EA – EA:MeOH 50:1 with 0.5% Et_3N). Compound **6a** was obtained as a purple-red solid (0.12 g, 0.17 mmol, 34%).

TLC: R_f = 0.63 (100% ethyl acetate)

^{1}H-NMR: (400 MHz, DMSO-*d$_6$*) δ (ppm) = 7.81 – 7.74 (m, 2H, Ar *H*-6+Ar *H*-4), 7.44 – 7.36 (m, 5H, Ar *H*-5+DMA Ar-C*H*+Ar-*H*), 7.27 (dt, *J* = 9.0, 4.5 Hz, 6H, Ar-*H*), 7.19 (d, *J* = 7.2 Hz, 1H, Ar-*H*), 6.84 (d, *J* = 8.7 Hz, 4H, Ar-*H*), 6.78 – 6.74 (m, 2H, DMA Ar-C*H*), 5.27 (s, 1H, 1'-*H*), 5.18 (dd, *J* = 9.8, 5.8 Hz, 1H, 3'-O*H*), 4.18 (s, 1H, 4'-*H*), 3.99 (dd, *J* = 7.7, 4.7 Hz, 1H, 3'-*H*), 3.71 (d, *J* = 3.2 Hz, 6H, DMT O-C*H$_3$*), 3.18 (d, *J* = 4.6 Hz, 2H, 5'-C*H$_2$*), 3.00 (s, 6H, DMA-C*H$_3$*), 2.27 – 2.20 (m, 1H, 2'-C*H$_2$*), 1.96 – 1.88 (m, 1H, 2'-C*H$_2$*).

^{13}C{^{1}H}-NMR: (101 MHz, DMSO-*d$_6$*) δ (ppm) = 158.04, 150.66, 145.68, 140.57, 135.59, 134.78, 129.73, 127.79, 127.72, 126.69, 126.22, 113.14, 112.18, 86.37, 85.43, 77.08, 72.17, 64.04, 54.99.

ESI-HRMS: *m/z* calculated for $C_{42}H_{40}N_2O_6S$ $[M+H]^+$ 701.2680; found 701.2691 (Δ_m = 0.0011, Δ_m/m = 1.6 ppm).

(*Z*)-7-((4*S*,5*R*)-5-((bis(4-methoxyphenyl)(phenyl)methoxy)methyl)-4-hydroxytetrahydrofuran-2-yl)-2-((2,3,6,7-tetrahydro-1*H*,5*H*-pyrido[3,2,1-*ij*]quinolin-9-yl)imino)benzo[*b*]thiophen-3(2*H*)-one (6b)

Compound **1b** (0.35 g, 0.78 mmol, 1.0 eq) was initially coevaporated 3 times using dry pyridine and subsequently dissolved in 6 mL dry pyridine. A spatula tip of DMAP was added and the solution was cooled to 0 °C. DMTCl (0.29 g, 0.86 mmol, 1.1 eq) in 5 mL dry pyridine was slowly added at 0 °C over 30 minutes. The reaction mixture was stirred for additional 4 hours at room temperature. Subsequently, DMTCl (0.13 g, 0.39 mmol, 0.5 eq) was added again at 0 °C and the reaction mixture was stirred overnight at room temperature. The reaction was stopped by addition of 2 mL of MeOH and the reaction mixture was coevaporated with toluene. The residue was extracted with DCM, the combined organic layers were dried over Na_2SO_4 and the solvent was removed under reduced pressure. The crude product was purified by column chromatography (Cy:EA 1:1 – EE:MeOH 9:1 with 0.5% Et_3N). Compound **6b** was obtained as a blue foam (0.26 g, 0.34 mmol, 44%).

TLC: R_f = 0.45 (cyclohexane/ethyl acetate 1:1)

^{1}H-NMR: (500 MHz, DMSO-*d$_6$*) δ (ppm) = 7.79 – 7.73 (m, 2H, Ar *H*-6+ Ar *H*-4), 7.40 (t, *J* = 7.9 Hz, 3H, Ar-*H*), 7.27 (dd, *J* = 14.2, 6.4 Hz, 6H, Ar-*H*, Ar *H*-5), 7.19 (t, *J* = 7.2 Hz, 1H, Ar-*H*), 7.00 (s, 2H, J Ar-C*H*), 6.84 (d, *J* = 8.2 Hz, 4H, Ar-*H*), 5.28 (d, *J* = 4.3 Hz, 1H, 3'-O*H*), 5.21 (dd, *J* = 9.6, 6.3 Hz, 1H, 1'-*H*), 4.15 (d, *J* = 3.1 Hz, 1H, 3'-*H*), 4.00 (dd, *J* = 5.6, 3.2 Hz, 1H, 4'-*H*), 3.69 (d, *J* = 6.2 Hz, 6H, DMT O-C*H$_3$*), 3.26 (t, *J* = 5.6 Hz, 5H, J-C*H$_2$*+5'-C*H$_2$*), 3.13 (dd, *J* = 9.9, 3.6 Hz, 1H, 5'-C*H$_2$*), 2.64 (dd, *J* = 10.1, 5.6 Hz, 4H, J-C*H$_2$*), 2.24 (dd, *J* = 11.7, 6.2 Hz, 1H, 2'-C*H$_2$*), 1.97 – 1.91 (m, 1H, 2'-C*H$_2$*), 1.89 – 1.83 (m, 4H, J-C*H$_2$*).

^{13}C{^{1}H}-NMR: (126 MHz, DMSO-*d$_6$*) δ (ppm) = 184.68, 170.32, 158.03, 158.02, 144.94, 144.11, 143.00, 140.58, 137.82, 135.60, 135.51, 133.29, 133.00, 129.70, 129.67, 128.14, 127.79, 127.67, 126.63, 126.48, 125.59, 124.46, 121.21, 113.16, 113.14, 86.26, 85.40, 77.13, 72.24, 64.21, 59.73, 54.96, 54.94, 49.28, 41.62, 27.17, 20.88, 20.74, 14.07.

MALDI-HRMS: *m/z* calculated for $C_{46}H_{44}N_2O_6S$ $[M+H]^+$ 753.29928; found 753.29654 (Δ_m = 0.00274, Δ_m/m = 3.7 ppm).

(2*R*,3*S*)-2-((bis(4-methoxyphenyl)(phenyl)methoxy)methyl)-5-((*Z*)-2-((4-(dimethylamino)phenyl)imino)-3-oxo-2,3-dihydrobenzo[*b*]thiophen-7-yl)tetrahydrofuran-3-yl(2-cyanoethyl)diisopropylphosphoramidite (7a)

Compound **6a** (0.12 g, 0.17 mmol, 1.0 eq) was dissolved in 1 mL dry DCM. Subsequently, DIPEA (0.15 g, 0.84 mmol, 5.0 eq) and CEO-(N^{i}Pr$_2$)Cl (0.056 g, 0.25 mmol, 1.5 eq) were added at room temperature and the reaction mixture was stirred for 20 hours.

The extraction was performed with DCM and saturated $NaHCO_3$ solution, the combined organic layers were dried over Na_2SO_4 and the solvent was removed under reduced pressure. The crude product was purified by column chromatography (Cy:EA 4:1 – 1:1 with 0.5% Et_3N). Compound **7a** was obtained as a red solid (0.066 g, 0.073 mmol, 44%).

TLC: R_f = 0.61 (cyclohexane/ethyl acetate 1:1)

^{1}H-NMR: (500 MHz, DMSO-*d6*) δ (ppm) = 7.80 (ddd, *J* = 12.9, 7.1, 4.4 Hz, 2H, Ar *H*-6+ Ar *H*-4), 7.46 – 7.39 (m, 3H, Ar *H*-5+Ar-*H*), 7.36 (dd, *J* = 9.1, 0.8 Hz, 2H, DMA Ar-C*H*), 7.30 – 7.22 (m, 6H, Ar-*H*), 7.21 – 7.16 (m, 1H, Ar-*H*), 6.85 – 6.80 (m, 4H, Ar-*H*), 6.72 – 6.66 (m, 2H, DMA Ar-C*H*), 5.22 – 5.13 (m, 1H, 1'-*H*), 4.49 – 4.40 (m, 1H, 3'-*H*), 4.16 – 4.05 (m, 1H, 4'-*H*), 3.74 – 3.70 (m, 2H, CEO-C*H2*), 3.67 – 3.61 (m, 3H, DMT O-C*H3*), 3.31 – 3.19 (m, 2H, 5'-C*H2*), 2.98 (d, *J* = 1.5 Hz, 6H, DMA-C*H3*), 2.76 (t, *J* = 5.9 Hz, 1H, CEO-C*H2*), 2.68 (t, *J* = 5.9 Hz, 1H, CEO-C*H2*), 2.49 – 2.35 (m, 1H, 2'-C*H2*), 2.12 – 2.03 (m, 1H, 2'-C*H2*), 1.25 – 1.07 (m, 12H, *i*Pr-C*H3*), 1.01 (d, *J* = 6.8 Hz, 3H, *i*Pr-C*H*).

^{13}C{^{1}H}-NMR: (126 MHz, DMSO-*d6*) δ (ppm) = 184.82, 184.79, 158.09, 150.62, 145.60, 145.57, 144.79, 140.69, 140.59, 137.12, 135.49, 135.45, 135.42, 134.75, 133.31, 133.19, 129.72, 129.70, 128.03, 128.00, 127.97, 127.80, 127.71, 127.66, 126.72, 126.61, 126.41, 126.24, 126.22, 125.94, 118.94, 118.82, 113.14, 112.07, 85.62, 85.58, 85.14, 77.37, 74.77, 74.68, 63.39, 62.88, 59.76, 58.44, 58.40, 58.30, 58.26, 55.00, 54.99, 42.84, 42.68, 42.65, 42.59, 42.55, 31.30, 29.62, 26.34, 26.31, 24.37, 24.33, 24.29, 24.24, 24.18, 22.10, 20.77.

^{31}P{^{1}H}-NMR: (202 MHz, DMSO-*d6*) δ (ppm) = 147.42, 147.00.

ESI-HRMS: *m/z* calculated for $C_{51}H_{57}N_4O_7PS$ $[M+H]^+$ 901.3758; found 901.3764 (Δ_m = 0.0006, Δ_m/m = 0.7 ppm).

(2*R*,3*S*)-2-((bis(4-methoxyphenyl)(phenyl)methoxy)methyl)-5-((*Z*)-3-oxo-2-((2,3,6,7-tetrahydro-1*H*,5*H*-pyrido[3,2,1-*ij*]quinolin-9-yl)imino)-2,3-dihydrobenzo[*b*]thiophen-7-yl)tetrahydrofuran-3-yl(2-cyanoethyl)diisopropylphosphoramidite (7b)

Compound **6b** (0.24 g, 0.32 mmol, 1.0 eq) was dissolved in 2.5 mL dry DCM. Subsequently, DIPEA (0.21 g, 1.6 mmol, 5.0 eq) and CEO-(^{i}NPr$_2$)Cl (0.12 g, 0.49 mmol, 1.5 eq) were added at room temperature and the reaction mixture was stirred for 3 hours. The extraction was performed with DCM and $NaHCO_3$ solution, the combined organic layers were dried over Na_2SO_4 and the solvent was removed under reduced pressure. The crude product was purified by column chromatography (Cy:EA 2:1 – 1:1 – 1:2 with 0.5% Et_3N). Compound **7b** was obtained as a blue foam (0.28 g, 0.30 mmol, 92%).

TLC: R_f = 0.45 (cyclohexane/ethyl acetate 2:1)

^{1}H-NMR: (500 MHz, DMSO-*d6*) δ (ppm) = 7.77 (t, *J* = 8.5 Hz, 2H, Ar *H*-6+ Ar *H*-4), 7.44 – 7.38 (m, 3H, Ar *H*-5+ Ar-*H*), 7.29 – 7.23 (m, 6H, Ar-*H*), 7.22 – 7.16 (m, 1H, Ar-*H*), 7.00 (s, 2H, J-C*H*), 6.83 (dd, *J* = 14.2, 5.5 Hz, 4H, Ar-*H*), 5.22 (ddd, *J* = 16.0, 9.7, 5.9 Hz, 1H, 1'-*H*), 4.49 – 4.33 (m, 1H, 3'-*H*), 4.13 (dd, *J* = 22.3, 3.7 Hz, 1H, 4'-*H*), 3.78 – 3.71 (m, 1H, CEO-C*H2*), 3.70 – 3.67 (m, 6H, DMT O-C*H3*), 3.64 (dd, *J* = 13.4, 6.4 Hz, 1H, CEO-C*H2*), 3.27 (d, *J* = 4.7 Hz, 5H, J-C*H2*+5'-C*H2*), 3.22 – 3.14 (m, 1H, 5'-C*H2*), 2.75 (t, *J* = 5.8 Hz, 1H, CEO-C*H2*), 2.63 (ddd, *J* = 18.7, 12.3, 6.0 Hz, 5H, J-C*H2*+CEO-C*H2*), 2.46 – 2.34 (m, 1H, 5'-C*H2*), 2.12 – 2.01 (m, 1H, 2'-C*H2*), 1.92 – 1.81 (m, 4H, J-C*H2*), 1.14 (ddd, *J* = 9.8, 8.9, 5.0 Hz, 12H, *i*Pr-C*H3*), 1.02 (d, *J* = 6.7 Hz, 2H, *i*Pr-C*H*).

^{13}C{^{1}H}-NMR: (126 MHz, DMSO-*d6*) δ (ppm) = 184.62, 184.56, 170.32, 158.08, 144.82, 144.14, 142.86, 142.65, 140.66, 140.48, 137.10, 137.03, 135.46, 135.43, 135.34, 133.25, 133.20, 133.04, 132.79, 129.66, 129.61, 128.22, 128.13, 127.81, 127.65, 127.59, 126.65, 126.59, 125.76, 124.52, 124.46, 121.18, 121.17, 118.86, 118.76, 113.17, 85.62, 85.56, 84.93, 77.33, 77.19, 74.87, 74.73, 63.68, 63.58, 59.73, 58.38, 58.26, 54.98, 54.96, 54.93, 54.89, 49.27, 42.72, 42.66, 42.62, 42.56, 27.19, 27.16, 24.31, 24.25, 24.22, 24.16, 20.87, 20.74, 19.86, 19.80, 19.73, 14.07.

^{31}P{^{1}H}-NMR: (202 MHz, DMSO-*d6*) δ (ppm) = 147.44, 147.22.

MALDI-HRMS: *m/z* calculated for $C_{55}H_{61}N_4O_7PS$ $[M+H]^+$ 953.40713 found 953.40576 (Δ_m = 0.00137, Δ_m/m = 1.5 ppm).

2. Anomeric Configuration of the Modified Nucleoside

In the synthesis of nucleoside analogues, it is essential that the native structure, size and orientation of the natural nucleobase is mimicked as closely as possible, thus minimizing perturbation of native structures.

With regard to the loss of stereochemical control due to the ring opening associated with the addition reaction to obtain compound **3**, the anomeric ratio resulting from the dehydroxylation was analyzed using 1D and 2D NMR spectroscopy. The recording of a ROESY spectrum enables the spatial proximity of the protons in the 1'-, 2'- and 3'-positions as well as the anomeric ratio to be determined. The measurement of the free BTP nucleoside **5** shown in Fig. S1 shows pronounced cross-coupling of the 1'- and 2'-position (red and green marked) and the 2'- and 3'-position (purple and yellow marked) and are attributed to the targeted β-anomer. The nucleoside analogue thus has the orientation of natural nucleobases, minimizing the potential for interference. By integrating the signals in the ^{1}H spectrum associated with ROESY, it was possible to determine an α-content of 10%.

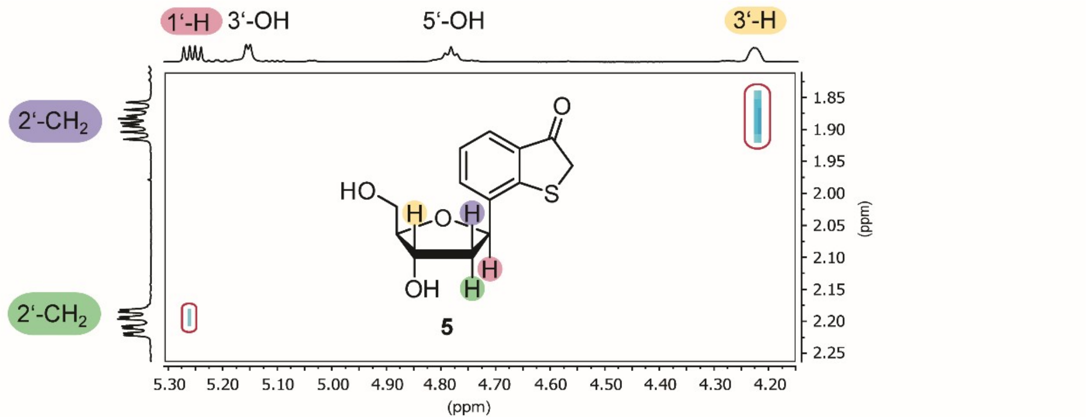

Fig. S1 ROESY 2D-NMR data: Characterization of the anomeric conformation and ratio of compound **5**. The protons of interest (1'-, 2'- and 3'-positions) are color-coded corresponding to their NMR signal.

In addition, we were able to characterize the conformation of the deoxyribose based on the signal patterns and the corresponding *J*-couplings of the 1'H positions of both anomers. The magnified region of the respective ^{1}H-NMR is shown in Fig. S2. Both anomers have an equivalent signal pattern with two coupling constants of 10.0 / 10.5 Hz and 5.5 Hz. According to the Karplus relationships, these are due to a syn-clinal and an anti-periplanar coupling. For the β-anomer, these relationships are possible in the 2' endo conformation of the sugar moiety. This is favored since the sterically demanding BTP chromophore is located in an energetically preferred equatorial orientation. For the α-anomer, on the other hand, the 3' endo conformation is favored, as the BTP is also in an equatorial orientation, also steric repulsion between BTP and 3'OH is prevented in comparison to 2' endo.

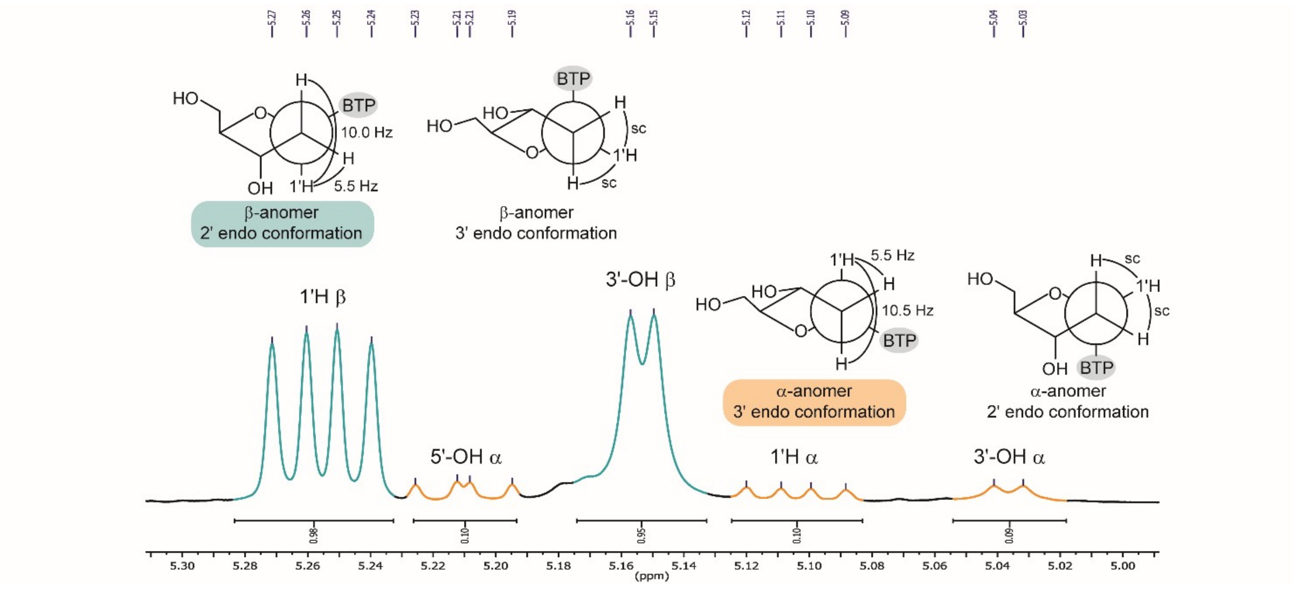

Fig. S2 Magnified region of the ^{1}H-NMR data of compound **5** in DMSO-d_6 including signal assignments. The determination of the sugar conformation was performed based on the *J*-couplings of the 1'H signal (Karplus relations) and is visualized using Newman-projections of both anomeric structures.

3. Photochemical Experiments (UV/Vis)

3.1. Absorption Spectra / Extinction Coefficients

The extinction coefficients of dipeptides **DMA-ITI-*C*-Nucleoside 1a** and **J-ITI-*C*-Nucleoside 1b** were determined on a *Specord S600* UV/Vis spectrometer (*Jena Analytik*) using quartz glass cuvettes with a light path of 10.00 mm (*Hellma Analytics*). For this purpose, a dilution series of the respective molecules in the optical density range between 0.1 and 1 was prepared. Sample preparation was performed using p.a. grade solvent MeOH or 1x PBS buffer. The data sets of all recorded spectra were baseline corrected by subtracting the absorption minimum in a region where no absorption was detectable (800-900 nm). The absorbance at the absorption maximum of the compound was plotted against the concentration and the interception was set for (0.0) for linear fitting. The extinction coefficients were calculated according to Beer-Lambert law (Fig. S3 left, Table S1). The additional plot of the extinction coefficient against the absorption wavelength serves as a representation of its solvent dependence (Fig. S3 right, Table S1).

a) DMA-ITI-*C*-Nucleoside 1a

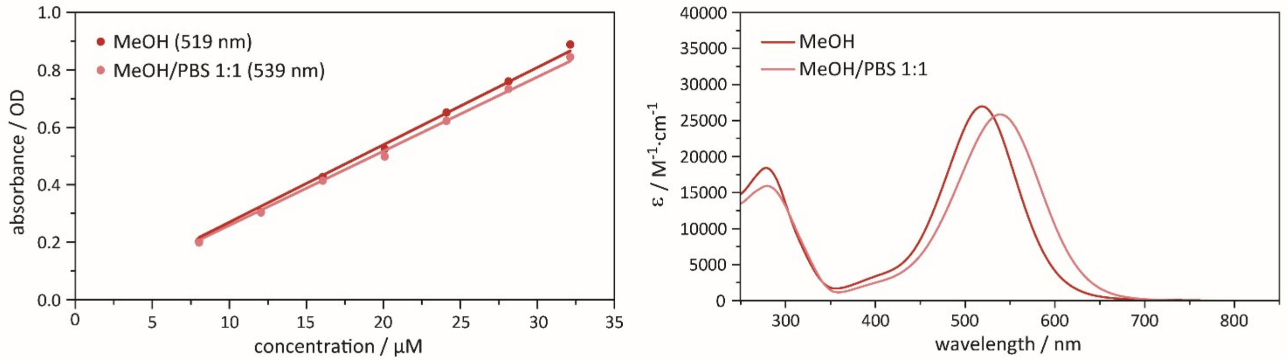

b) J-ITI-*C*-Nucleoside 1b

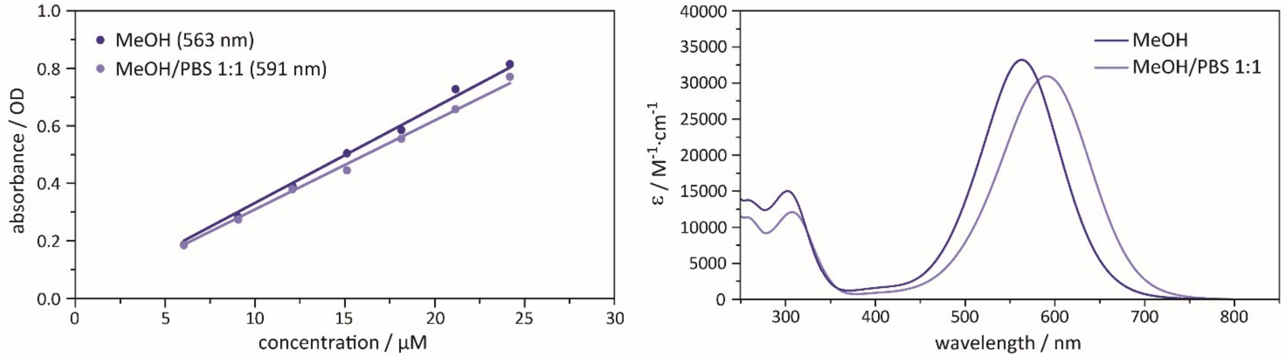

Fig. S3 Absorption measurements of (a) DMA-ITI-*C*-Nucleoside **1a** and (b) J-ITI-*C*-Nucleoside **1b** in MeOH, MeOH/PBS 1:1. Left: Plot of dilution series with corresponding linear fitting. Right: Absorption spectra including the extinction coefficients.

Table S1 Solvent dependent extinction coefficients of DMA-ITI-*C*-Nucleoside **1a** and J-ITI-*C*-Nucleoside **1b** in MeOH, MeOH/PBS 1:1.

DMA-ITI-*C*-Nucleoside **1a**	$\lambda_{abs.\ max.}$ / nm	ε / $M^{-1}\cdot cm^{-1}$	J-ITI-*C*-Nucleoside **1b**	$\lambda_{abs.\ max.}$ / nm	ε / $M^{-1}\cdot cm^{-1}$
MeOH	519	27 000 ± 300	MeOH	563	33 200 ± 400
MeOH/PBS (1:1)	539	25 900 ± 300	MeOH/PBS (1:1)	591	31 000 ± 400

3.2. Hypochromicity of DMA- and J-ITI Chromophores in double stranded DNA

For further evaluation of the π-stacking interactions between the quencher chromophore and the adjacent nucleobases of the native oligonucleotide structure, additional hypochromicity studies were performed. For this purpose, the temperature-dependence of the absorption properties of internally DMA-ITI- and J-ITI-modified oligonucleotides ON_{int}^{DMA} and ON_{int}^{J} was characterized. The absorption of both oligonucleotides was measured in 1 x PBS buffer at 20, 40, 60 and 80 °C. The measurements were accomplished on a *Specord S600* UV/Vis spectrometer (*Jena Analytik*) using quartz glass cuvettes with a light path of 10.00 mm (*Hellma Analytics*). The data sets of all recorded spectra were baseline corrected by subtracting the absorption minimum in a region where no absorption was detectable (800-900 nm). To highlight the change of the absorption maxima, all spectra were normalized and plotted together for all temperatures of each sample (Fig. S4).

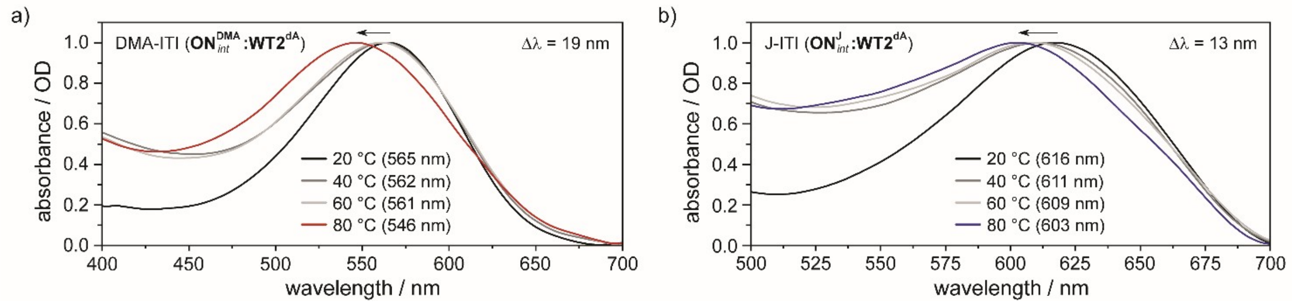

Fig. S4 Temperature-dependent changes of the absorption of internally DMA-ITI- and J-ITI-modified oligonucleotides indicating π-stacking between the quencher chromophore and the adjacent nucleobases of the native DNA duplex structure.

Fig. S4 clearly shows a temperature-dependence of the chromophore absorption properties for both ITI-derivatives. Starting with an absorption maximum at 565 nm for the DMA-ITI and 616 nm for the J-ITI at 20 °C, increasing the temperature to 80 °C gradually induces hypsochromic shifts of the absorption maxima for both derivatives. At 80 °C the absorption maximum of the DMA-ITI is shifted 19 nm to 546 nm and the J-ITI shifts 13 nm to 603 nm. It is well known for organic compounds, that increased π-interaction or a larger π-system induces bathochromic shifts while a decrease results in hypsochromic shift of the absorption properties. Also, a temperature increase of oligonucleotides in solution leads to a complete or partial weakening and dissociation of the native oligonucleotide duplex structure. Our measurements show a significant hypsochromic shift with increasing sample temperature with especially pronounced effects above the melting temperatures of the oligonucleotides. The observed findings display a hypsochromic shift of the chromophore absorption properties with decreasing native oligonucleotide duplex interactions at higher temperatures. This indicates decreased π-interaction between the chromophore structure and the adjacent nucleobases compared to the conditions at ambient temperature providing yet another experiment that supports our hypothesis of strong positive interactions, like π-stacking within our quencher-modified oligonucleotides.

3.3. Determination of the Förster Distance R_0

The Förster distances R_0 of the F/Q-pairs (Cy3/ DMA-ITI, Cy3/ BHQ-2, ATTO Rho11/ J-ITI and ATTO Rho11/ BHQ-2) were determined according to the following procedure based on the absorption spectra of DMA- and J-ITI-*C*-nucleoside **1a** und **1b** as well as the BHQ2 absorption spectrum and the Cy3 and ATTO Rho11 emission spectra (obtained from www.fpbase.org/spectra).[3]

1. The absorption spectrum of the quencher was normalized and multiplied by the molar extinction coefficient, resulting in the molar extinction coefficient as a function of the wavelength $\varepsilon_A(\lambda)$.

2. The fluorophore emission spectrum $F_D(\lambda)$ was area-normalised to a value of 1 as a function of the wavelength according to equation (1).

$$(1) \quad F_D(\lambda) = \frac{F_D(\lambda)}{\int_0^\infty F_D(\lambda) d\lambda}$$

3. The spectral overlap integral of F/Q-pairs was determined according to equation (2).

$$(2) \quad J = \int_0^\infty F_D(\lambda)\varepsilon_A(\lambda)\lambda^4 d\lambda$$

4. Subsequently, the spectral overlap integral J is used to determine the Förster distance R_0 based on equation (3) with N_A (Avogadro constant), ϕ_D(donor quantum yield), n (refractive factor of the medium, used value: 1.333) and κ^2 (dipole orientation factor, used value: $^2/_3$)

$$(3) \quad R_0^6 = \frac{9\ln(10)\,\kappa^2\phi_D J}{128\pi^5 N_A \; n^4}$$

The results obtained for the spectral overlap integrals and the Förster distances are listed in Table S2.

Table S2 Results for overlap integrals J and Förster distances R_0.

F/Q-pair	$\lambda_{max}^{Em.}$ (Fluorophor) / nm	$\lambda_{max}^{Abs.}$ (Quencher) / nm	ε (Quencher) · 10^{-5} / $M^{-1}\cdot cm^{-1}$	Quantum Yield (Fluorophore)	Spectral Overlap Integral J	Förster Radius R_0 / nm
Cy3 + DMA-ITI **1a**	570	539	0.26	0.31	$1.90 \cdot 10^{15}$	4.7
Cy3 + BHQ-2	570	579	0.38	0.31	$3.30 \cdot 10^{15}$	6.1
ATTO Rho11 + J-ITI **1b**	597	591	0.31	0.80	$3.90 \cdot 10^{15}$	5.3
ATTO Rho11 + BHQ-2	597	579	0.38	0.80	$4.24 \cdot 10^{15}$	6.3

4. Ultrafast Vis Measurements

4.1. Materials and Methods

Sample Preparation

Both samples were dissolved and diluted in MeOH and aqueous PBS/MeOH 1:1 mixture to yield an optical density of 0.5 to 0.6 on 1 mm at the respective absorption maximum. 300 μL of these sample solutions were then transferred into a 1 mm quartz cuvette (*Starna*).

Transient Vis-pump / Vis-probe spectroscopy

The time-resolved Vis transient absorption measurements were conducted using a home-built pump-probe setup as described in detail previously.[4] To summarize, a Ti:Sapphire amplifier system (*Clark, MXR-CPA-iSeries*) provided fundamental laser pulses (1 mJ, 775 nm, 130 fs, 1 kHz) that were split into a pump and a probe pathway. Generation of the pump pulses was achieved using a home-built two stage NOPA (noncollinear optical parametric amplifier)[5,6] with a prism compressor located in between the NOPAs for pulse compression. For probing, super continuum probe pulses (300-750 nm) were generated by focusing the laser fundamental into a CaF_2 crystal (5 mm). The resulting beam was split into a probe and reference beam prior to detection. The reference beam was guided directly into a spectrograph (*AMKO Multimode*) containing gratings with 600 grooves/mm blazed at 500 nm and a photodiode array with the detection range set to 430-720 nm. The probe beam was first focused at the sample position, then collected and guided into a second spectrograph of identical configuration. Excitation was conducted at 530 nm and 640 nm (J-ITI, aqueous PBS/MeOH 1:1 mixture) with pulse energies of 90 nJ. The instrument response function of ~70 fs was estimated from the pump probe cross correlation and confirmed using an autocorrelator (*APE, APE Pulse Check*). Experiments were carried out under magic angle conditions (54.7° pump-probe polarization difference) to eliminate anisotropic contributions. To avoid photodegradation the sample cell (fused silica, 1 mm optical path) was constantly moved in the plane perpendicular to the direction of probe pulse propagation.

Data Analysis

Data analysis of the Vis transient pump-probe data was performed using OPTIMUS (http://www.optimusfit.org/).[7] Correction of all datasets for the so-called coherent artefact was achieved using a function composed of a Gaussian and/or its first and second derivative.[7,8] The corresponding fitting was performed within the same routine as the exponential fitting of the data. For analysis, model-independent lifetime distribution analysis (LDA) was applied on all datasets; a method that deals intrinsically with non-exponential kinetics such as cooling dynamics. Here, the pre-exponential amplitudes of a quasi-continuous set (n>50) of exponential functions with fixed, equally spaced (on a decimal logarithm scale) lifetimes are determined. Plotting these pre-exponential amplitudes at each detection wavelength in form of a contour plot yields the displayed lifetime density map (LDM).[9] The reading of LDMs is analogous to the more well-known decay-associated spectra arising from global lifetime analysis. Positive (red) amplitudes account for decay of the excited state and product absorption (ESA and PA) or the rise of ground state bleach and stimulated emission (GSB and SE). Negative (blue) amplitudes describe the rise of ESA and PA or the decay of GSB and SE.

4.2. Results and Discussion

To verify the ultrafast unproductive decay dynamics of the *C*-nucleoside based Iminothioindoxyl compounds in analogy to our previous study on the same chromophore incorporated into an amino acid analogue,[2] time-resolved Vis transient absorption measurements were conducted.

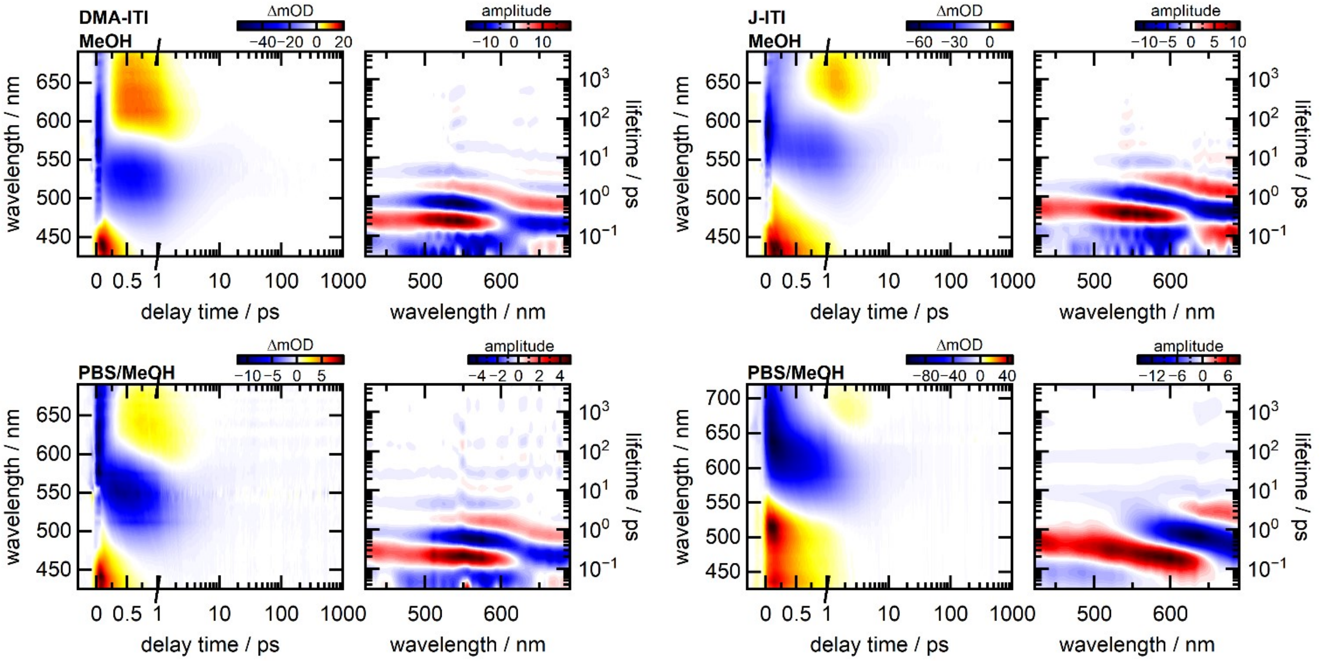

Fig. S5 Transient absorption data and the corresponding LDM obtained by LDA of the DMA-ITI-/ **1a** (left) and J-ITI-C-Nucleoside **1b** (right) in MeOH (top) and aqueous PBS/MeOH 1:1 mixture (bottom). All *C*-nucleosides were excited at 530 nm except for the J-ITI in PBS/MeOH 1:1 mixture where excitation was conducted at 640 nm.

In both MeOH and aqueous PBS/MeOH 1:1 mixture, two negative and two positive contributions are observed in the ultrafast transient absorption data after excitation at 530 nm or 640 nm (J-ITI, aqueous PBS/MeOH 1:1 mixture). In analogy to our previous work,[2] the two negative contributions can be assigned to stimulated emission (SE; >550 nm, J-ITI; >500, DMA-ITI) and ground state bleach (GSB; 550-650 nm, J-ITI; 500-575, DMA-ITI), while the two positive contributions can be assigned to excited state absorption (ESA; <550 nm nm, J-ITI; <475 nm, DMA-ITI) and a hot ground state intermediate (GSI; >650 nm, J-ITI; >575 nm, DMA-ITI). Whereas SE and ESA decay simultaneously after hundreds of fs, the GSI is formed on that timescale. The GSB is then recovered within several ps together with the decay of the GSI. No permanent photoproducts are observed on longer timescales, an important property for efficient quenching.

To identify the underlying kinetics in more detail, lifetime distribution analysis was performed on all datasets. The first pair of positive (>650 nm, DMA-ITI, J-ITI MeOH; >600 nm, J-ITI PBS/MeOH) and negative (<650 nm, DMA-ITI, J-ITI MeOH; <600 nm, J-ITI PBS/MeOH) lifetime distribution amplitudes at ~100 fs can be assigned to a red-shift of the SE connected to the departure from the Franck-Condon region. At ~200-300 fs, the negative lifetime distribution amplitude (>625 nm, DMA-ITI, J-ITI MeOH; >650 nm, J-ITI PBS/MeOH) can be assigned to the decay of the SE and the rise of the hot GSI, while the positive amplitude (<625 nm, DMA-ITI, J-ITI MeOH; <650 nm, J-ITI PBS/MeOH) represents the decay of the ESA. The GSI then decays on the early picosecond timescale and recovers the GS via cooling dynamics, represented by the tilted shape of the positive amplitude (>550 nm, DMA-ITI, J-ITI MeOH; >625 nm, J-ITI PBS/MeOH) elongated in lifetime. While the dynamics in most solvents is remarkably similar with only slight differences in the exact lifetimes, the spectral position of the signals is shifted especially in the J-ITI in PBS/MeOH 1:1 mixture due to the analogous spectral shift of the absorption spectrum in presence of water.

In summary, the ultrafast decay dynamics of the utilized Iminothioindoxyl chromophore compounds are remarkably similar in both *C*-nucleoside and amino acid analogues. In both systems, the excited state decays rapidly via a hot GSI without forming lasting photoproducts, ideal requirements for efficient fluorescence quenching. Based on the similarity of the dynamics compared to the previously studied amino acid analogues, it can be assumed that the excited state dynamics of the *C*-nucleoside compounds are also predominantly independent of the excitation wavelength and the respective solvent polarity. The solvent dependent study in this work further supports this point as the observed effects are identical to the ones in our previous study.[2] These properties render the *C*-nucleoside based Iminothioindoxyl compounds promising candidates as fluorescence quenchers in biological applications.

5. Oligonucleotide Synthesis

5.1. Materials and Methods

All works involving oligonucleotides were carried out using deionized, RNase-free water. Hence, Milli-Q water was stirred over night after addition of 0.1% diethyl pyrocarbonate (DEPC) and autoclaved before usage.

DNA oligonucleotides were prepared using an *ABI 392 DNA/RNA synthesizer (Applied Biosystems)* at 1 µM synthesis scales. 0.3 M of 5-(benzylthio)-1*H*-tetrazole in acetonitrile serving as the activator, UltraMild capping reagents (tetrahydrofuran/ pyridine/ phenoxyacetic anhydride) and a 3% solution of trichloroacetic acid (TCA) in DCM as detritylation reagent were all procured from *emp Biotech*. For oxidation Oxidizing (ABI) reagent was sourced from *J.T. Baker*.

UltraMild-protected phosphoramidites were used for synthesis of all oligonucleotides, acquired through *LGC Biosearch Technologies* (dA (Pac) CE-phosphoramidite, dG (*i*Pr-Pac) CE-phosphoramidite, dT CE-phosphoramidite) and *Glen Research* (dC (Ac) CE-phosphoramidite). The amino-functionalized Fmoc-amino-DMT C-3 CED phosphoramidite for the subsequent introduction of fluorescence-labels was purchased from *ChemGenes*. As quencher reference system BHQ-2-dT CE phosphoramidite was procured through *LGC Biosearch Technologies*. The commercially acquired phosphoramidites were used at 0.1 M concentration in dry ACN (*LGC Biosearch Technologies*). Chromophore-labeled *C*-nucleosidic phosphoramidites were prepared in dry ACN/DCM (1:1) at 0.12 M concentrations. Standard coupling times were utilized for DNA phosphoramidites and the amino-functionalized phosphoramidite, while the coupling times were prolonged to a 12 minutes push cycle for the modified phosphoramidites (molecular structures, Fig. S6). All oligonucleotides besides $\mathbf{ON}^{J}_{int}$ were synthesized in DMT-on mode. DMT-off synthesis strategy is suggested for further oligonucleotide incorporation. Oligonucleotide strands used within this study are shown in Table S3.

Solid supports were used with the first nucleotide already attached to the CPG procured through *LGC Biosearch Technologies (Universal Q Synbase CPG 1000/110, dA-Pac-CPG 1000/110 and dT Synbase CPG 1000/110).*

The unmodified DNA strands **WT1^{dT}**, **WT1^{dA}** and **WT2^{dT}** were purchased HPLC-purified through *Biomers.net GmbH.*

Table S3 Overview of all oligonucleotide sequences. Incorporated modifications include: amino-Modifier (**M**), quencher (**Q**) and fluorophore (**F**). The molecular structure of the Amino-Modifier – Fmoc-amino-DMT C-3 is shown in Fig. S6.

Category	Name	Modification	Sequence (5' → 3')	M_{calc} / Da	M_{found} / Da
Native 1	WT1^{dT}	-	TGC ACC TTT GTT TAT CGA CGT	6375.1268	6376.0066
	WT2^{dA}		ACG TCG ATA AAC AAA GGT GCA	6469.1957	6469.9818
Native 2	WT1^{dA}		TGC ACC TTT GTA TAT CGA CGT	6384.0851	6384.0882
	WT2^{dT}		ACG TCG ATA TAC AAA GGT GCA	6460.1376	6460.2270
Quencher modification	ON^{DMA}_{int}	**7a**	TGC ACC TTT GT**Q** TAT CGA CGT	6531.1654	6531.8577
	ON^{J}_{int}	**7b**		6583.1967	6583.9460
	ON^{BHQ2}_{int}	dT-BHQ2		7033.3610	7034.1702
	ON^{DMA}_{term}	**7a**	**Q** TGC ACC TTT GTT TAT CGA CGT	6835.2142	6835.9594
	ON^{J}_{term}	**7b**		6887.2455	6887.9657
	ON^{BHQ2}_{term}	dT-BHQ2		7337.4071	7338.2806
Amino-modification	ON^{NH2}_{int}	Fmoc-amino-DMT C-3	ACG TCG ATA **M**AC AAA GGT GCA	6309.1583	6309.8964
	ON^{NH2}_{term}		ACG TCG ATA AAC AAA GGT GCA **M**	6622.2176	6622.9908
Fluorophore modification	ON^{Cy3}_{int}	Cy3	ACG TCG ATA **F**AC AAA GGT GCA	6748.4322	6748.1707
	ON^{ATTO}_{int}	ATTO Rho11		6857.4488	6857.3838
	ON^{Cy3}_{term}	Cy3	ACG TCG ATA AAC AAA GGT GCA **F**	7061.4914	7060.2757
	ON^{ATTO}_{term}	ATTO Rho11		7170.5078	7169.4843

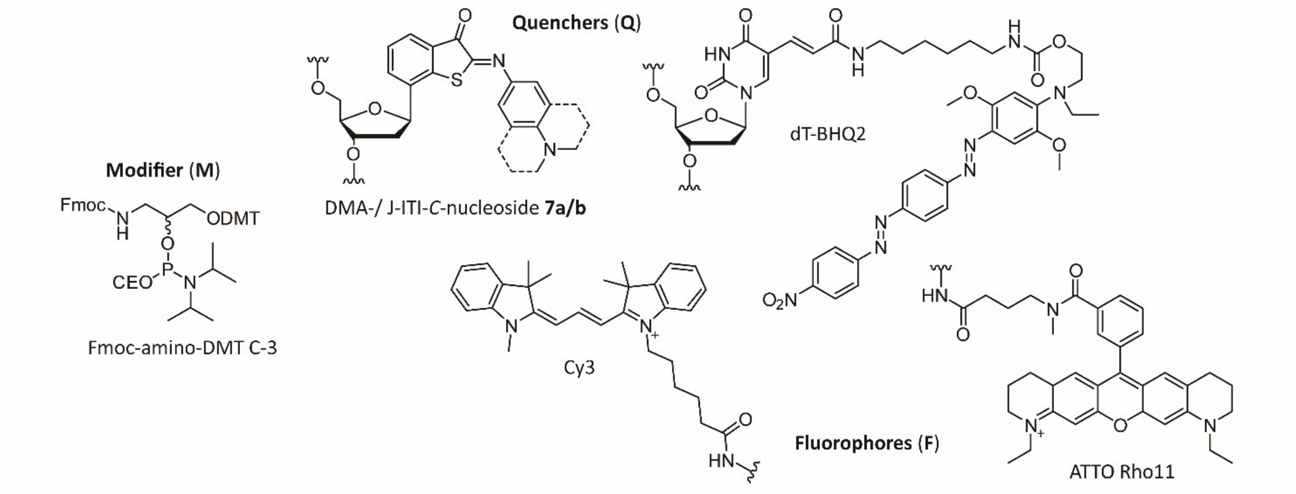

Fig. S6 Chemical structures of the Fmoc-amino-DMT C-3 for subsequent NHS-labelling – Modifier (**M**), quencher – Quenchers (**Q**) and fluorophore – Fluorophores (**F**) moieties incorporated for oligonucleotide modification.

Incubation with 30% aqueous NH_3 solution (*Merck*) (2 hours, rt) was used for deprotection of all oligonucleotides with exception of the amino-modified constructs. Their cleavage was performed by incubation with 30% aqueous NH_3 solution (5 min, 130 °C MW) using an *Initiator EXP EU* microwave system (*Biotage*). The crude samples were concentrated under reduced pressure via vacuum centrifuge before purification. $\mathbf{ON}^{\mathbf{J}}_{int}$ was synthesized in DMT-off mode and directly purified. DMT-on synthesized oligonucleotides were purified using RP-HPLC chromatography, followed by the removal of the DMT protecting group with 80% AcOH (20 min, rt). The AcOH was removed under reduced pressure and the deprotected samples underwent a second RP-HPLC purification step. The purified oligonucleotides were desalted by spinfiltration with *Microsep Advance 1K Omega (Pall Corporation)* using RNase-free water followed by a final concentration via vacuum centrifuge.

5.2. Purification

RP-HPLC

Oligonucleotide purification and analysis via RP-HPLC were conducted using either an *Agilent 1200* system or *Agilent 1260 Infinity* system equipped with *Waters XBridge* columns (as specified in Table S4). 400 mM hexafluoroisopropanol (HFIP) and 16.3 mM triethylamine (Et_3N) at pH 7.8 against a gradient of MeOH was employed. Oligonucleotides were eluted using various methanol gradients (detailed in Table S5). Unless otherwise specified, purification and analysis were carried out at 60 °C.

Table S4 Columns for RP-HPLC purification.

Column	Gradient
1	XBridge Peptide BEH C18 OBD Prep Column, 300 Å, 5 µm, 10 x 250 mm
2	XBridge BEH C18 OBD Prep Column, 130 Å, 5 µm, 10 x 50 mm
3	XBridge Peptide BEH C18 Column, 300 Å, 3.5 µm, 4.6 x 250 mm

Table S5 Gradients for RP-HPLC purification of the synthesized oligonucleotides.

	Column	Gradient	Flow rate
DMT-on	1	0-2 min 5% MeOH, 2-26 min 5-60% MeOH	3.5 mL m^{-1}
DMT-off	2	0-2 min 5% MeOH, 2-8 min 5-50% MeOH	3.5 mL m^{-1}
analytical	3	0-2 min 5% MeOH, 2-26 min 5-60% MeOH	0.7 mL m^{-1}

Mass Spectrometry

The purity and identity of all oligonucleotide strands were verified via LC-MS on an *Agilent 1200* system equipped with a *Waters XBridge Peptide BEH C18* column (300 Å, 3.5 µm, 2.1 mm x 250 mm), along with a *Bruker micrOTOF-QII* ESI. The LC-MS analysis utilized the same buffer system used for purification and analytics, conducted at 40 °C.

5.3. Hypothesis – Structural Orientation of the ITI-Chromophore within the DNA Duplex

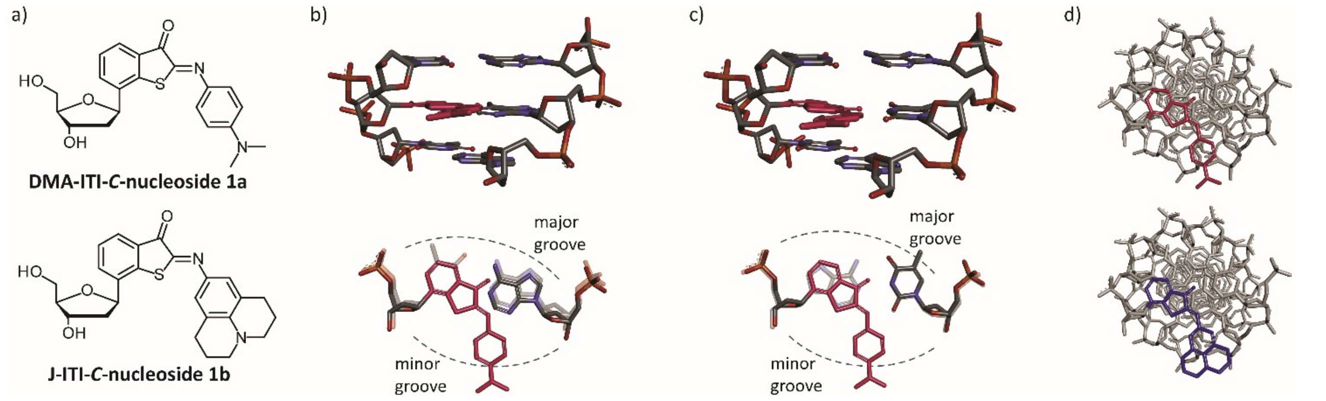

Fig. S7 (a) Schematic Illustration: The iminothioindoxyl-(ITI)-derived quencher nucleosides **1a** and **1b** introduced in this study (BIOVIA Discovery Studio). (b) and (c) Side and top views of a dsDNA structure in which one nucleoside has been replaced by nucleoside 1b opposite a dA or dT residue, respectively. The (overlapping) progenitor dT and dA residues, respectively, where nucleoside **1a** has been incorporated, are shown with faded colors. (d) Positioning of the ITI chromophores of compounds **1a** and **1b** in the native duplex structure viewed along the helix axis.

6. Fluorophore NHS-Labeling

NHS-Labeling

Internal and terminal introduction of the fluorophore modification of the NH_2-modified oligonucleotides $\mathbf{ON}^{\mathbf{NH2}}_{\boldsymbol{int}}$ and $\mathbf{ON}^{\mathbf{NH2}}_{\boldsymbol{term}}$ were performed via NHS-labeling of the respective fluorophores Cy3 (*Lumiprobe GmbH*) or ATTO Rho11 (*ATTO-TEC GmbH*) (Fig. S6, Section 5.1). The modifications were implemented at 5 nmol scales. The dry oligonucleotide aliquots were solved in 150 µL freshly prepared borate-buffer (0.1 M $Na_2B_4O_7$, pH 8.41). The fluorophore stock solutions were freshly prepared in DMF and 50 µL (100 nmol) were directly added into the oligonucleotide solution. After homogenization, the reaction mixture was shaken at 500 U min^{-1} (6 h, 37 °C).

Purification

The reaction was stopped by removal of the fluorophore excess using *illustra NAP columns (GE Healthcare)* and RNase-free water. The fractions received were checked for oligonucleotide absorbance (260 nm) as well as fluorophore specific absorbance using a *NanoDrop2000 (Thermo Fisher)* and combined accordingly. The solvent was removed by vacuum centrifuge and immediately purified by RP-HPLC chromatography (Agilent, column 3, gradient 3 60 °C, 0.7 mL min^{-1}). The samples were dried again and the modified oligonucleotide was desalted by spinfiltration with *Microsep Advance 1K Omega (Pall Corporation)* using RNase-free water followed by a final concentration via vacuum centrifuge. Table S3 (Section 5.1) shows the corresponding fluorophore modified oligonucleotide sequences.

7. Fluorescence Studies

7.1. Materials and Methods

For the evaluation of the fluorescence quenching efficiencies of our new quencher molecules **1a/b** internally and terminally within oligonucleotides, fluorescence studies were performed using a *Tecan Infinite M200 Pro Plate* equipped with black flat bottom polystyrene 96-well microplates (*Greiner Bio-One*). As fluorophores, commercially available Cy3 (Abs. 554 nm, Em. 568 nm) and ATTO Rho11 (Abs. 572 nm, Em. 595 nm) were purchased from *Lumiprobe* or *ATTO-TEC*. Based on the absorption and emission properties Cy3 was paired with DMA-ITI **1a** and ATTO Rho11 was paired with J-ITI **1b**. For comparison, dT-BHQ2 was used as a commercially available reference quencher for both fluorophores. Cy3 was excited at 530 nm (detection range: 550-850 nm), while ATTO Rho11 was excited at 550 nm (detection range: 580-850 nm). All measurements were conducted at 25 °C.

For background correction the pure solvent was measured. To determine the maximum fluorescence (reference), the fluorophore-modified strand of each system was measured without any quencher strand present. Within the fluorescence study, different fluorophore/quencher ratios (100:1, 10:1, 7:1, 3:1, 1:1, 1:3, 1:7, 1:10) were characterized keeping the fluorophore concentration constant. All samples were prepared and characterized as triplicates (n = 3) with respective sample volumes of 100 µL 1x PBS buffer solution (pH 7.4). Hybridization of the modified duplex structures was performed by incubation at 95 °C for 3 minutes, followed by slow passive cooling to ambient temperature.

The data-sets of all recorded spectra were background corrected by subtracting the emission of the pure solvent. The measured triplicates were processed to obtain the average value and standard deviation. For evaluation of the residual fluorescence of each system characterized, the data for the different fluorophore/ quencher ratios were normalized to the maximum fluorescence (reference) of the respective system. The reference was set to a value of 1, representing 100% of the possible fluorescence emission. The normalized residual fluorescence was plotted against the various fluorophore/ quencher ratios for each system (Fig. 4). The primary data measured is shown in Fig. S8.

7.2. Results

An additional list of the results of the fluorescence reduction can be found in Table S6. For this purpose, the quotient of the residual fluorescence and the maximum reference fluorescence was formed. The values are given in percent.

Table S6 Fluorescence studies to evaluate internal and terminal quencher efficiencies. Combinations of Cy3 / DMA-ITI **1a** and ATTO Rho11 / J-ITI **1b** were chosen as F/Q pairs with dT-BHQ2 as quencher reference for both fluorophores.

Fluorescence strand	Quencher strand	Residual fluorescence F/Q ratio 1:1	Residual fluorescence F/Q ratio 1:10
ON^{Cy3}_{int}	ON^{DMA}_{int}	33%	13%
	ON^{BHQ2}_{int}	1%	1%
ON^{Cy3}_{term}	ON^{DMA}_{term}	21%	13%
	ON^{BHQ2}_{term}	3%	1%
ON^{ATTO}_{int}	ON^{J}_{int}	24%	8%
	ON^{BHQ2}_{int}	2%	1%
ON^{ATTO}_{term}	ON^{J}_{term}	12%	5%
	ON^{BHQ2}_{term}	7%	2%

7.3. Primary Data

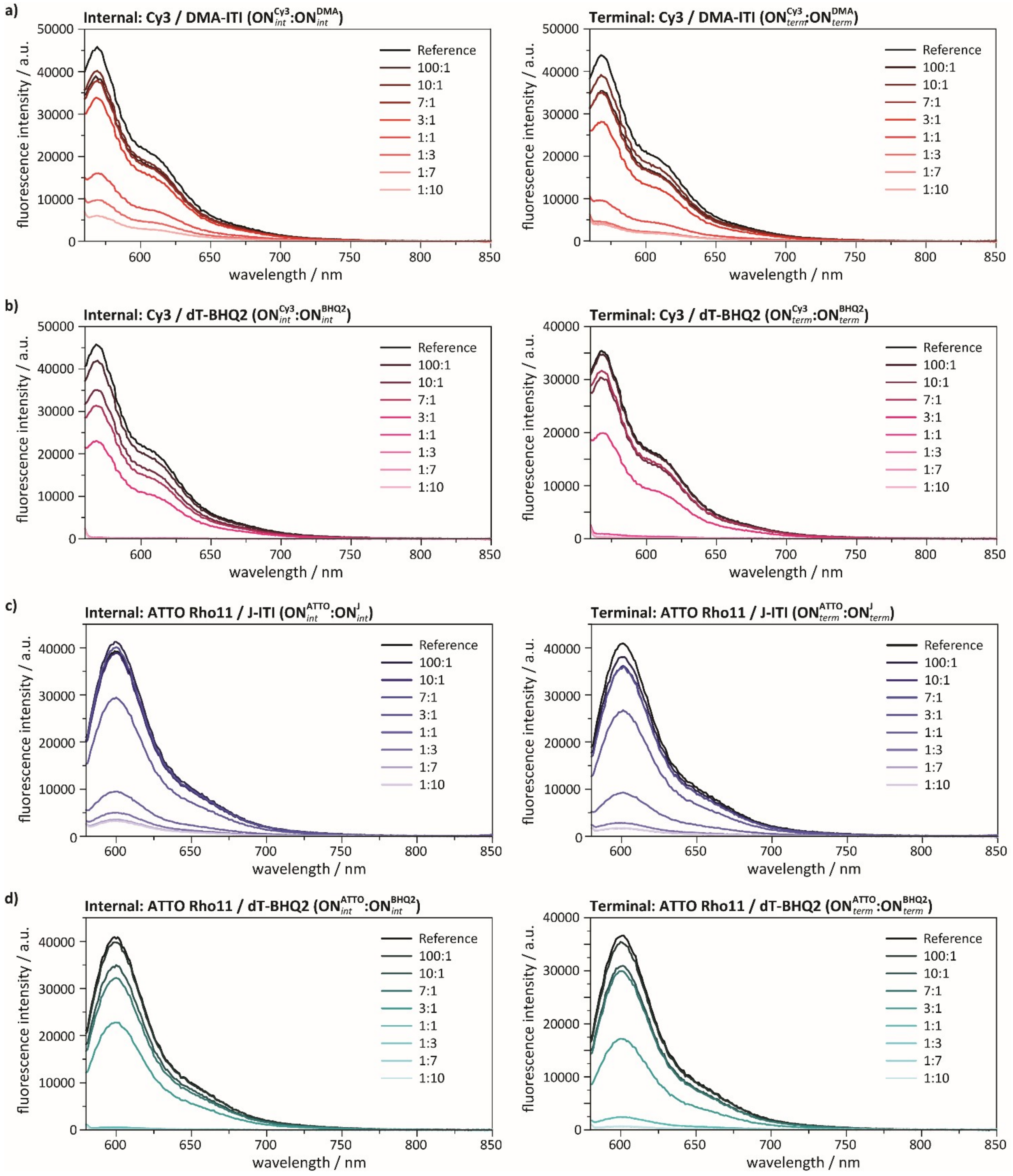

Fig. S8 Primary data of all fluorescence measurements performed within this ratio-dependent fluorescence study. Internal modifications are shown on the left, terminal in the right. (a) Cy3 / DMA-ITI, (b) Cy3 / dT-BHQ2, (c) ATTO Rho11 / J-ITI and (d) ATTO Rho11 / dT-BHQ2. The corresponding F-labelled oligonucleotide strand serves as the reference.

8. Melting Temperature Studies

8.1. Materials and Methods

The characterization of the duplex melting temperatures was performed on an *Evolution 300* UV-Vis spectrometer equipped with a peltier element (both *Thermo Fisher Scientific*). The measurements were performed in a quartz glass cuvette with a light path of 10.00 mm (*Hellma Analytics*). In preparation for the measurements, the solvent was premeasured for background correction. The samples were subjected to 4 temperature cycles ranging from 20°C to 80°C and back, with a gradual increase or decrease of 1°C per minute, while their absorbance was monitored at 260 nm.

For this purpose, all samples were prepared in 1 µM concentration in a total volume of 1 mL 1x PBS buffer (pH 7.4) containing 1 nmol of each oligonucleotide strand. As references, both native duplexes **WT1^{dT}:WT2^{dA}** and **WT1^{dA}:WT2^{dT}** were analyzed. Followed by the characterization of the strand including an internal ON^{DMA}_{int} / ON^{J}_{int} / ON^{BHQ2}_{int} or terminal quencher modification ON^{DMA}_{term} / ON^{J}_{term} / ON^{BHQ2}_{term} with the complementary strands **WT2^{dA}** or **WT2^{dT}**. This enables the evaluation of the induced disruption of the native structure dependent on the presence of a purine or pyrimidine as the adjacent nucleobase. The arithmetic mean of the inflection points of seven obtained curves was used to determine the duplex melting temperature including the corresponding error.

Followed by the characterization of the oligonucleotide strands including a terminal quencher modification ON^{DMA}_{term} / ON^{J}_{term} / ON^{BHQ2}_{term} with the complementary strand **WT2^{dA}**. The strands with a terminal quencher modification ON^{DMA}_{int} / ON^{J}_{int} / ON^{BHQ2}_{int} were analyzed with the complementary strands **WT2^{dA}** or **WT2^{dT}**. This enables the evaluation of the induced disruption of the native structure dependent on the presence of a purine or pyrimidine as the adjacent nucleobase, which was expected to be more significant with the internal modification.

8.2. Results

The results of the duplex melting temperature study are displayed in Fig. S9 and Fig. S10. For better visualization to compare the subtle differences in the melting temperature, the plots were smoothed. The original primary data is given in the following Section of the Supporting Information (Fig. S11 and Fig. S12, Section 8.3). The resulting values including the corresponding standard deviation are listed in Table S7.

Table S7 Melting temperature of the duplex structure **WT1^{dT}:WT2^{dA}** or **WT1^{dA}:WT2^{dT}** (native) as well as the quencher-modified strands ON^{DMA}_{int}, ON^{J}_{int} and $ON^{dT-BHQ2}_{int}$ (internal) and ON^{DMA}_{term}, ON^{J}_{term} and $ON^{dT-BHQ2}_{term}$ (terminal) with the native AS strand **WT2^{dA}** (left) or **WT2^{dT}** (right). All values are given with the corresponding standard deviation.

antisense strand: **WT2^{dA}**			antisense strand: **WT2^{dT}**		
	**Tm ± standard deviation / °C**	**ΔTm / °C**		**Tm ± standard deviation / °C**	**ΔTm / °C**
WT1^{dT}	70.3 ± 0.4	-	WT1^{dA}	67.7 ± 0.6	-
ON^{DMA}_{term}	70.5 ± 0.5	+ 0.2			
ON^{J}_{term}	71.1 ± 0.3	+ 0.8			
ON^{BHQ2}_{term}	68.7 ± 0.7	- 1.6			
ON^{DMA}_{int}	63.3 ± 0.5	- 7.0	ON^{DMA}_{int}	65.7 ± 0.2	- 2.0
ON^{J}_{int}	65.8 ± 0.6	- 4.5	ON^{J}_{int}	66.4 ± 0.3	- 1.3
ON^{BHQ2}_{int}	66.2 ± 0.4	- 4.1	ON^{BHQ2}_{int}	66.5 ± 0.4	- 1.2

To evaluate the disruption of native duplex structure due to terminal or internal quencher modification, melting temperature measurements were performed. Fig. S9 indicates that terminal quencher modifications **1a/b** with **$WT2^{dA}$** as the complementary strand do not influence the duplex stability based on the absence of a melting temperature decrease. For the reference system – dT-BHQ2 a very small destabilization was observed.

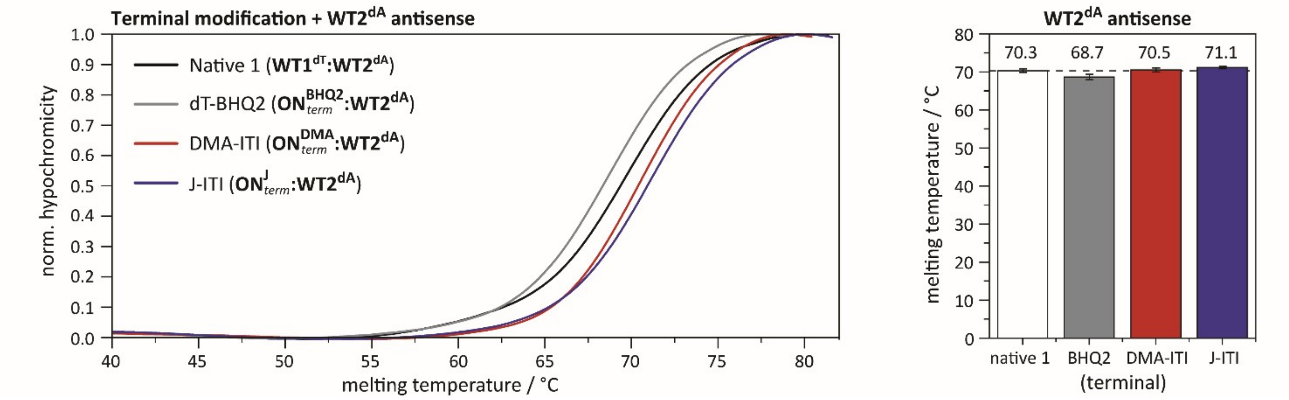

Fig. S9 Melting temperatures curves of the terminal modifications with **$WT2^{dA}$** as the antisense strand. Left: plot of the normalized hypochromicity against the melting temperature, right: melting temperature values including their standard deviation displayed as a bar chart.

The results for internal quencher modifications are shown in Fig. S10a shows melting temperatures with the antisense strand WT2dA featuring the structurally large purine dA as the adjacent nucleobase. Small destabilizations in the range of 4-7 °C (6-10%) were observed for both the new quencher molecules **1a/b** as well as dT-BHQ2. To fully evaluate the steric effects of the introduced modifications, a smaller pyrimidine dT was also used as the opposing nucleobase (Fig. S10b). Here, the perturbations were reduced to remarkable 1.3-2 °C (2-3%), thus showing a dependence of the degree of perturbation on the environment. In summary, we were able to show that the native duplex structure remains largely undisturbed when modified with **1a/b**.

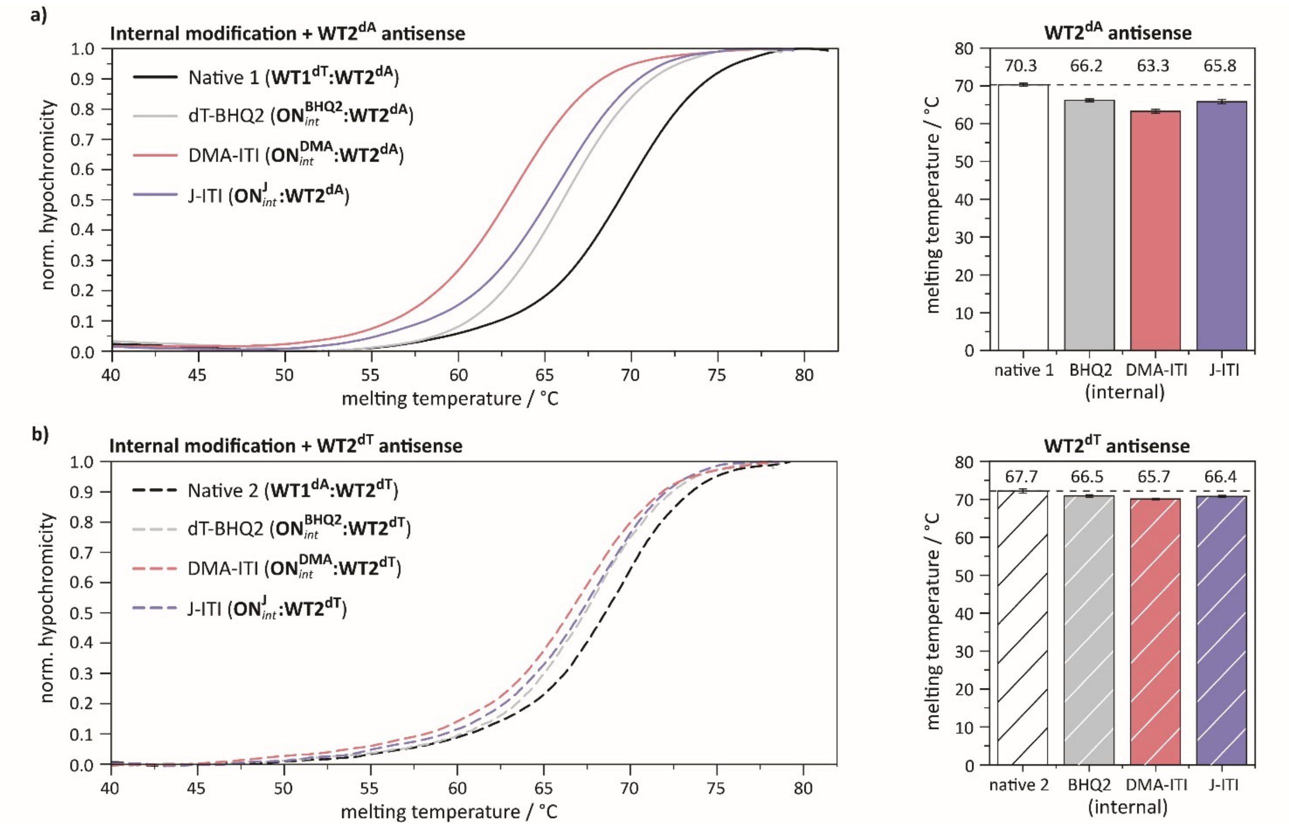

Fig. S10 Melting temperatures curves of the internal modifications with (a) **$WT2^{dA}$** and (b) **$WT2^{dT}$** as the antisense strand. Left: plot of the normalized hypochromicity against the melting temperature, right: melting temperature values including their standard deviation displayed as a bar chart.

8.3. Primary Data

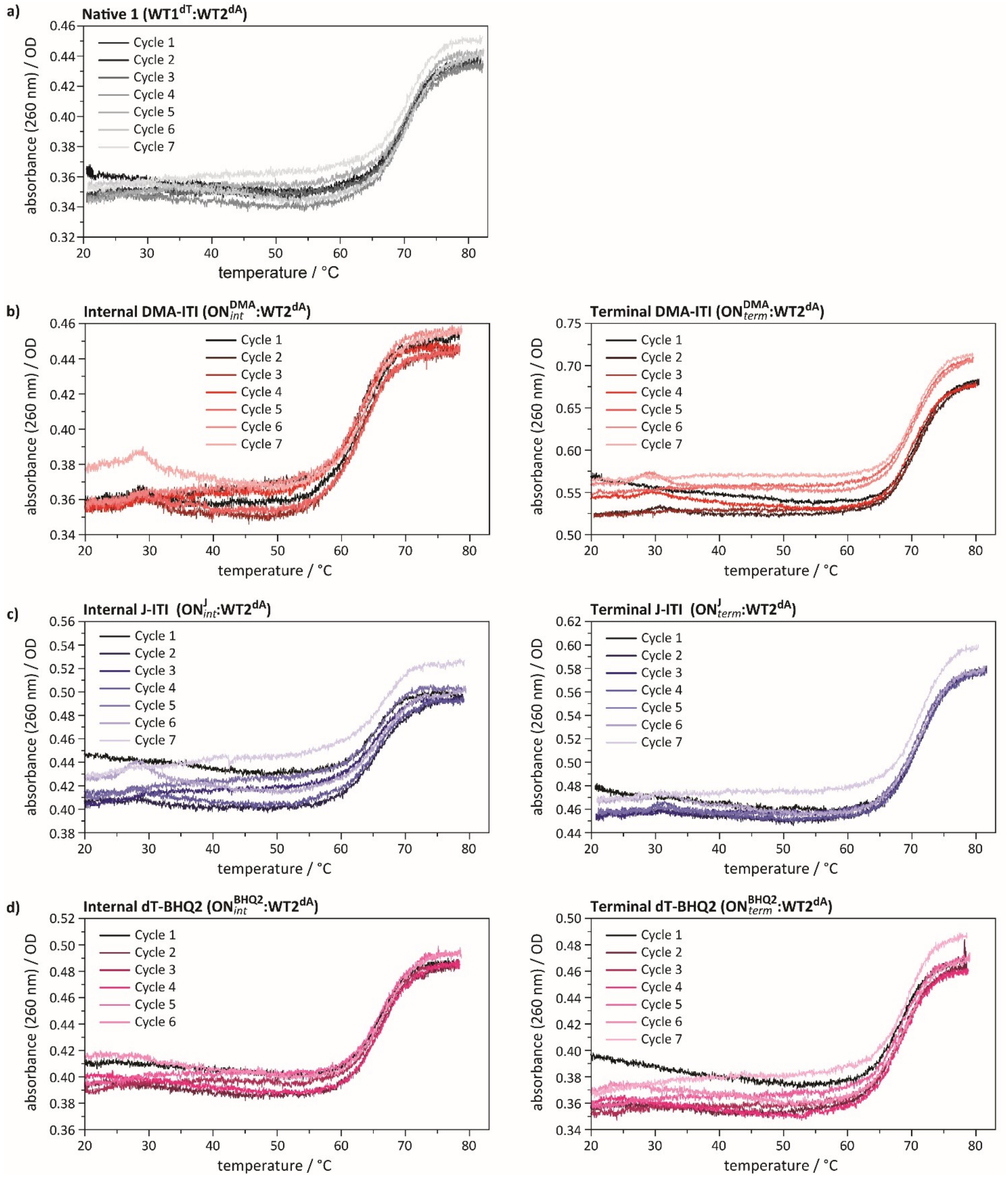

Fig. S11 Primary data of the melting temperature curves with **WT2^{dA}** as the antisense strand. Internal modifications are shown on the left, terminal in the right. (a) **WT1^{dT}** – Native oligonucleotide duplex (reference, grey), (b) **ON^{DMA}** – DMA-ITI (red), (c) **ON^{J}** – J-ITI (blue) and (d) **ON^{BHQ2}** – dT-BHQ2 (pink).

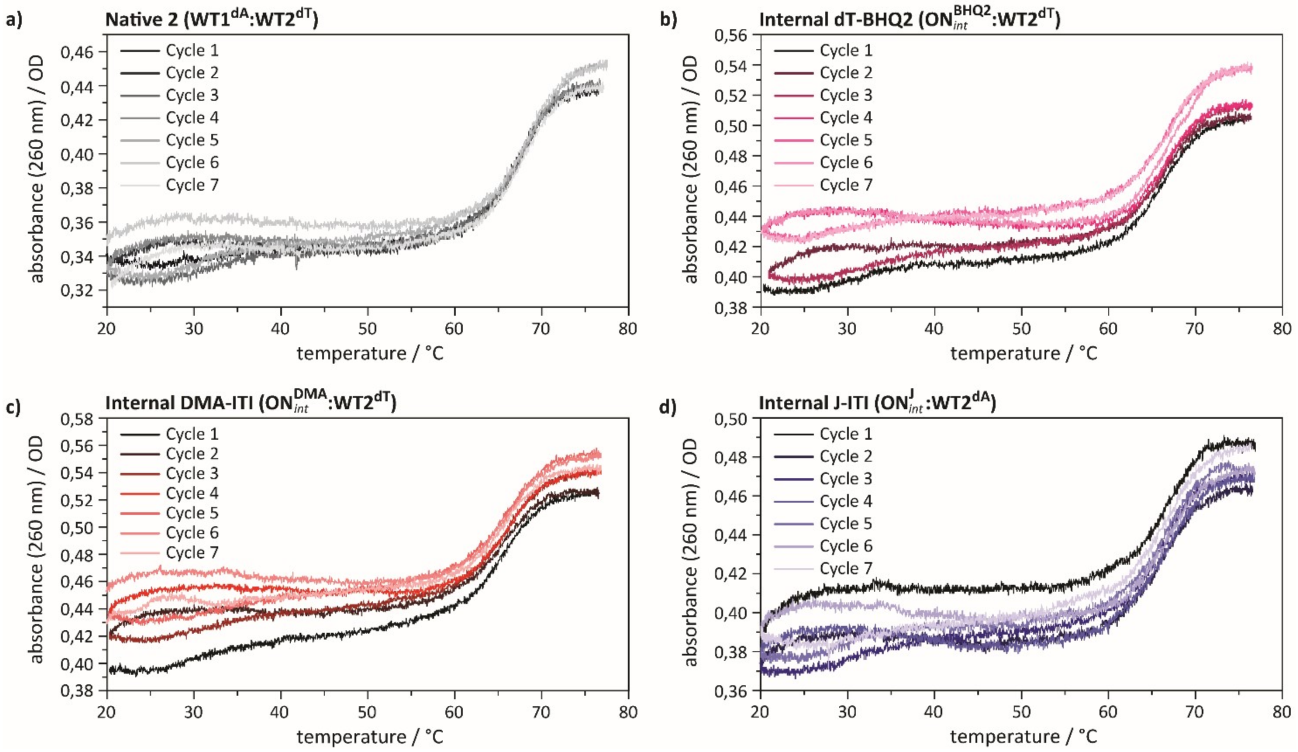

Fig. S12 Primary data of the melting temperature curves for internal modifications with **WT2^{dT}** as the antisense strand. (a) **WT1^{dA}** – Native oligonucleotide duplex (reference, grey), (b) **ON^{BHQ2}** – dT-BHQ2 (pink), (c) **ON^{DMA}** – DMA-ITI (red) and (d) **ON^{J}** – J-ITI (blue).

9. CD Spectroscopy

9.1. Materials and Methods

The CD spectroscopy was performed on a *Jasco J-715* CD spectrometer using quartz glass cuvettes with a light path of 1.00 mm (*Hellma Analytics*). The samples were characterized at 25 and 37 °C in the range of 200-340 nm. Each measurement was realized doing 5 repetitive scans that were averaged to obtain the respective trace. The parameters were set to a scanning speed of 200 nm/ min with a 1 s time constant, 1.0 nm data pitch and 1.0 nm bandwidth.

All samples were prepared in 10 µM concentration in a total volume of 100 µL 1x PBS buffer (pH 7.4) containing 1 nmol of each oligonucleotide strand. As reference, the native duplex **WT1^{dT}:WT2^{dA}** was analyzed first. Followed by the characterization of the strand including an internal $\mathbf{ON}^{\mathbf{DMA}}_{int}$ / $\mathbf{ON}^{\mathbf{J}}_{int}$ / $\mathbf{ON}^{\mathbf{BHQ2}}_{int}$ or terminal quencher modification $\mathbf{ON}^{\mathbf{DMA}}_{term}$ / $\mathbf{ON}^{\mathbf{J}}_{term}$ / $\mathbf{ON}^{\mathbf{BHQ2}}_{term}$ with the complementary strand **WT2^{dA}**. Hybridization of the modified duplex structures was performed by incubation at 95 °C for 3 minutes, followed by slow passive cooling to ambient temperature.

All recorded CD spectra were baseline corrected by subtracting the background signal of the solvent and smoothed before plotting.

9.2. Results

To verify the results of the melting temperature measurements to characterize the disruptions of the native duplex structure induced by quencher modification, additional CD spectroscopy was conducted. Terminal and internal modifications for the chromophore systems DMA-/ and J-ITI, as well as the commercially available reference system dT-BHQ2 were analyzed in comparison to the unmodified native duplex structure. The data (Fig. S13) indicate that all modified constructs, regardless of the temperature at which the experiments were conducted, exhibit traces consistent with CD spectra of native B-DNA helices. Generally, a negative band at approximately 245 nm and a positive band at around 275 nm are observed. Both bands exhibit similar intensities, and the integral of the spectral curve is close to zero. Upon closer examination, minimal deviations from the native structure are evident for all constructs, consistent with the supporting the results of the conducted melting temperature measurements. Leading to the assumption of minimal disturbance of the native oligonucleotide structure.

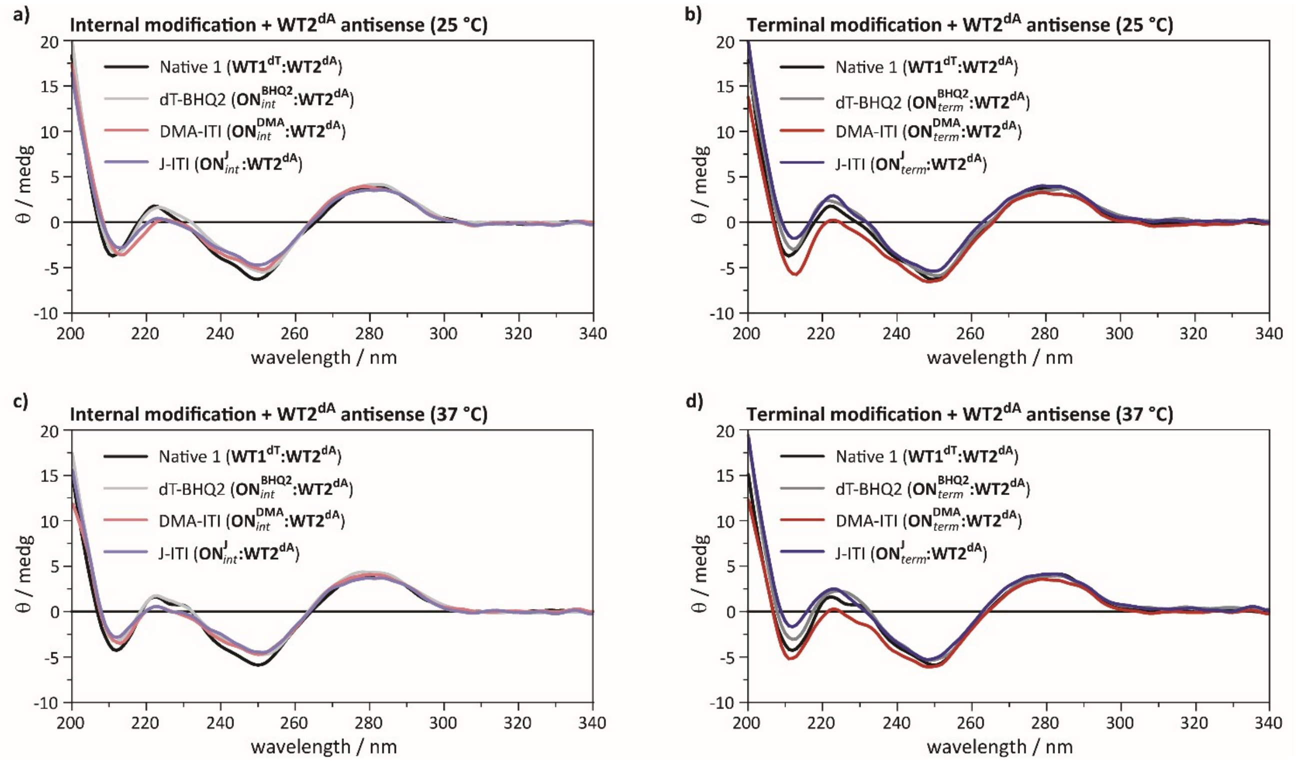

Fig. S13 CD spectroscopy of (a) internal modifications at 25 °C, (b) terminal modification at 25 °C, (c) internal modifications at 37 °C and (d) terminal modifications at 37 °C. All measurements were performed using **WT2^{dA}** as the antisense strand. Native 1 (black), dT-BHQ2 (grey), DMA-ITI (red) and J-ITI (blue).

10. MD Simulations

All-atom molecular dynamics (MD) simulations were performed using the GROMACS package[10,11] based on the AMBER99 force field[12] including the Barcelona Supercomputer Center (bsc1) corrections[13,14] for DNA. The force field parameters for the quencher are based on the Generalized AMBER Force Field (GAFF),[15] which contains all atom types relevant for the quencher. Partial charges were determined by Hartree-Fock calculations with the 6-31G* basis set using Restrained Electrostatic Potential (RESP) charges.

Based on this setup, single-trajectory simulations of 1 μs duration were carried out for each system. The simulation box contained about 62,000 atoms, including the modified DNA, about 20,000 water molecules and 40 sodium atoms as counter ions. The SPC water model was employed. Periodic boundary conditions were used. The simulations were carried out within the isothermal-isobaric (NPT) ensemble using the Berendsen thermostat, and the equilibration time was about 50 ps. All simulations were carried out at T=300K.

In order to quantify the stability of the stacking interaction between the quencher and the neighboring nucleotides, two parameters were monitored, shown in Figure 5 of the main text and in Fig. S14 below. First, the distance parameter d keeps track of the distance between the centers of mass of the proximal aromatic ring of the quencher and the adjacent nucleobases, see panel a). Second, the tilt angle α is the angle between the normal vectors of the adjacent quencher and nucleobase (shown in magenta and green, respectively, in panel a). While Figure 5 of the main text shows time traces of these two parameters for the interaction between the quencher and the upper nucleobase (in 5' direction), Fig. S14 illustrates an analogous analysis for the interaction between the quencher and the lower nucleobase (in 3' direction). In the latter case, it turns out that significantly stronger fluctuations are observed than in the case of the upper nucleobase. For reference, Fig. S14 d) shows the same quantities for two native nucleobases at a distance from the chromophore. As can be inferred from Fig. S14, panels b) and c), especially the tilt angle between the quencher and lower nucleobase undergoes large-amplitude fluctuations that exceed those observed for the native nucleobases (panel d) up to a factor of two. Conversely, the interaction between the quencher and the upper nucleobase, shown in Figure 5 of the main text, exhibits a level of fluctuations in good agreement with pairs of native nucleobases. This suggests that the proximal aromatic ring of the J-ITI-*C*-nucleoside **1b** is mainly stabilized by π–stacking interactions with the upper nucleobase. In contrast, the interaction with the lower nucleobase is affected by the position of the distal aromatic ring of the quencher that is rotated towards the minor groove.

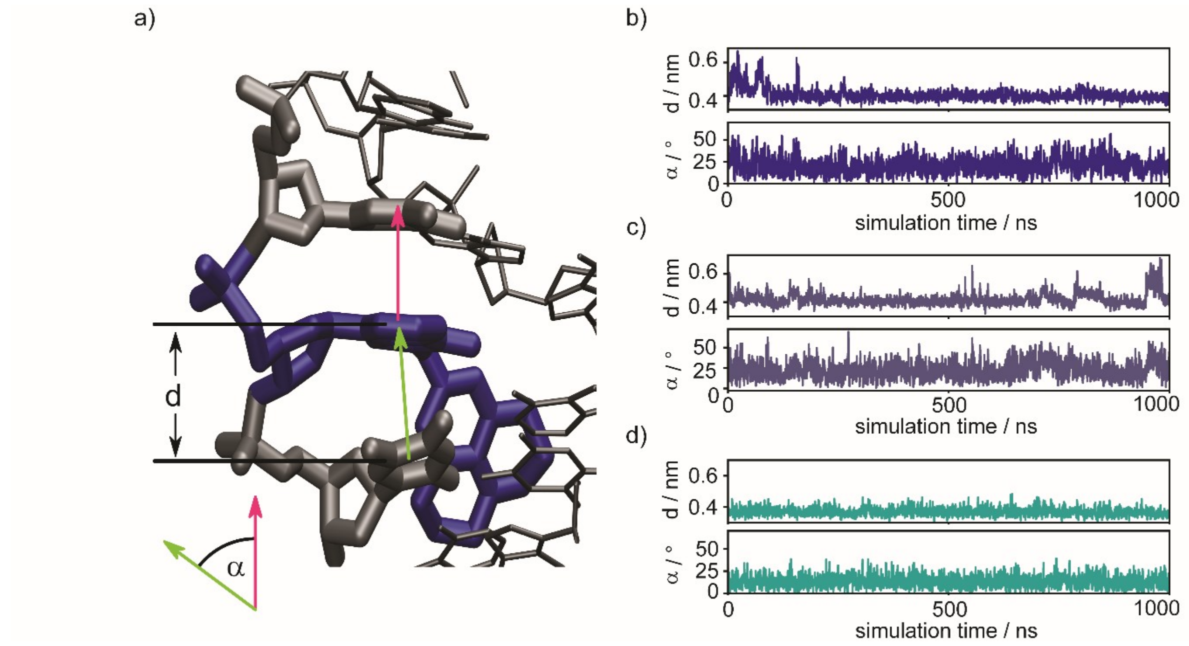

Fig. S14 Analogously to Figure 5 of the main text, a snapshot from an all atom MD simulation at 300K is shown, with the J-ITI-*C*-nucleoside **1b** highlighted in blue. The stacking distance parameter d and tilt angle α between normal vectors (marked in green and magenta) now relate to the interaction with the lower nucleobase, revealing less effective stacking as compared with the upper nucleobase. (b) Time traces for the parameters d and α for the **WT2**^{dA} modified duplex (see Fig. 3 of the main text). (c) Corresponding time traces of the parameters d and α for the **WT2**^{dT} duplex. (d) For reference, the distance parameter d and the tilt angle parameter α are shown for a pair of two nucleobases at a distance from the chromophore in the **WT2**^{dA} modified duplex. It is seen that the fluctuations are generally reduced as compared with panels (b) and (c) above. Conversely, Figure 5 of the main text, which focuses on the interaction between the quencher and the upper nucleobase, exhibits a similar level of fluctuations.

11. NMR Spectroscopy

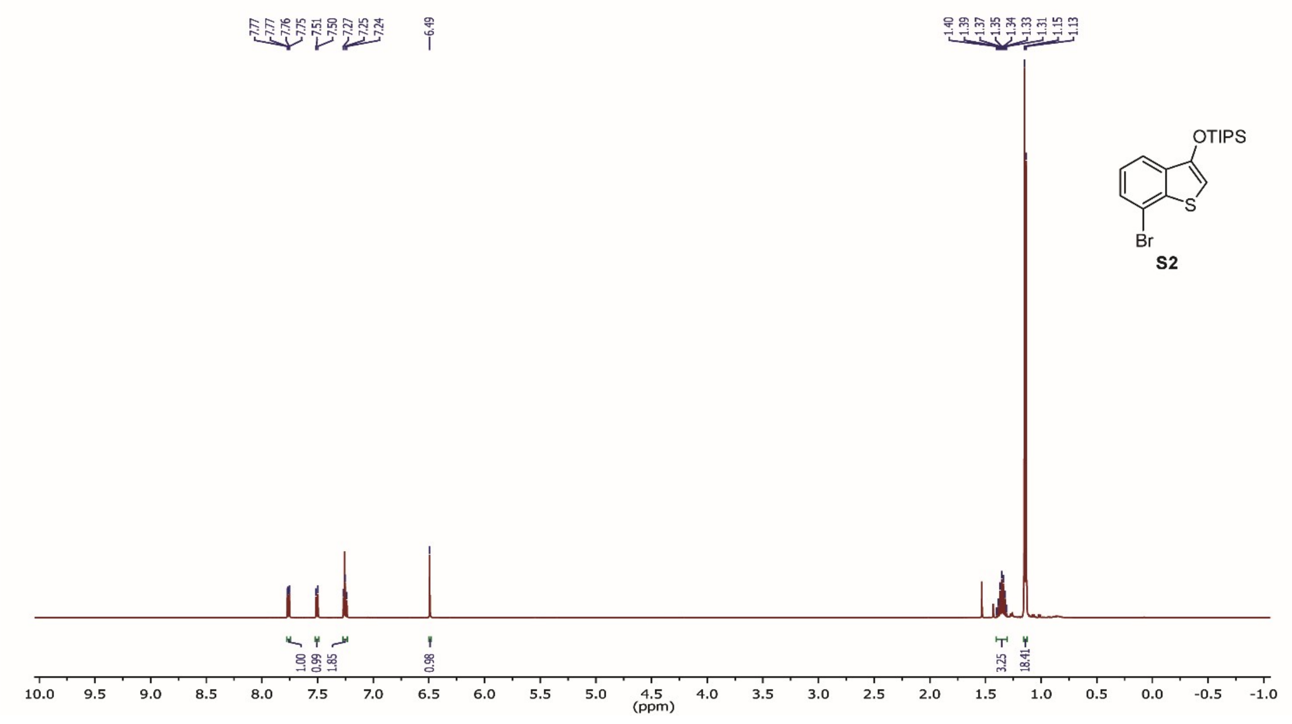

Fig. S15 ^{1}H-spectrum of compound **S2** in $CDCl_3$-*d*.

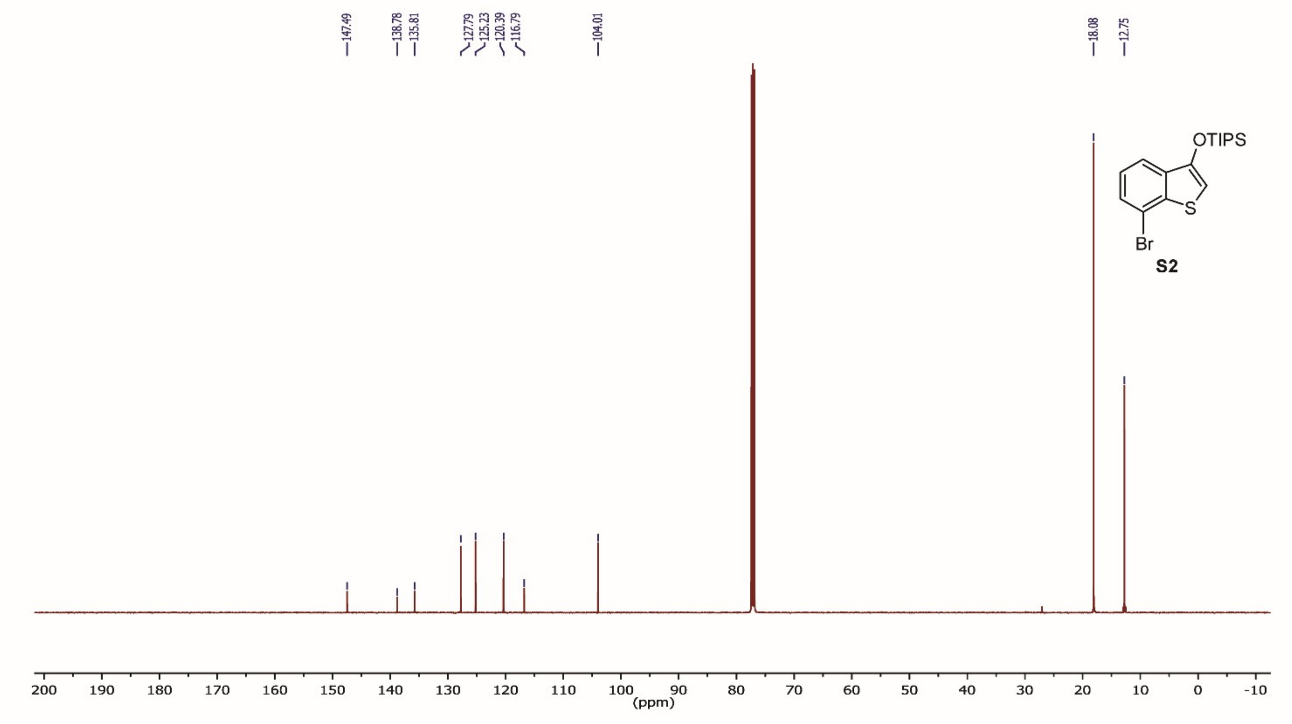

Fig. S16 $^{13}C\{^{1}H\}$-spectrum of compound **S2** in $CDCl_3$-*d*.

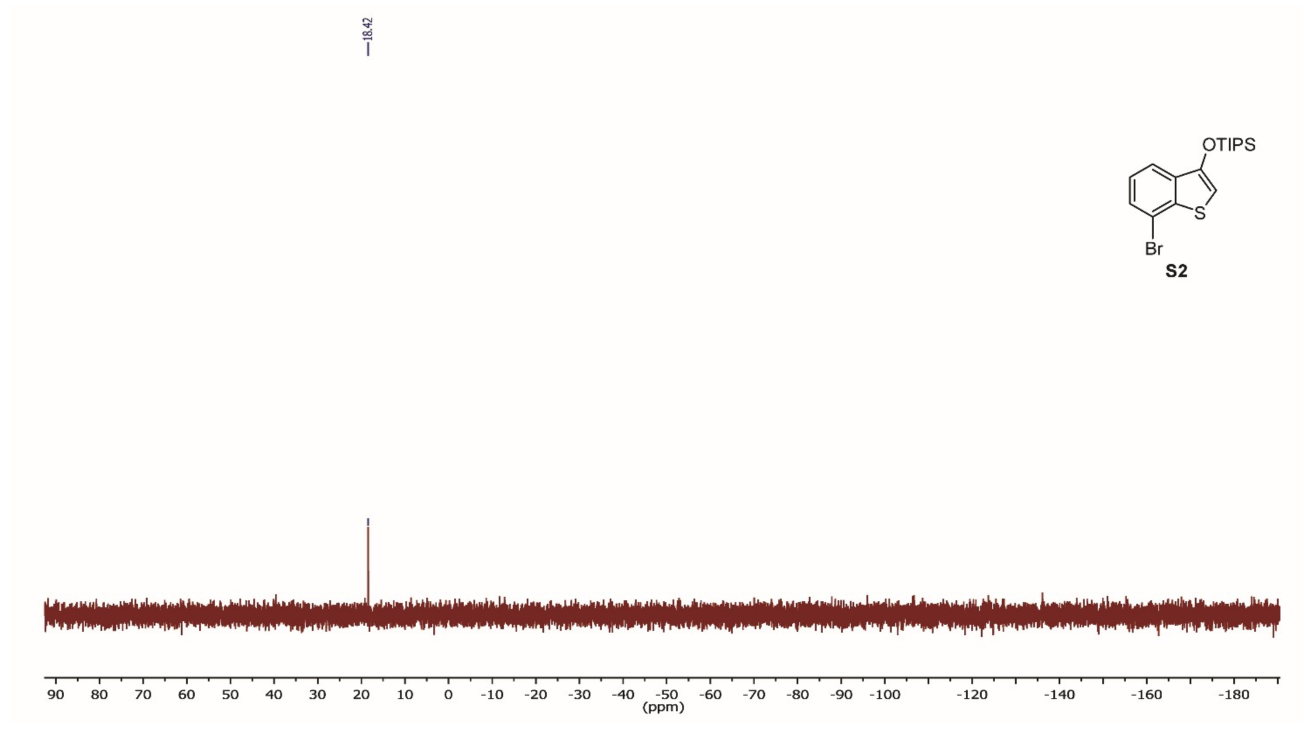

Fig. S17 ^{29}Si-INEPT-spectrum of compound **S2** in $CDCl_3$-*d*.

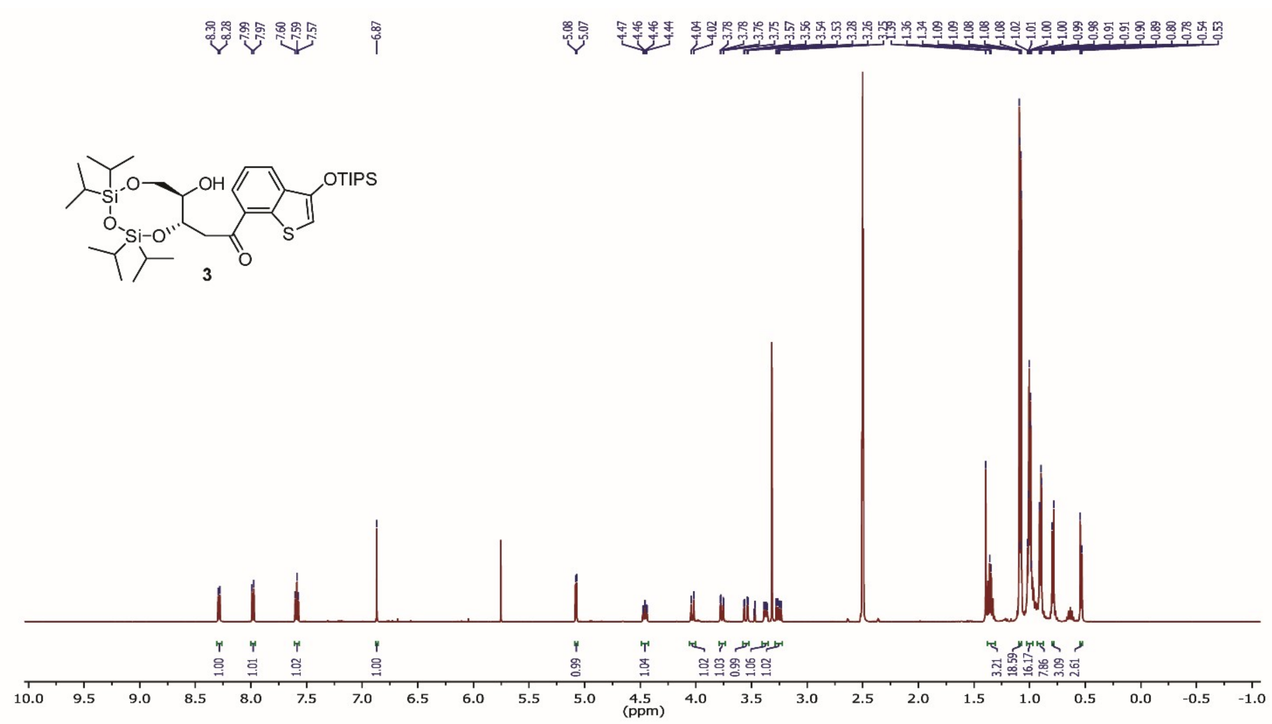

Fig. S18 ^{1}H-spectrum of compound **3** in DMSO-d_6.

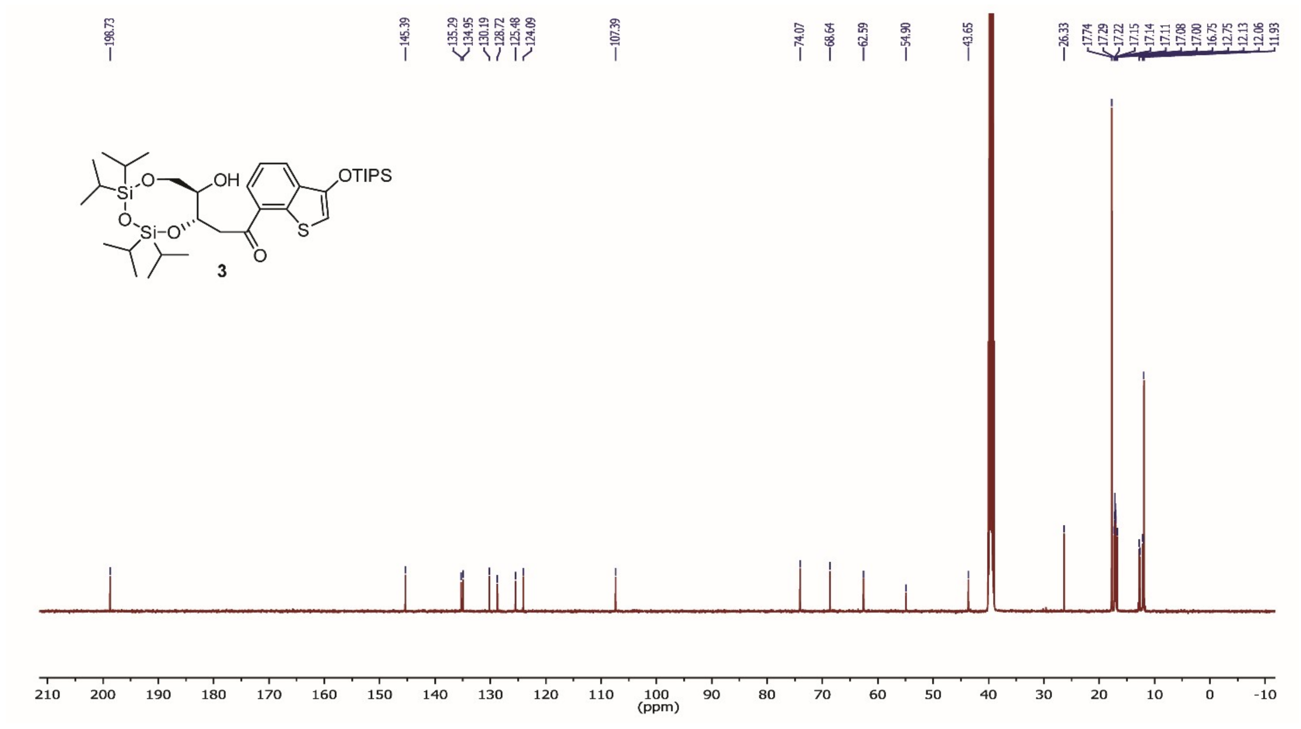

Fig. S19 $^{13}C\{^{1}H\}$-spectrum of compound **3** in DMSO-d_6.

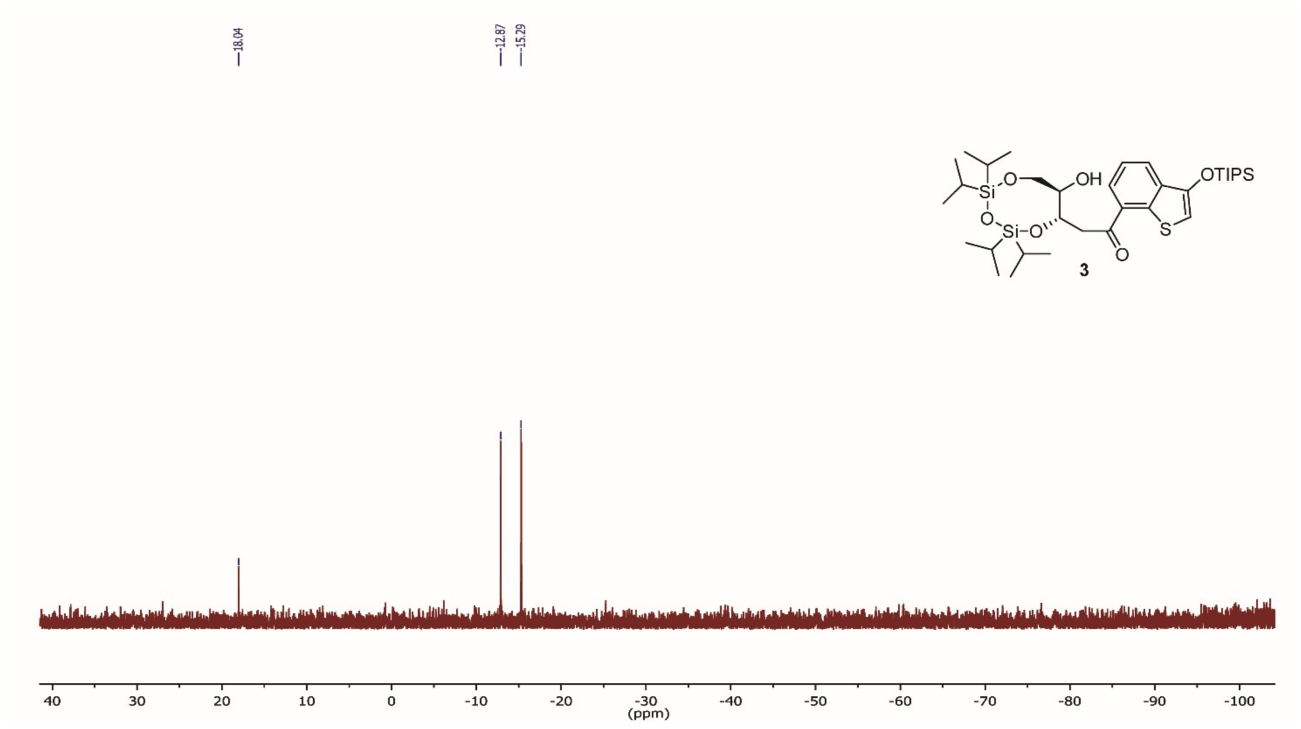

Fig. S20 $^{29}Si\{^{1}H\}$-spectrum of compound **3** in DMSO-d_6.

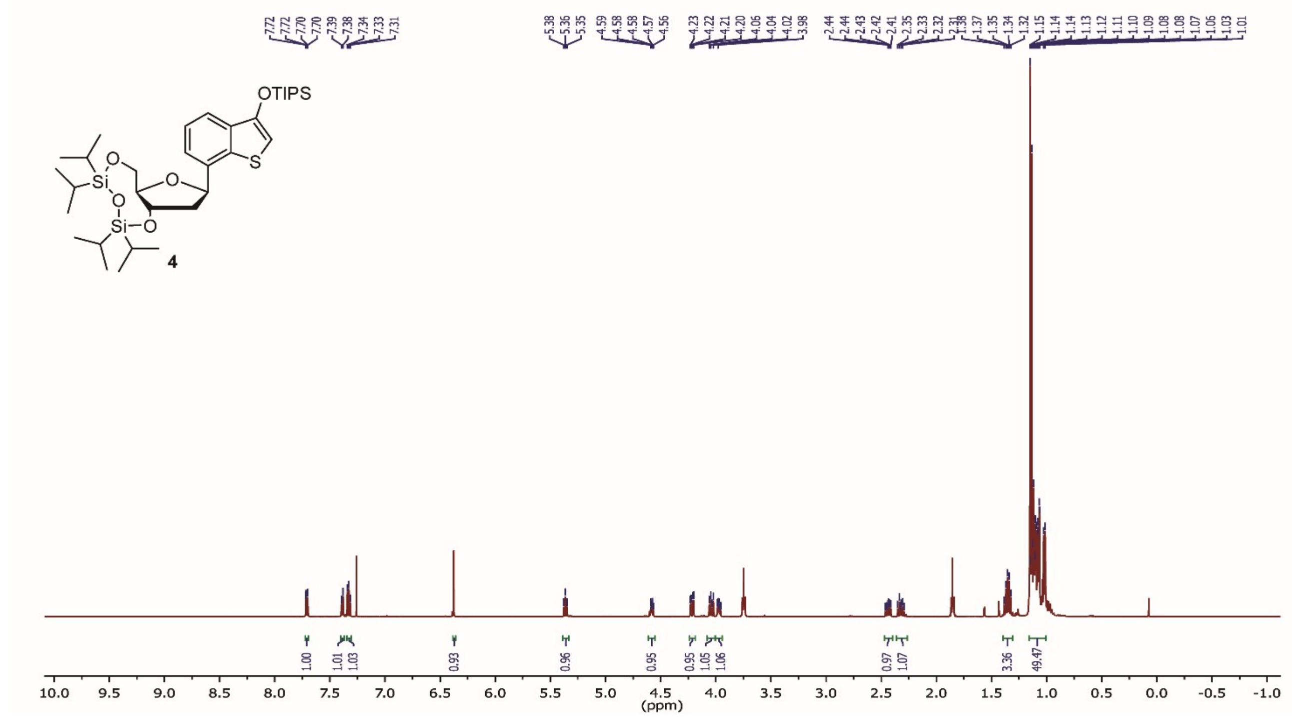

Fig. S21 ^{1}H-spectrum of compound **4** in $CDCl_3$-*d*.

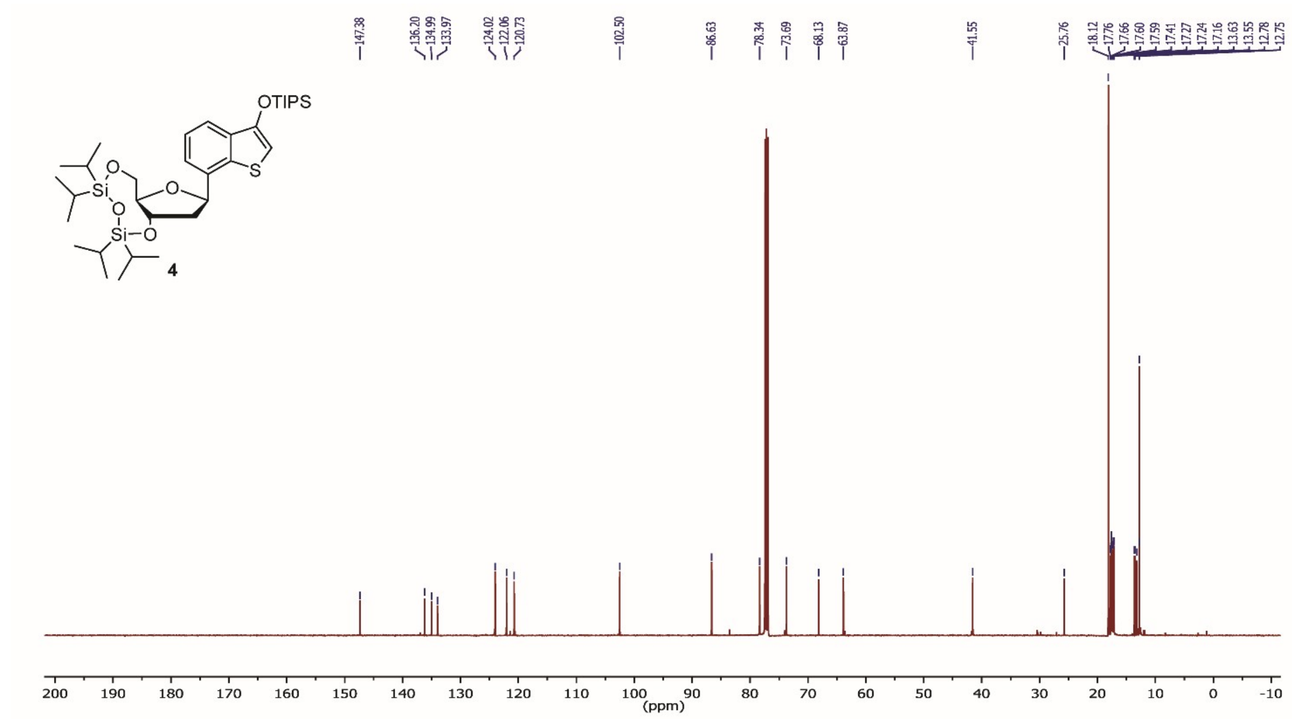

Fig. S 22 $^{13}C\{^{1}H\}$-spectrum of compound **4** in $CDCl_3$-*d*.

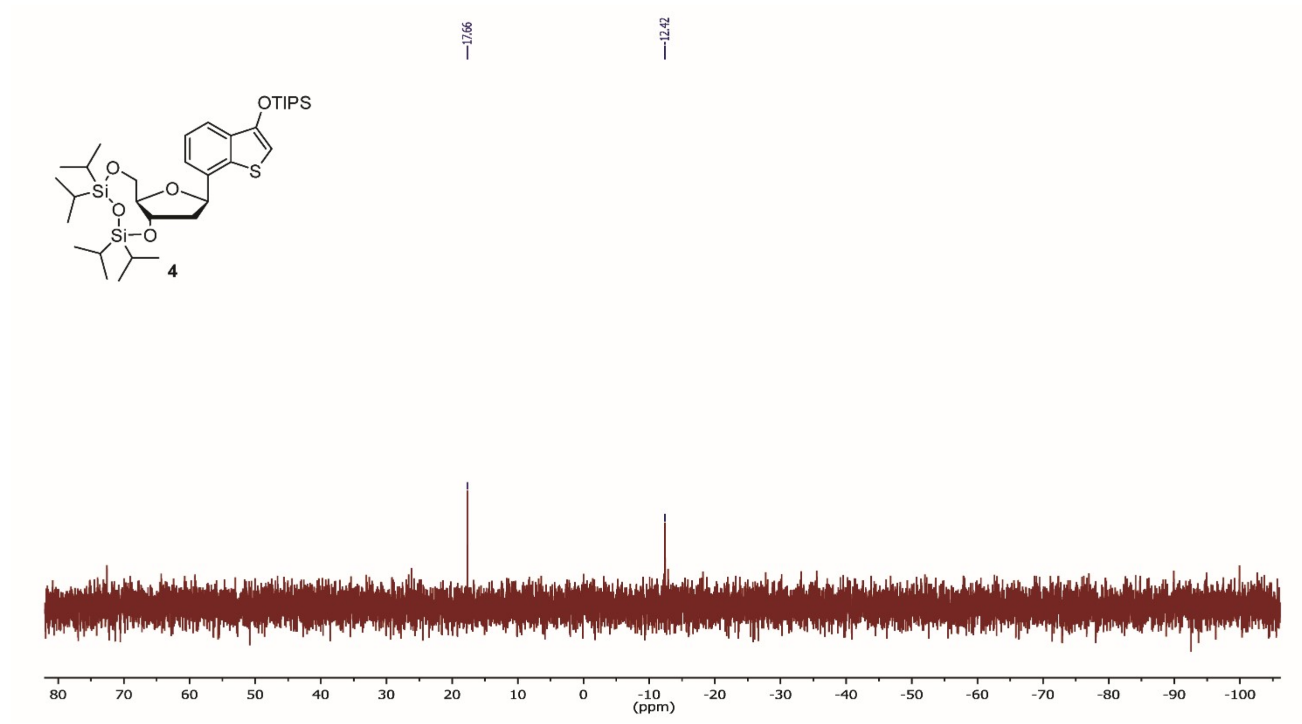

Fig. S23 ^{29}Si-INEPT-spectrum of compound **4** in $CDCl_3$-*d*.

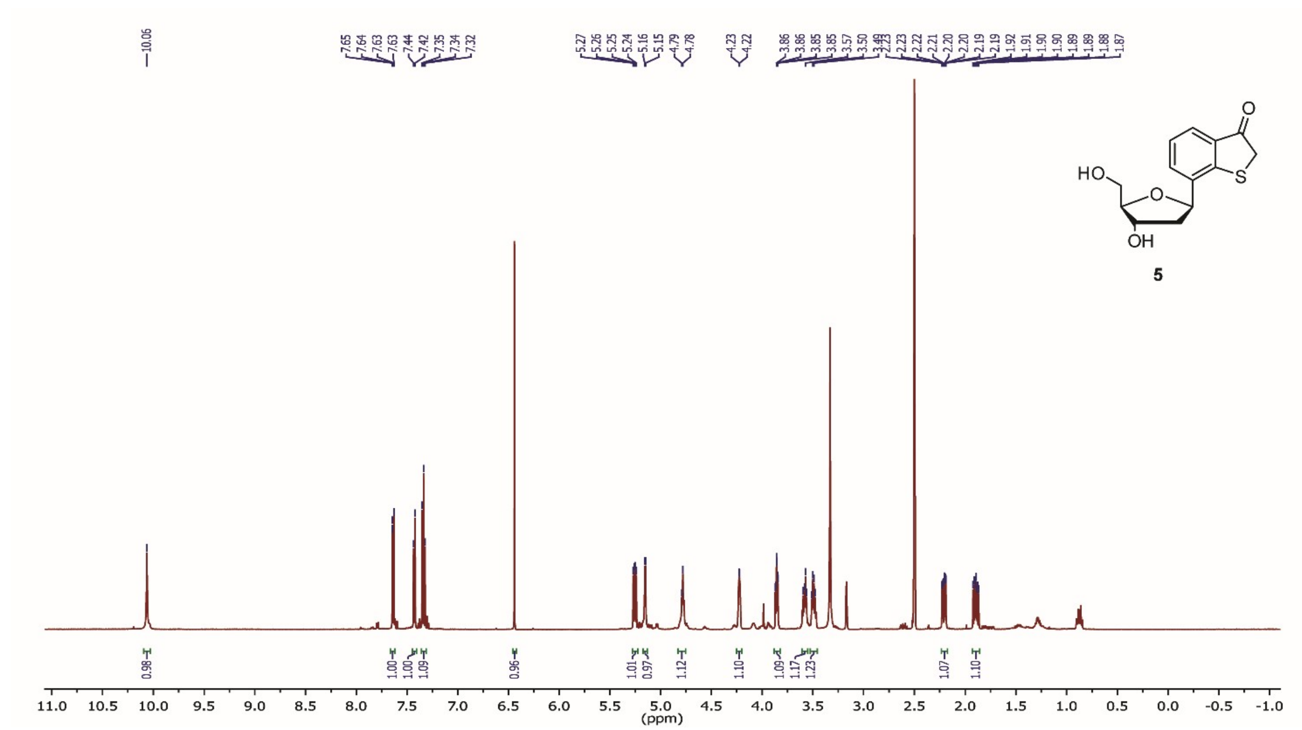

Fig. S24 ^{1}H-spectrum of compound **5** in DMSO-d_6.

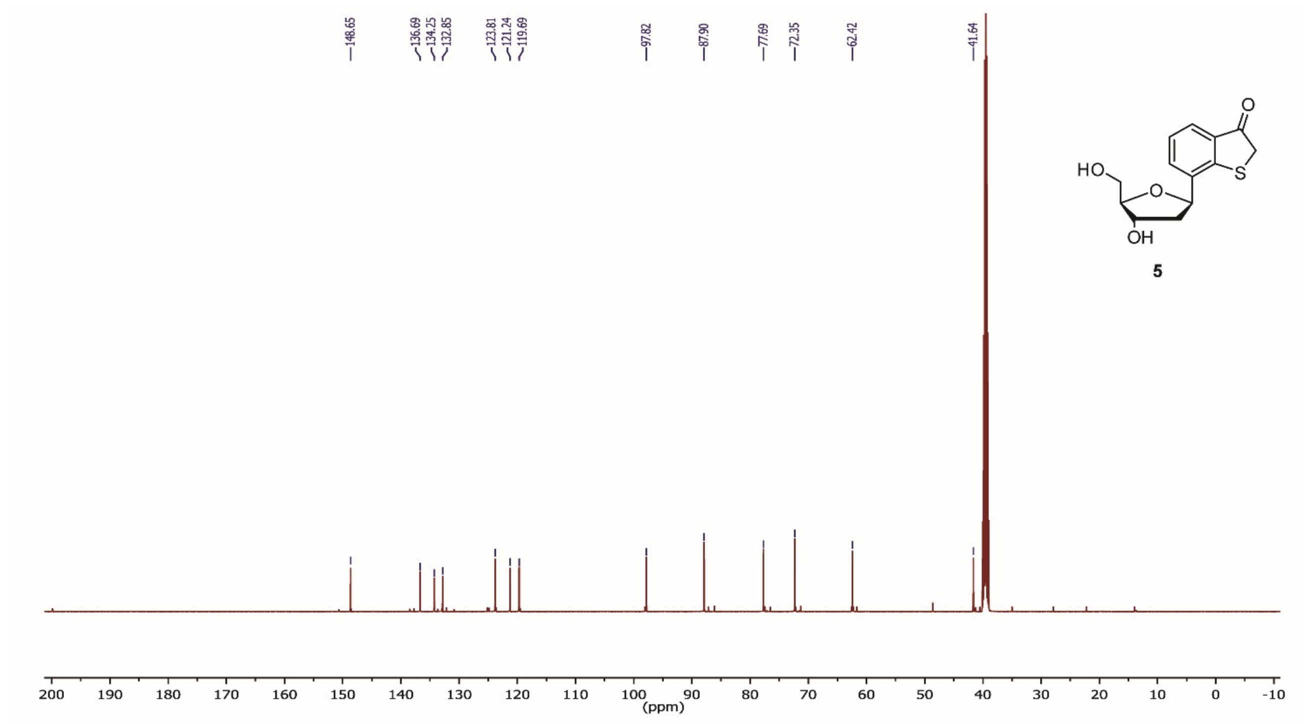

Fig. S25 $^{13}C\{^{1}H\}$-spectrum of compound **5** in DMSO-d_6.

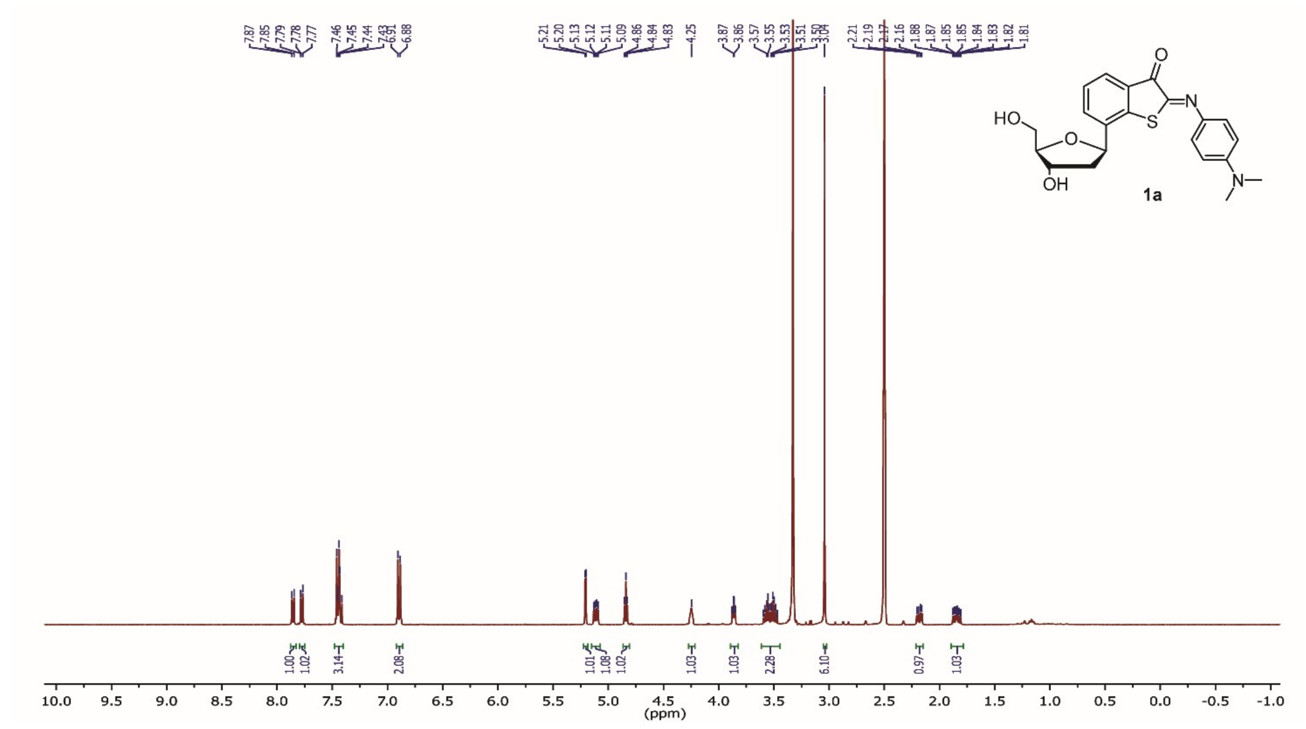

Fig. S26 ^{1}H-spectrum of compound **1a** in DMSO-d_6.

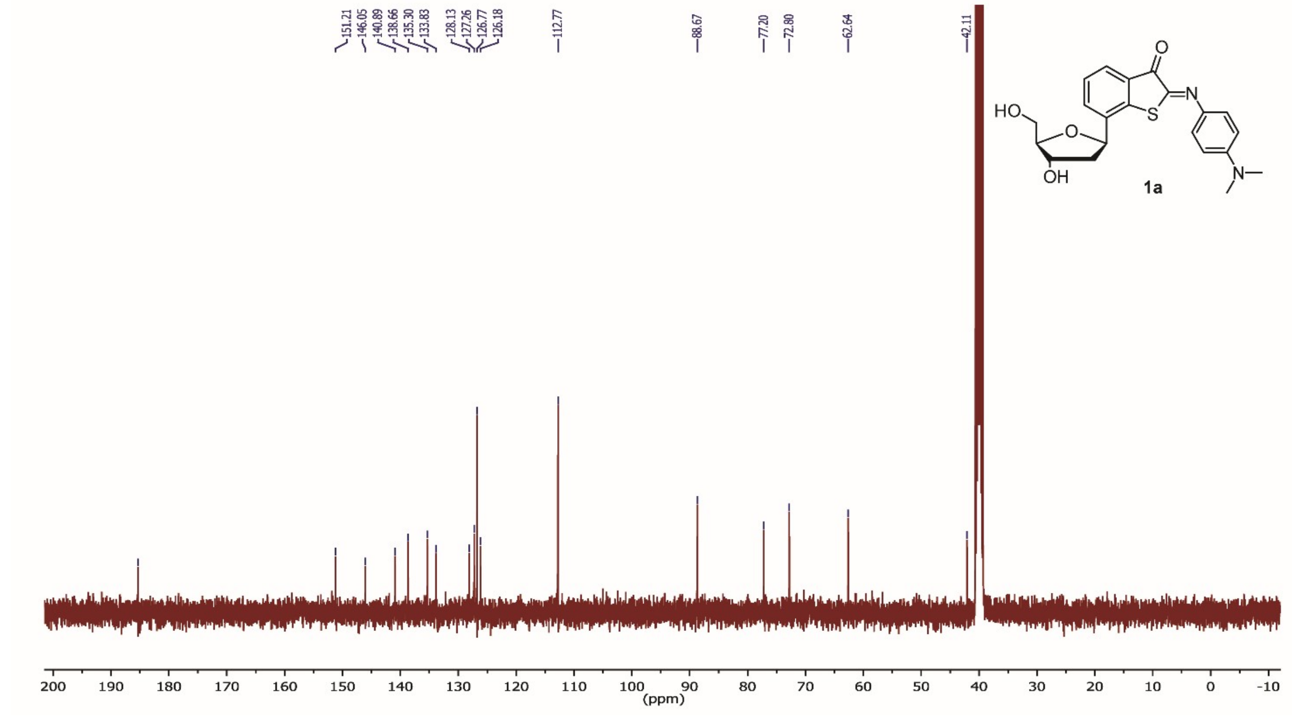

Fig. S27 $^{13}C\{^{1}H\}$-spectrum of compound **1a** in DMSO-d_6.

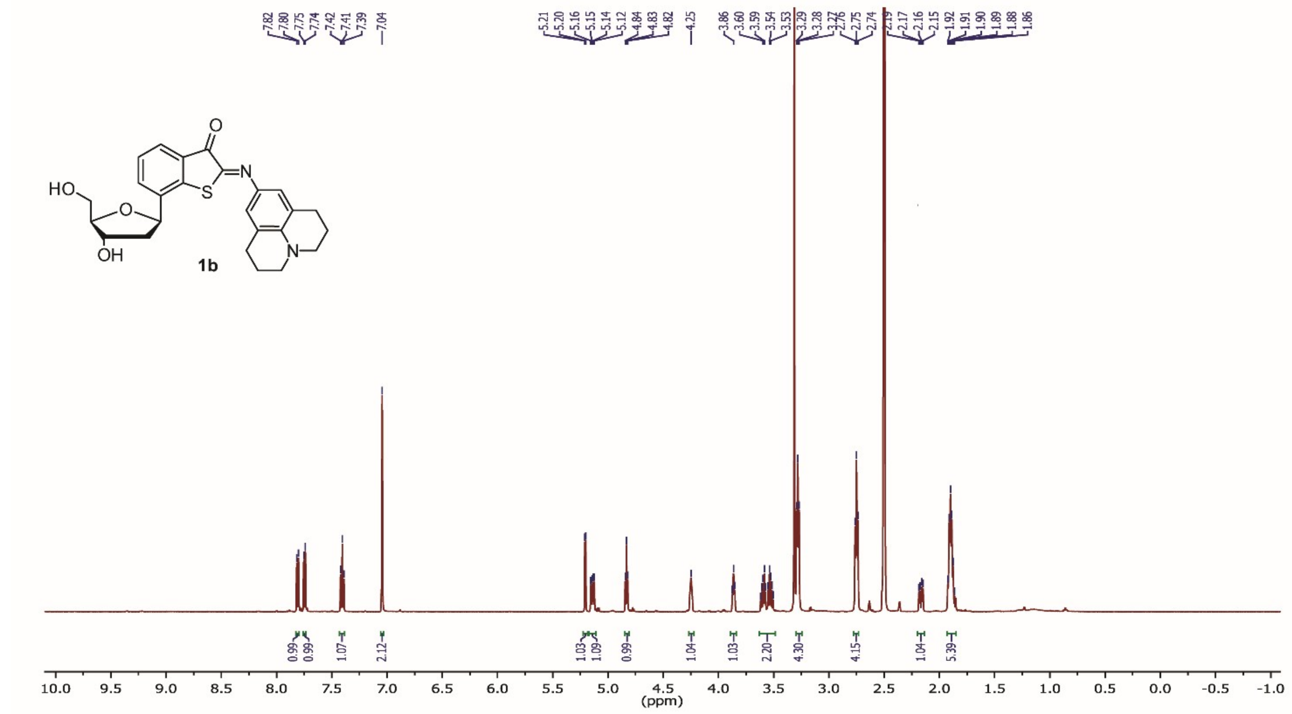

Fig. S28 ^{1}H-spectrum of compound **1b** in DMSO-d_6.

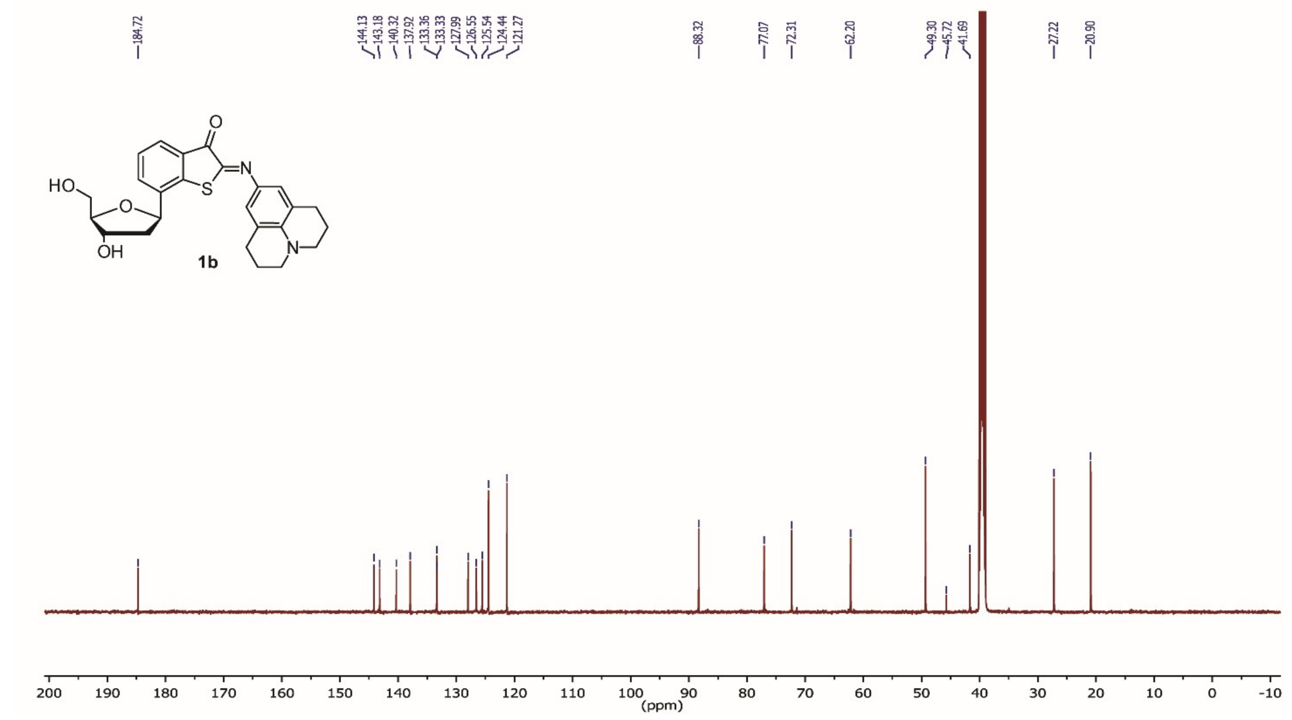

Fig. S29 $^{13}C\{^1H\}$-spectrum of compound **1b** in DMSO-d_6.

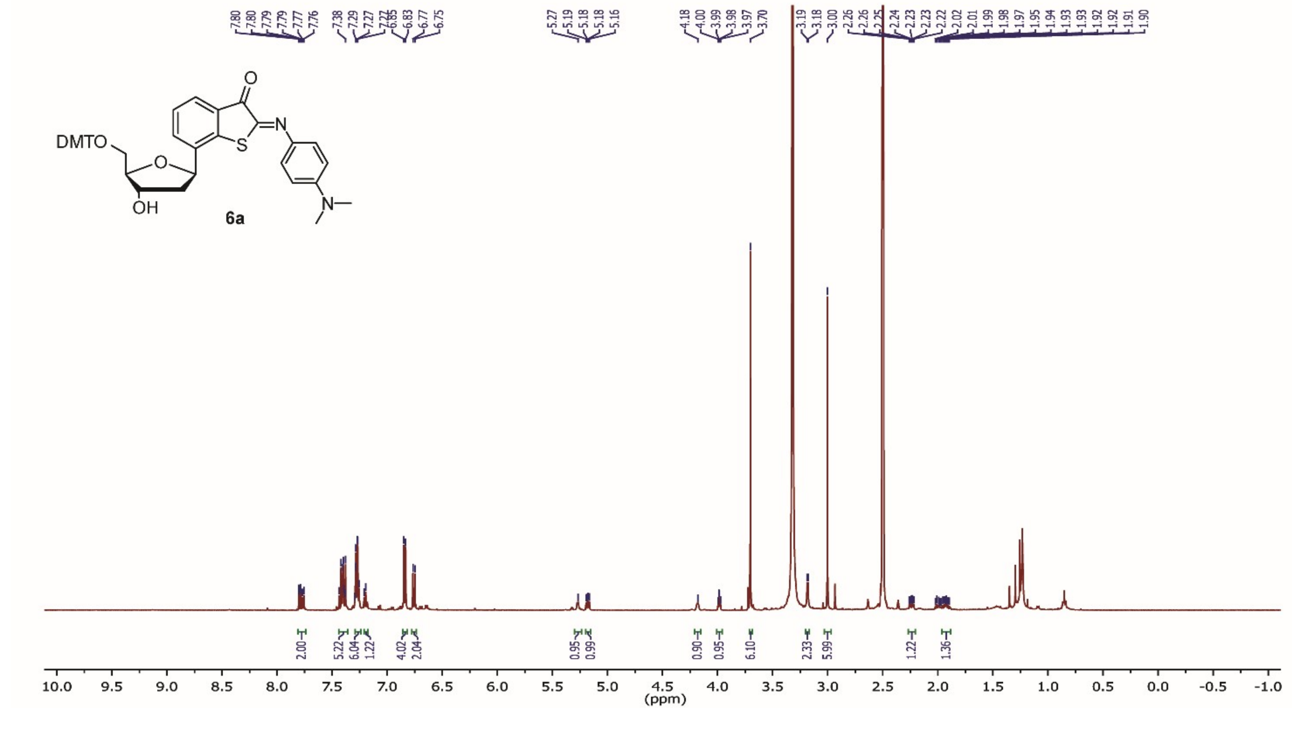

Fig. S30 ^1H-spectrum of compound **6a** in DMSO-d_6.

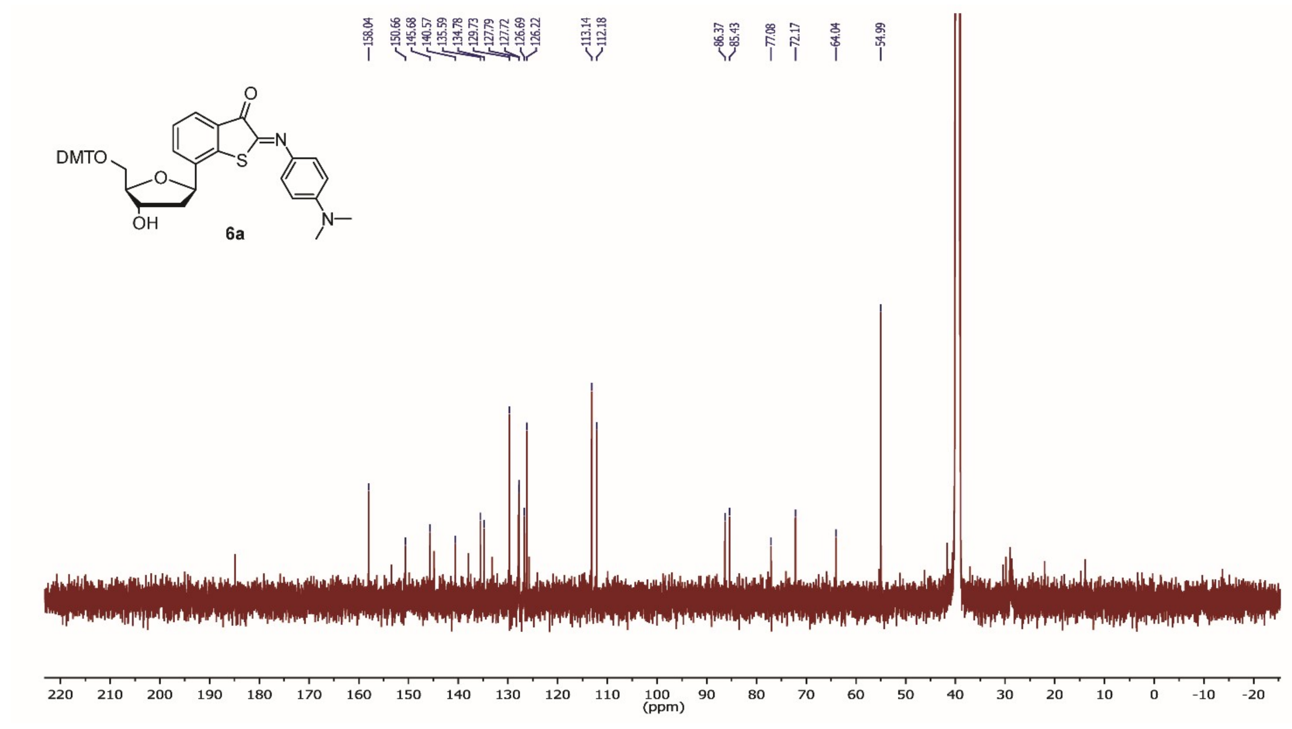

Fig. S31 $^{13}C\{^{1}H\}$-spectrum of compound **6a** in DMSO-d_6.

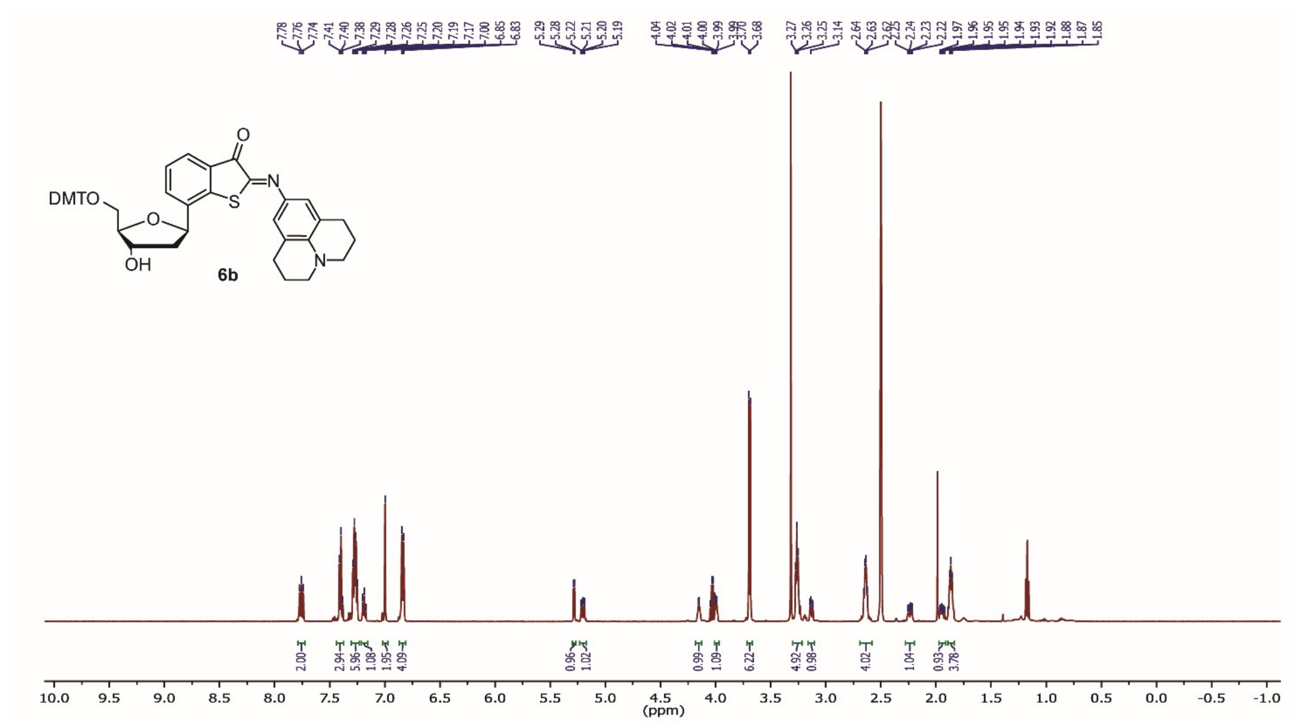

Fig. S32 ^{1}H-spectrum of compound **6b** in DMSO-d_6.

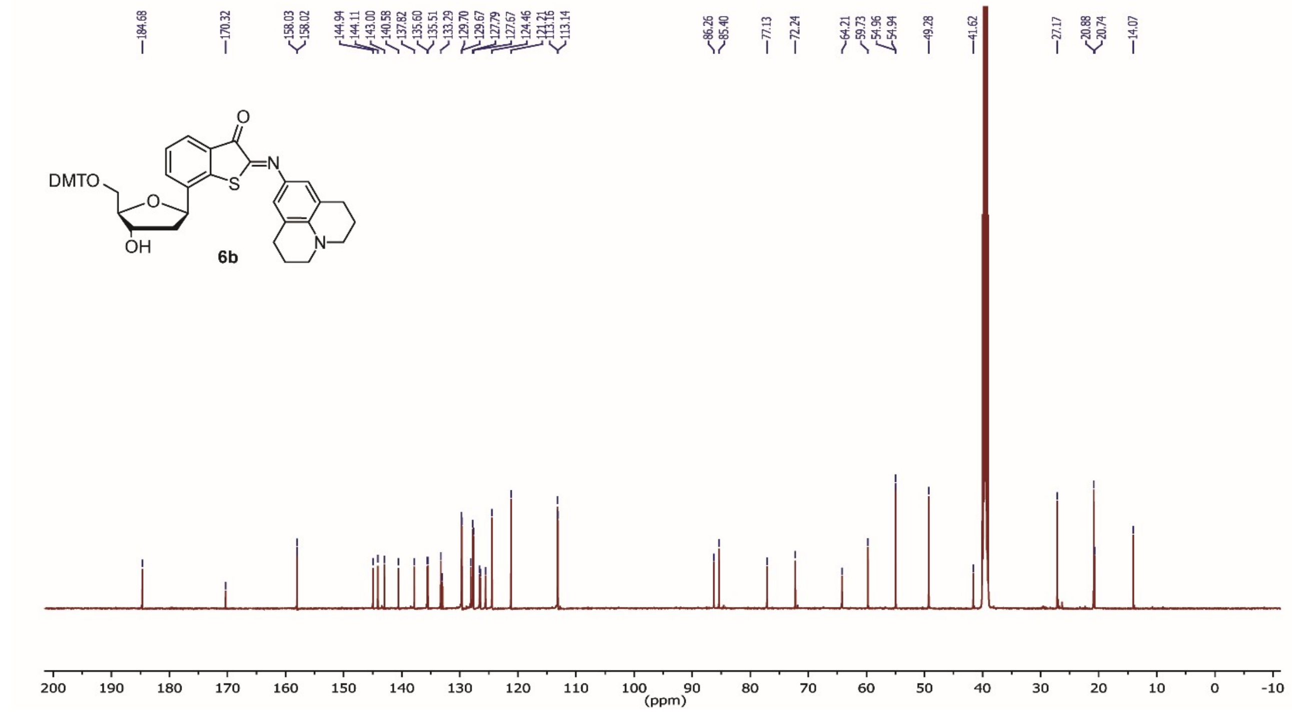

Fig. S33 $^{13}C\{^1H\}$-spectrum of compound 6b in DMSO-d_6.

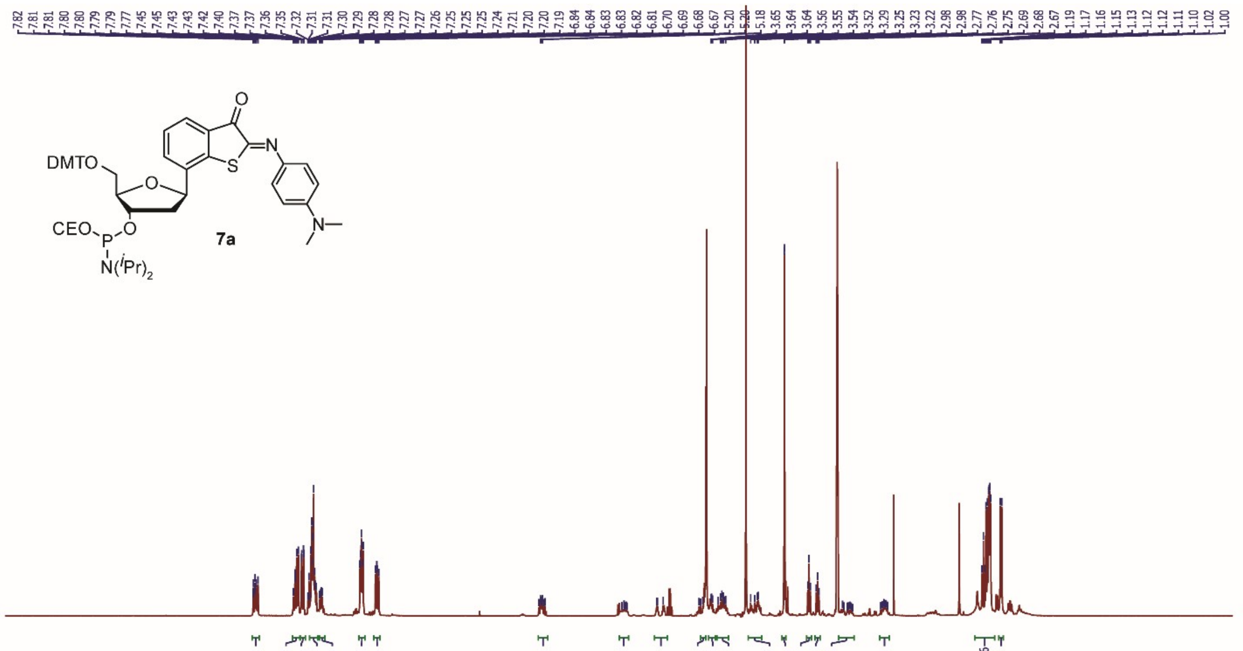

Fig. S34 ^1H-spectrum of compound **7a** in DMSO-d_6.

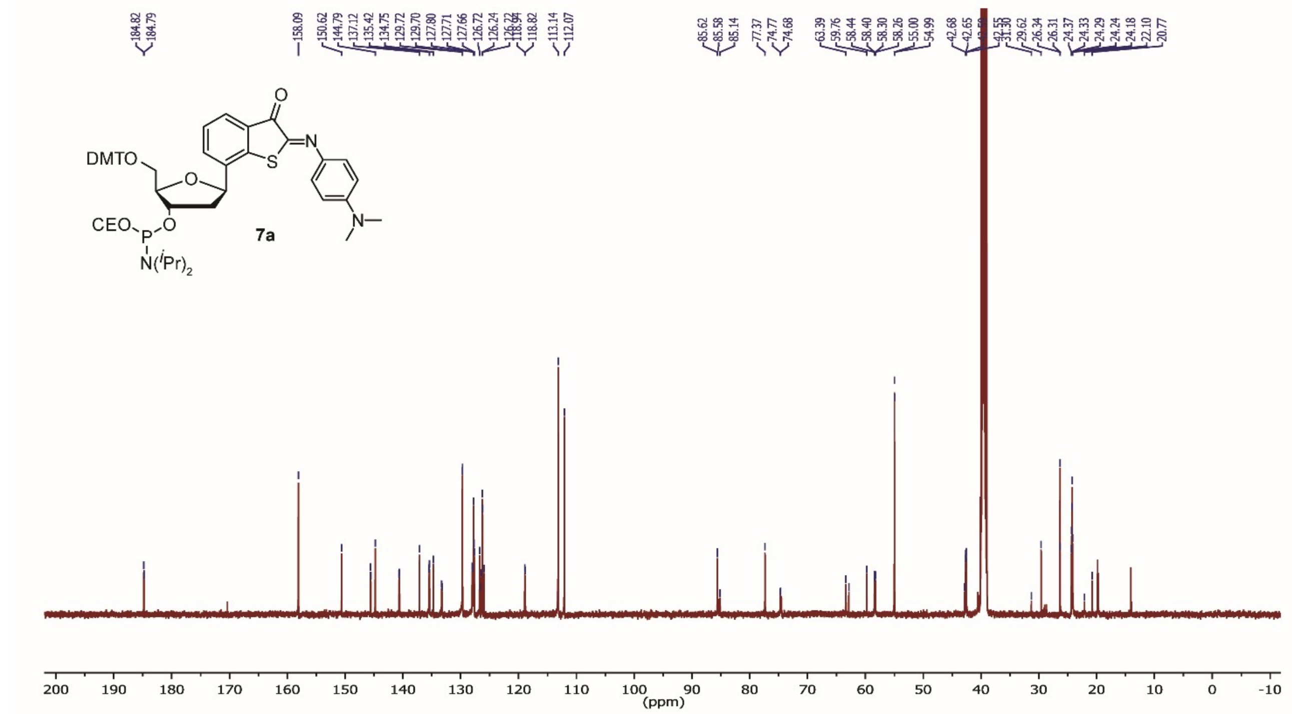

Fig. S35 $^{13}C\{^1H\}$-spectrum of compound **7a** in DMSO-d_6.

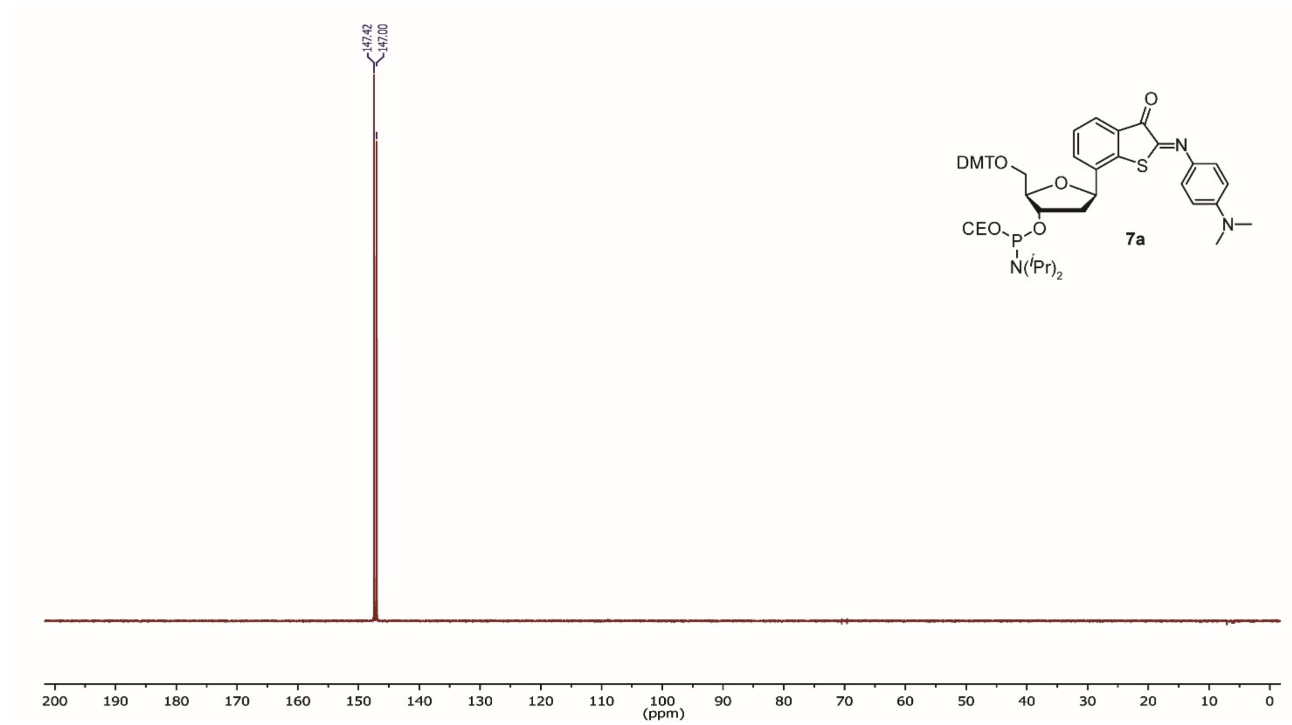

Fig. S36 $^{31}P\{^1H\}$-spectrum of compound **7a** in DMSO-d_6.

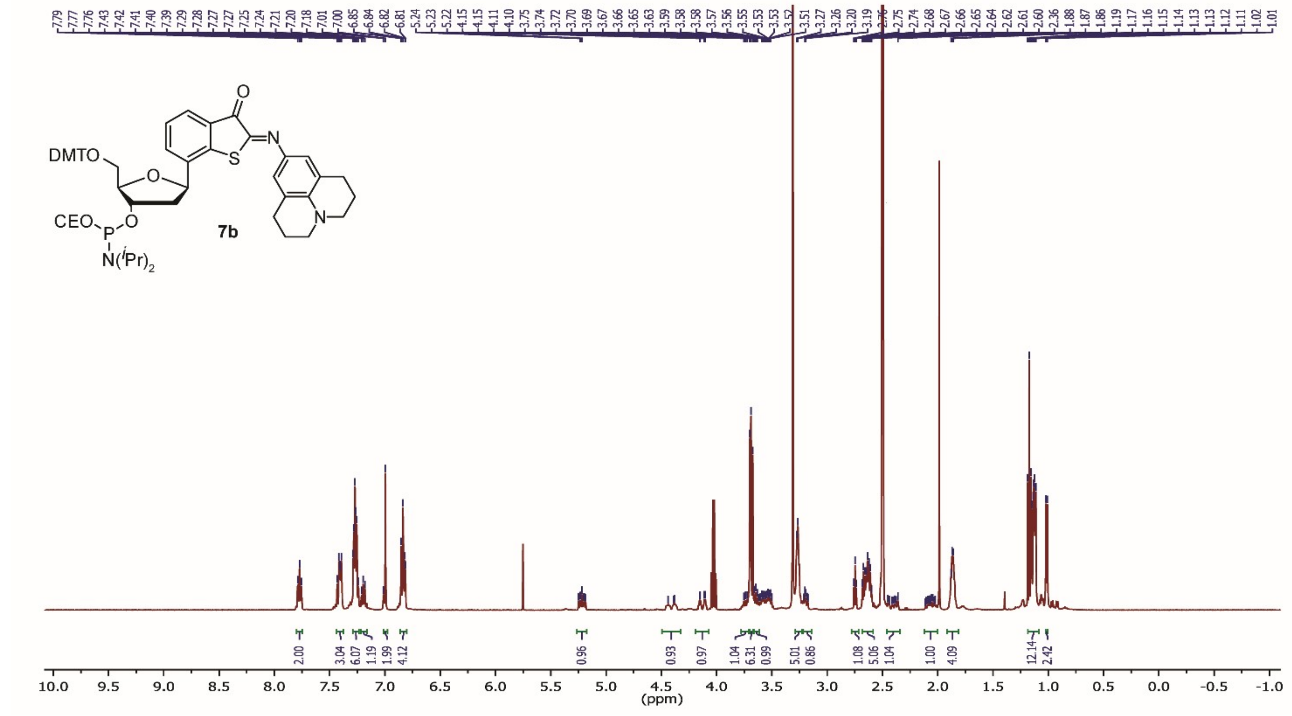

Fig. S37 ^{1}H-spectrum of compound **7b** in DMSO-d_6.

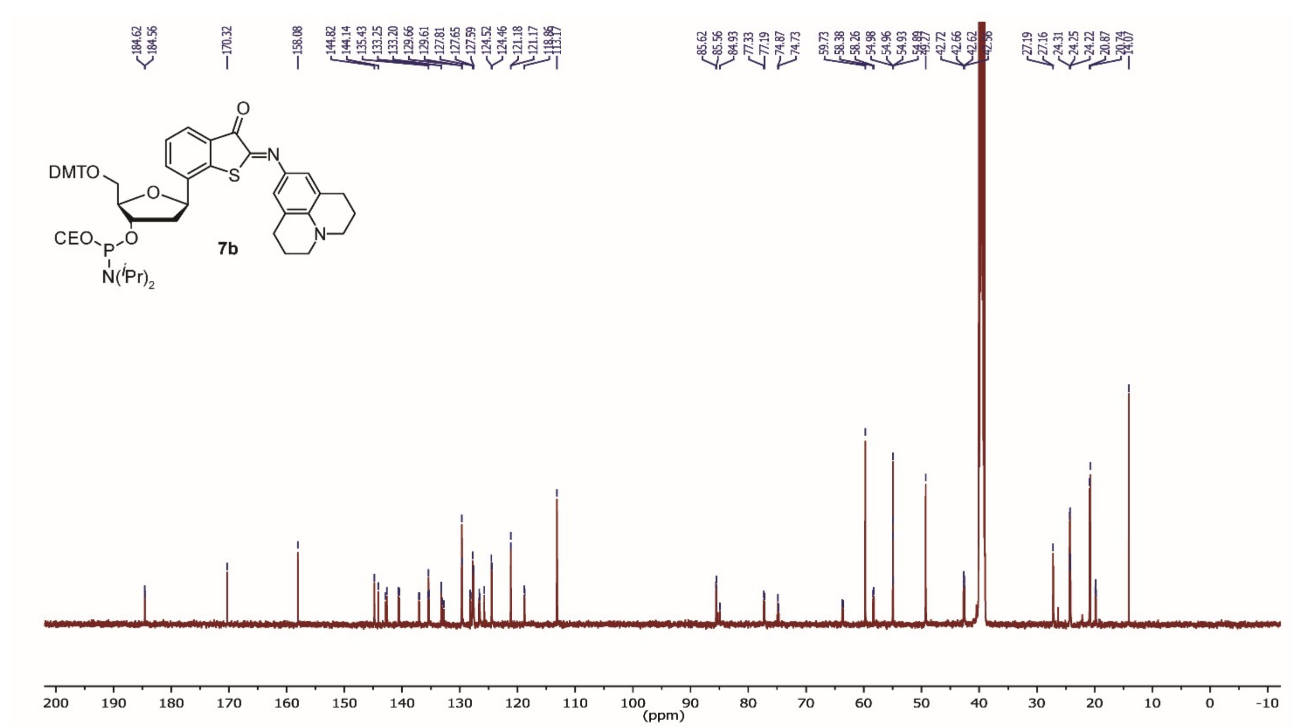

Fig. S38 ^{13}C{^{1}H}-spectrum of compound **7b** in DMSO-d_6.

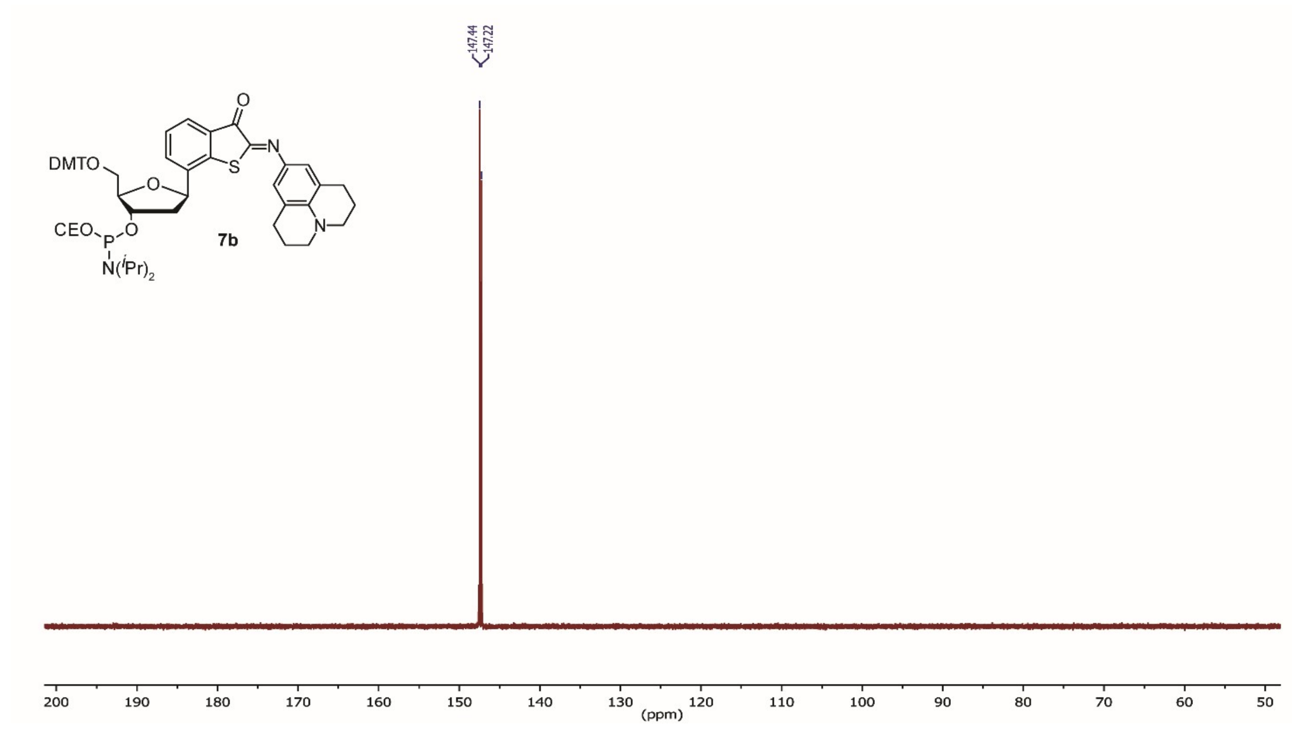

Fig. S39 $^{31}P\{^{1}H\}$-spectrum of compound **7b** in DMSO-d_6.

12. Mass Spectra

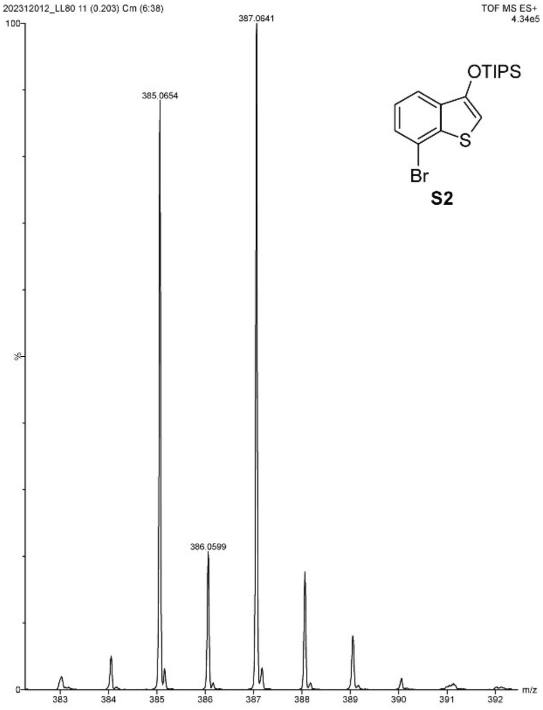

Fig. S40 ESI-HRMS spectra of compound **S2**.

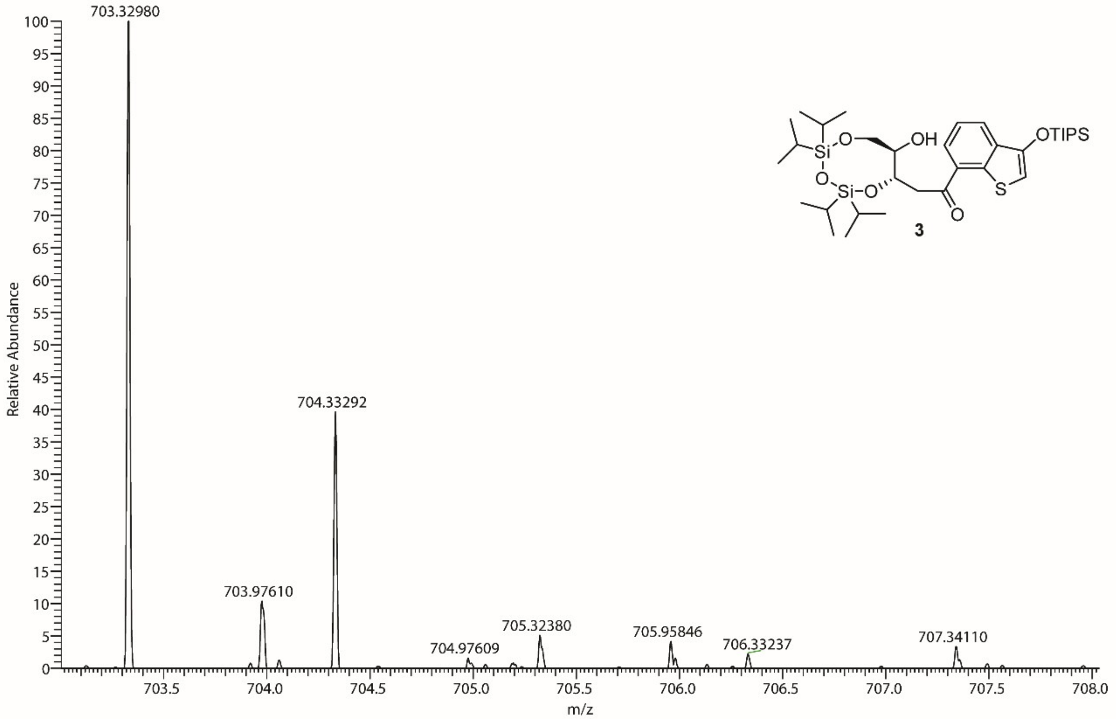

Fig. S41 MALDI-HRMS spectra of compound **3**.

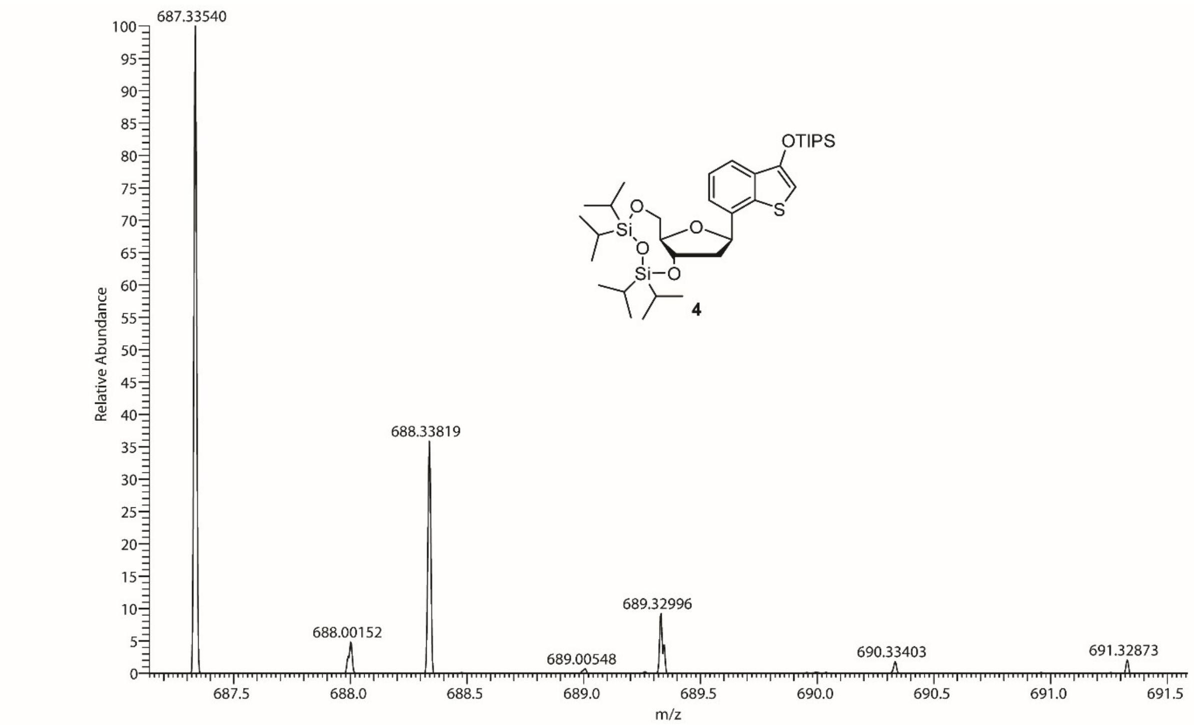

Fig. S42 MALDI-HRMS spectra of compound **4**.

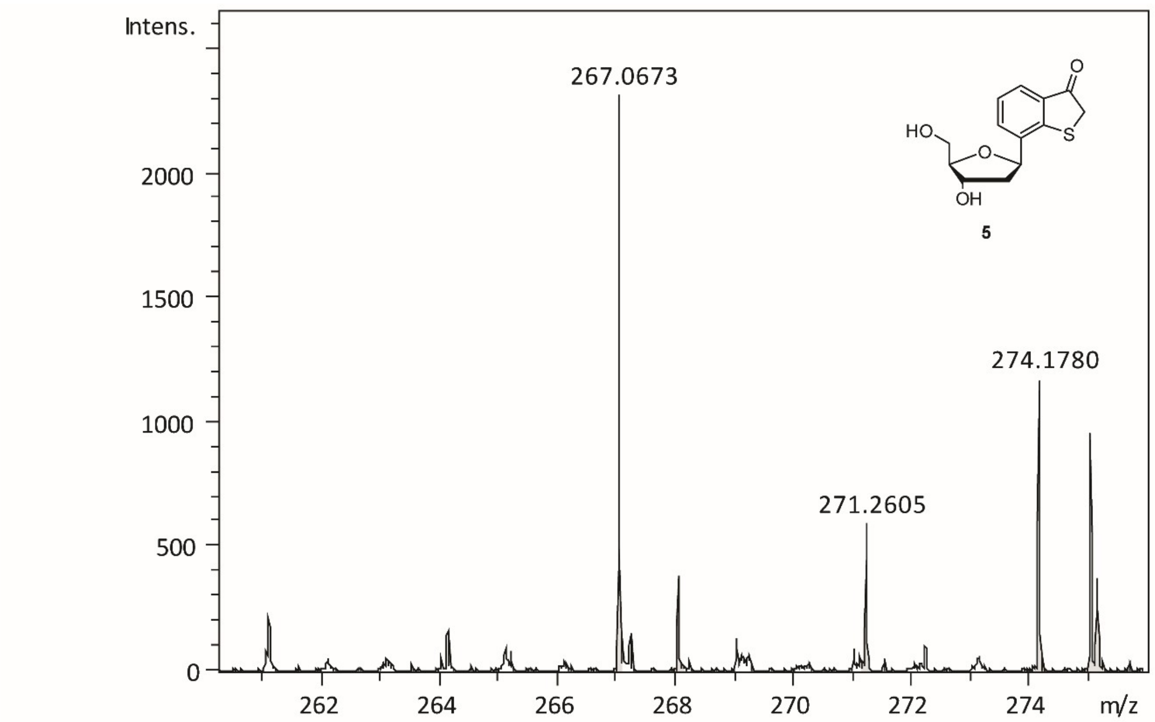

Fig. S43 ESI-HRMS spectra of compound **5**.

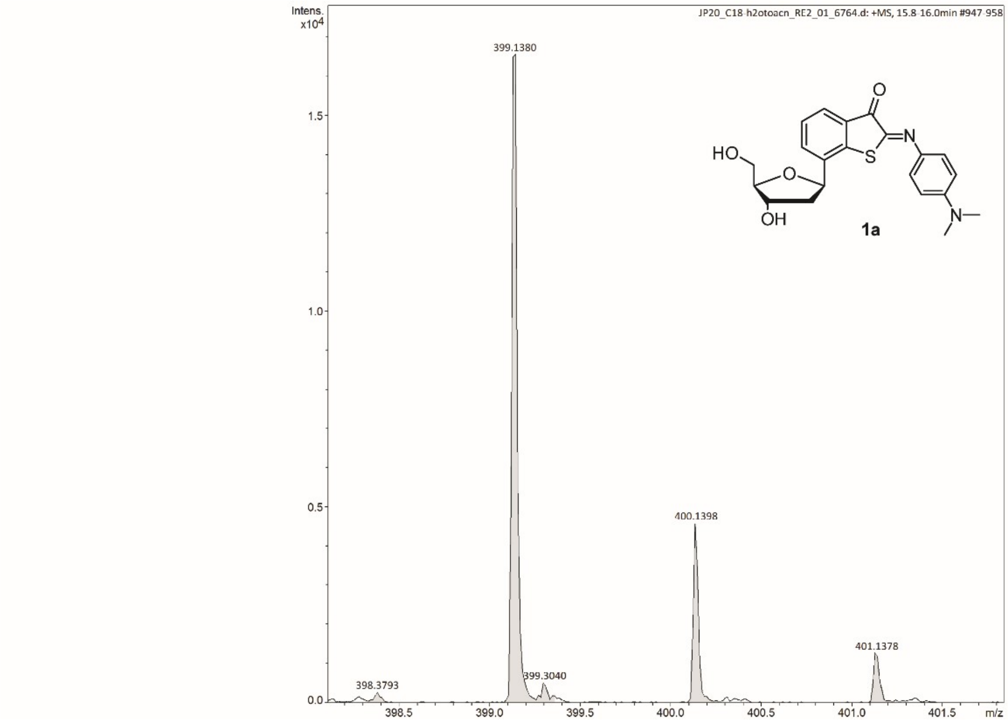

Fig. S44 ESI-HRMS spectra of compound **1a**.

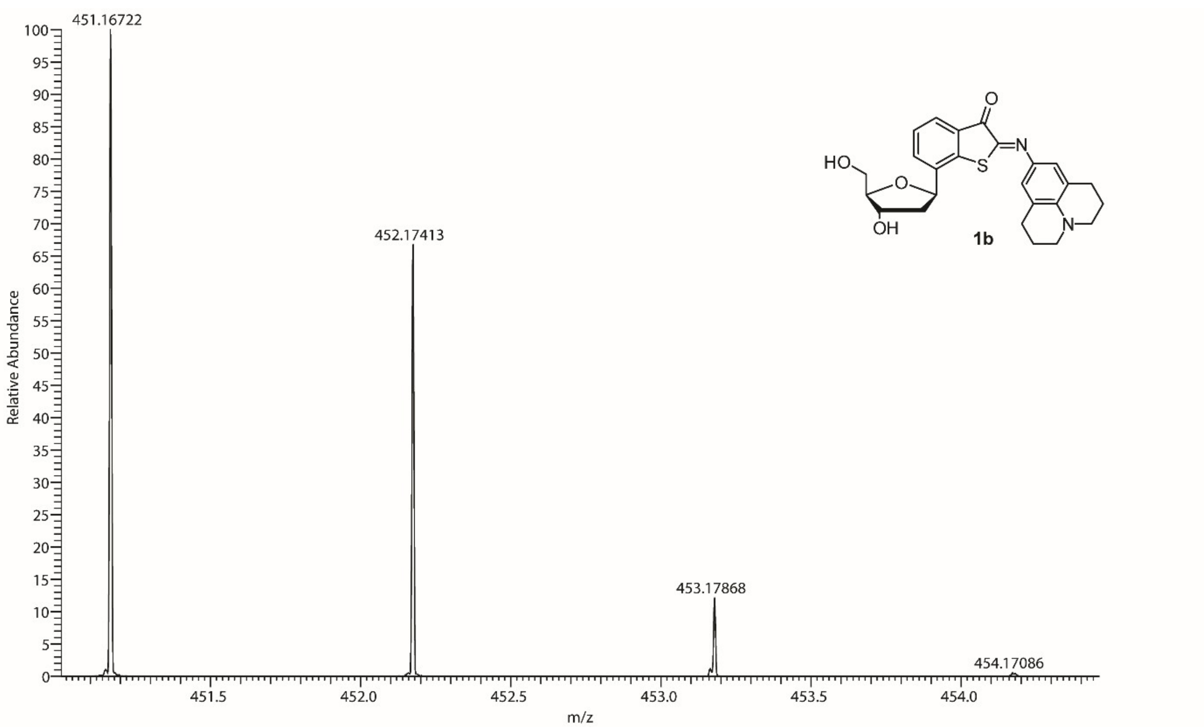

Fig. S45 MALDI-HRMS spectra of compound **1b**.

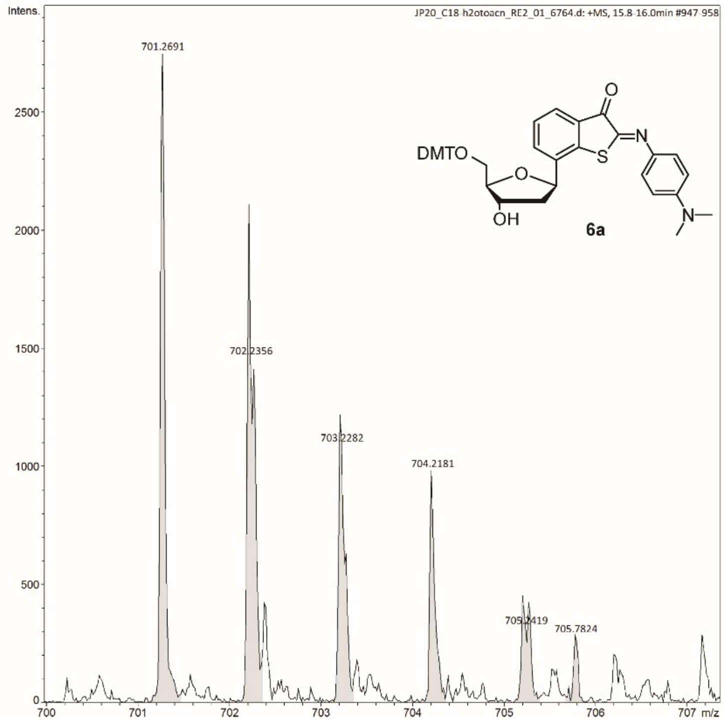

Fig. S46 ESI-HRMS spectra of compound **6a**.

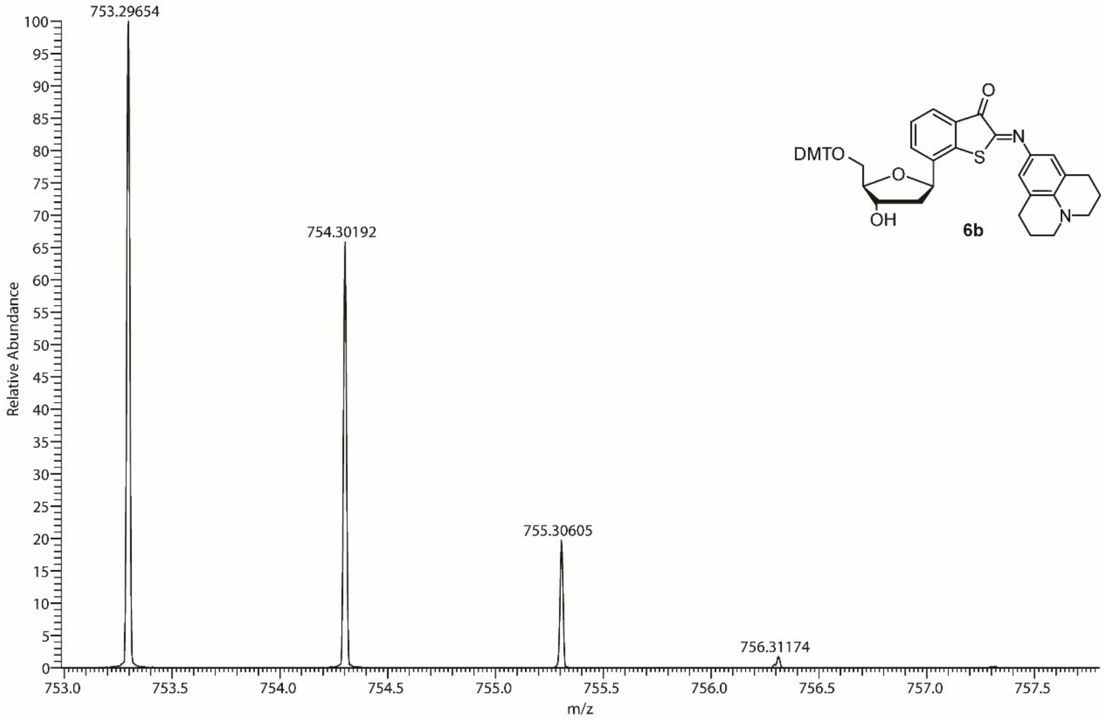

Fig. S47 MALDI-HRMS spectra of compound **6b**.

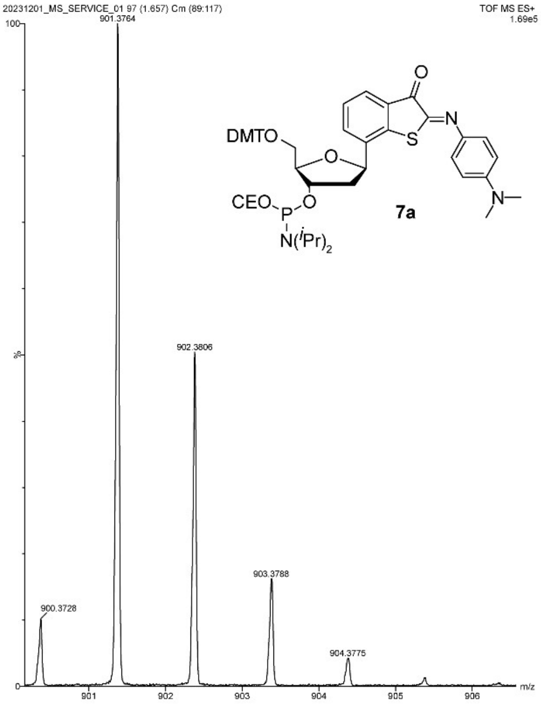

Fig. S48 ESI-HRMS spectra of compound **7a**.

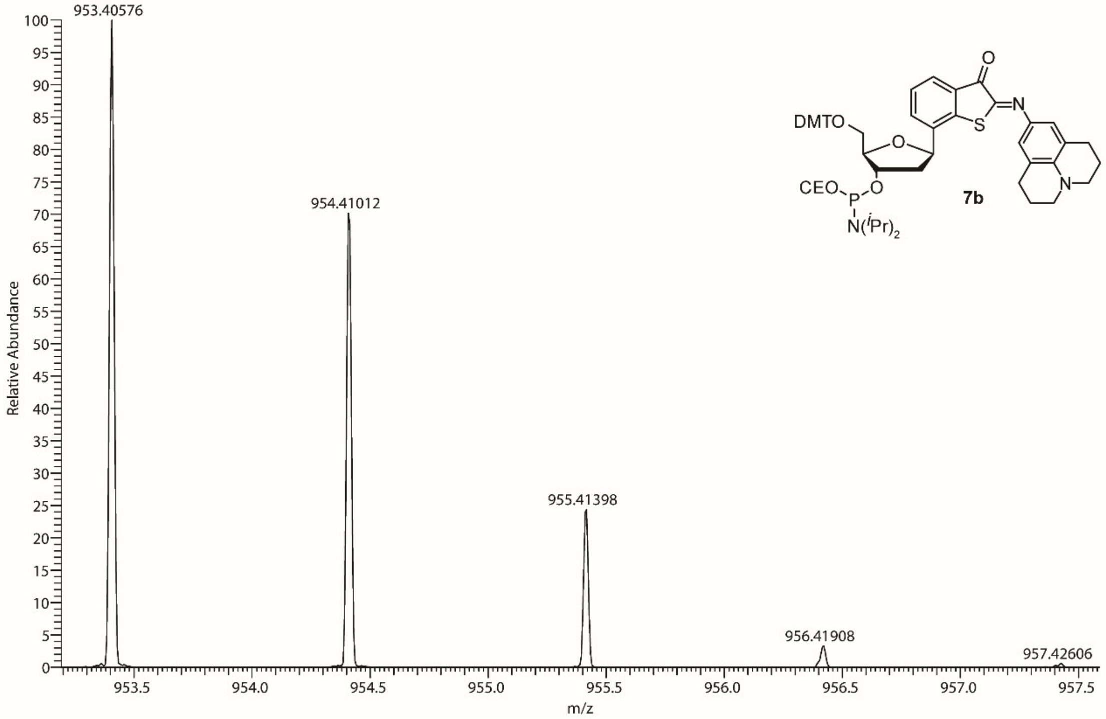

Fig. S49 MALDI-HRMS spectra of compound **7b**.

13. Oligonucleotide Mass Spectra

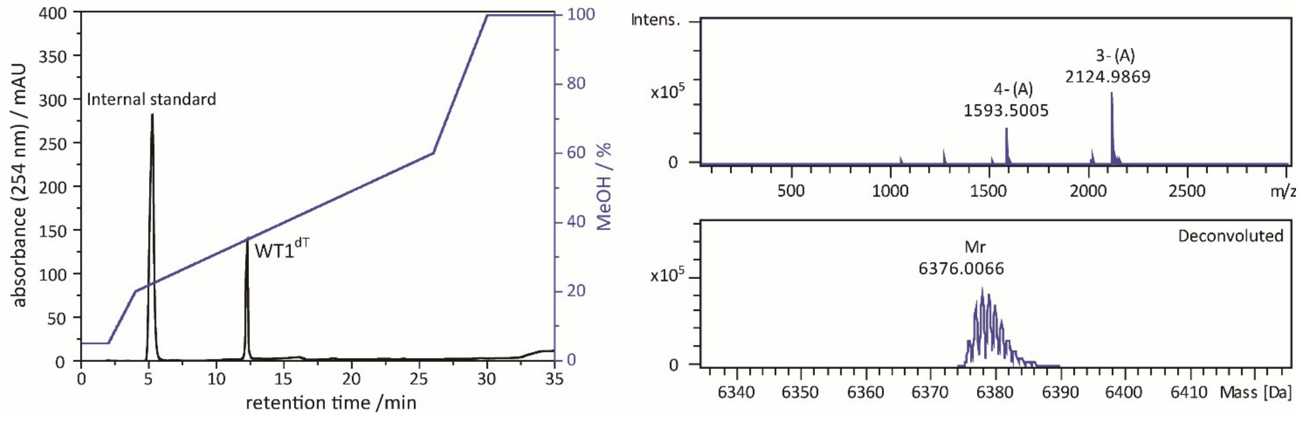

Fig. S50 LC-MS spectra of oligonucleotide **WT1^{dT}**.

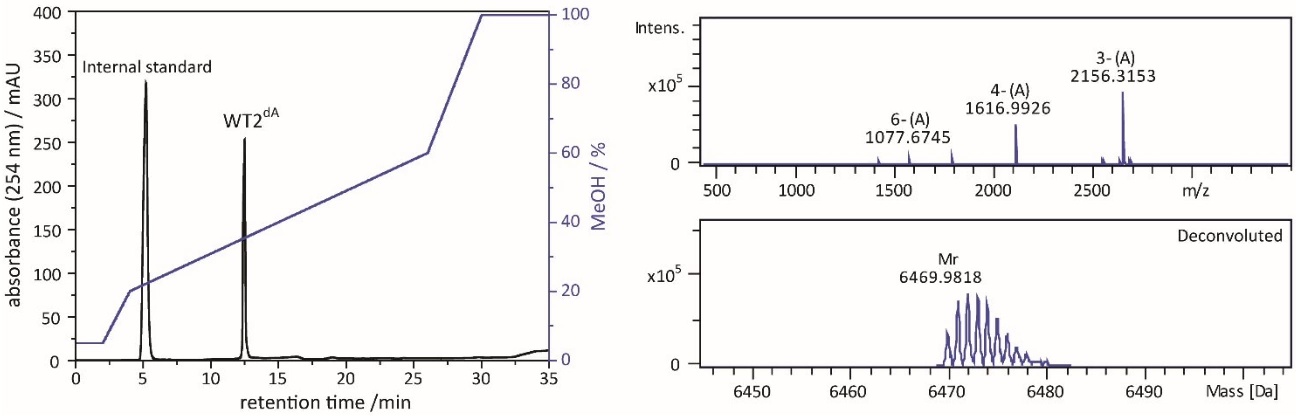

Fig. S51 LC-MS spectra of oligonucleotide **WT2^{dA}**.

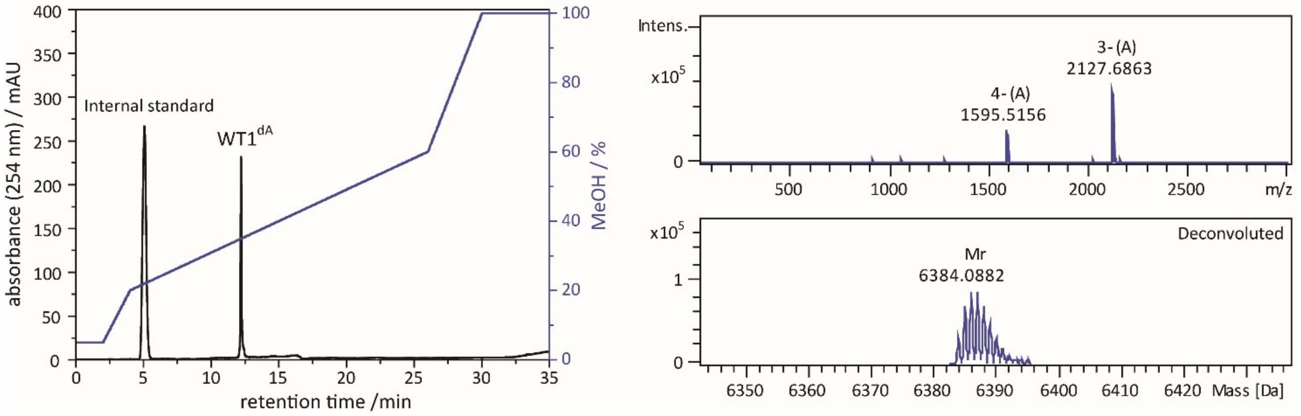

Fig. S52 LC-MS spectra of oligonucleotide **WT1^{dA}**.

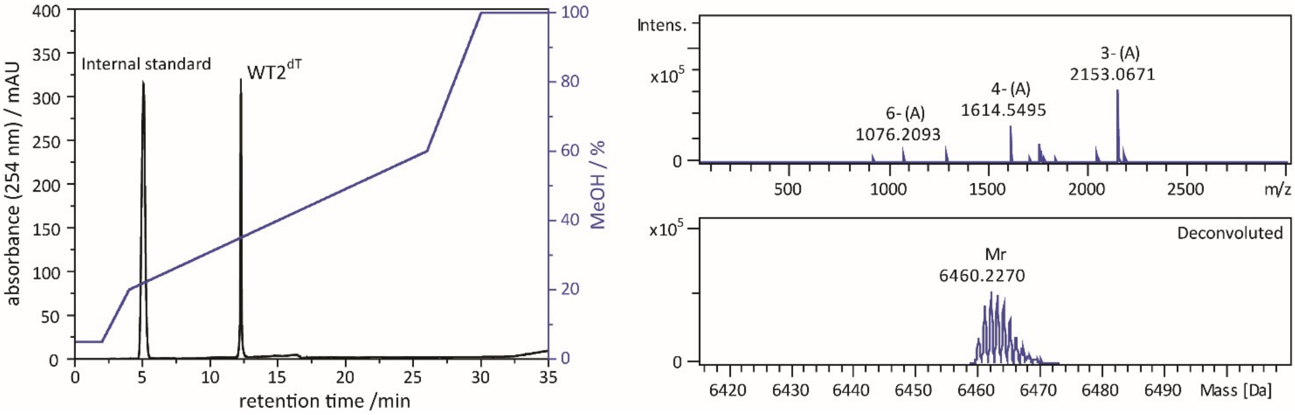

Fig. S53 LC-MS spectra of oligonucleotide **WT2^{dT}**.

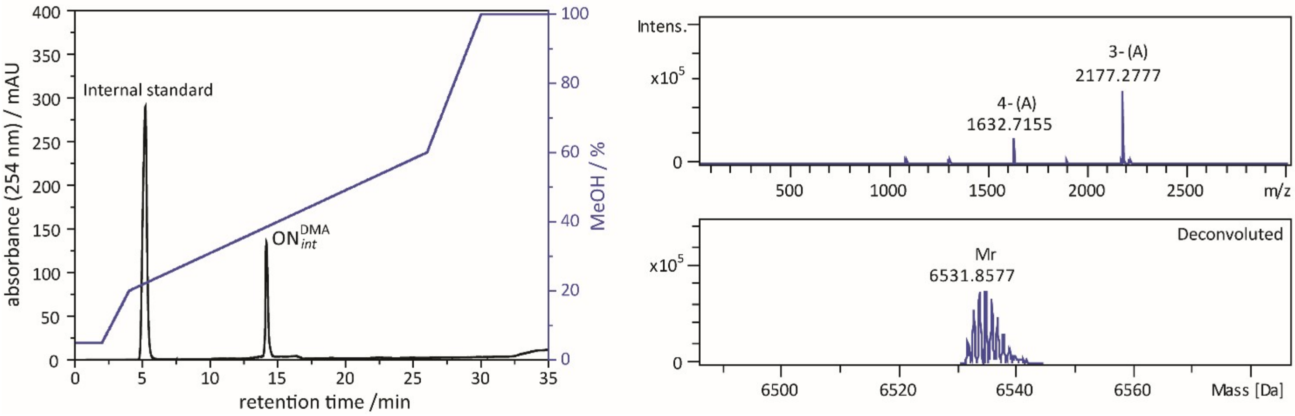

Fig. S54 LC-MS spectra of oligonucleotide $\mathbf{ON}^{\mathbf{DMA}}_{\boldsymbol{int}}$.

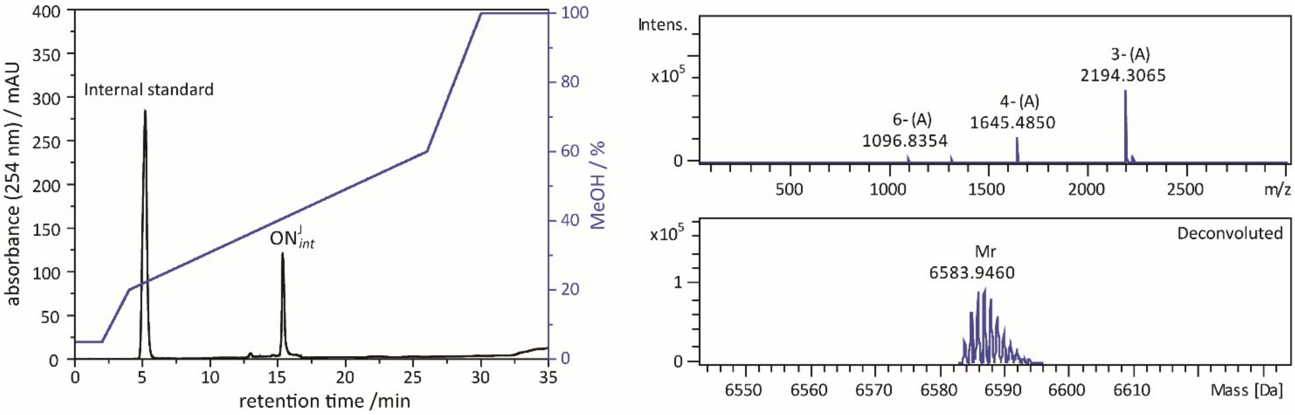

Fig. S55 LC-MS spectra of oligonucleotide $\mathbf{ON}^{\mathbf{J}}_{\boldsymbol{int}}$.

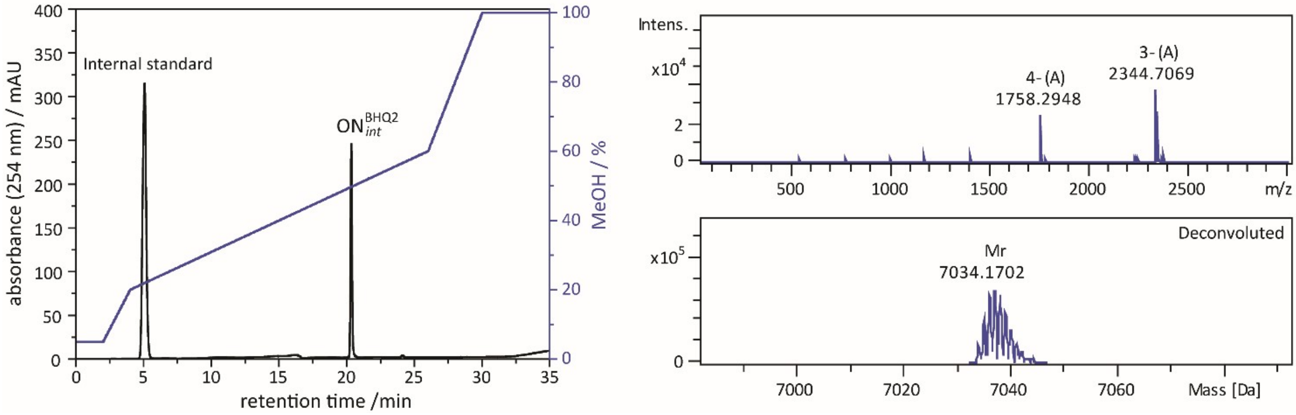

Fig. S56 LC-MS spectra of oligonucleotide $\mathbf{ON}^{\mathbf{BHQ2}}_{\boldsymbol{int}}$.

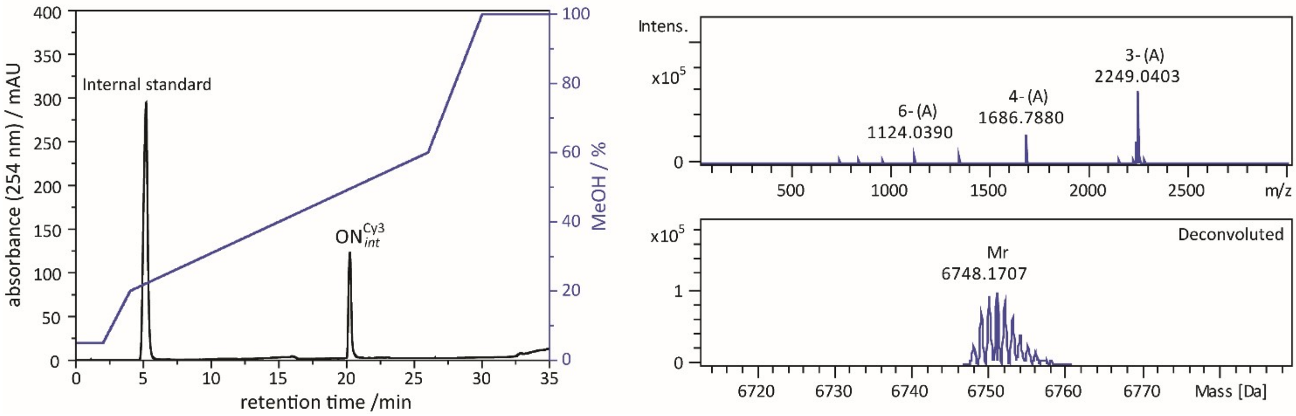

Fig. S57 LC-MS spectra of oligonucleotide $\mathbf{ON}^{\mathbf{Cy3}}_{\boldsymbol{int}}$.

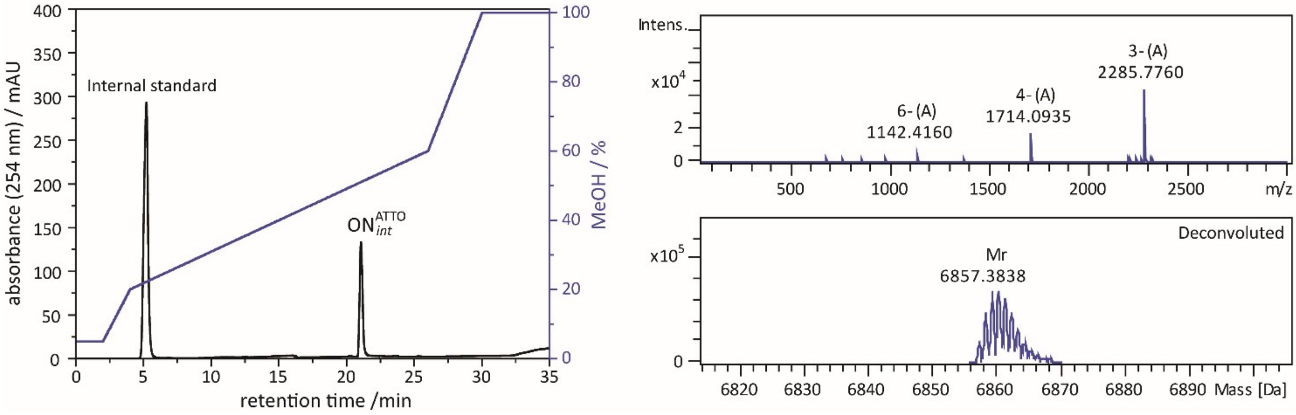

Fig. S58 LC-MS spectra of oligonucleotide $\mathbf{ON}^{\mathbf{ATTO}}_{\boldsymbol{int}}$.

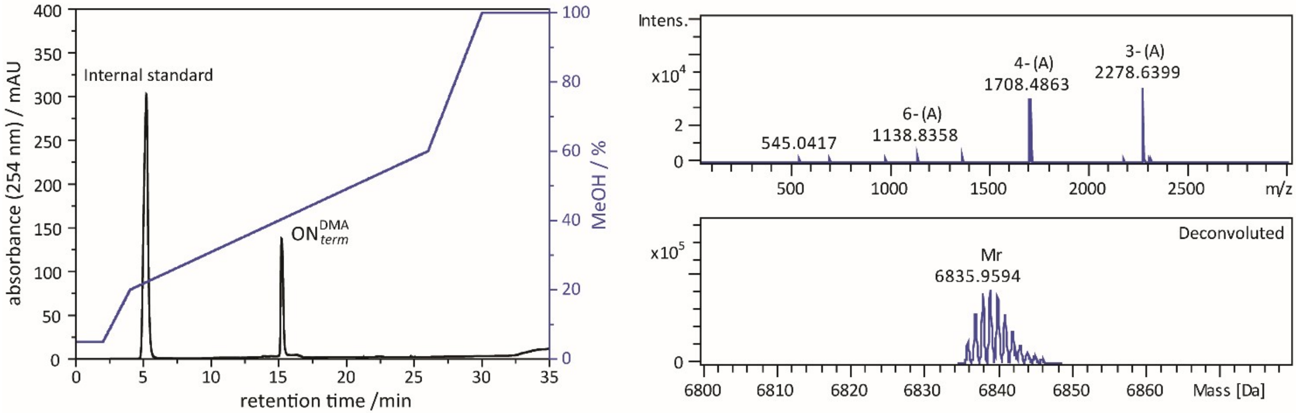

Fig. S59 LC-MS spectra of oligonucleotide $\mathbf{ON}^{\mathbf{DMA}}_{\boldsymbol{term}}$.

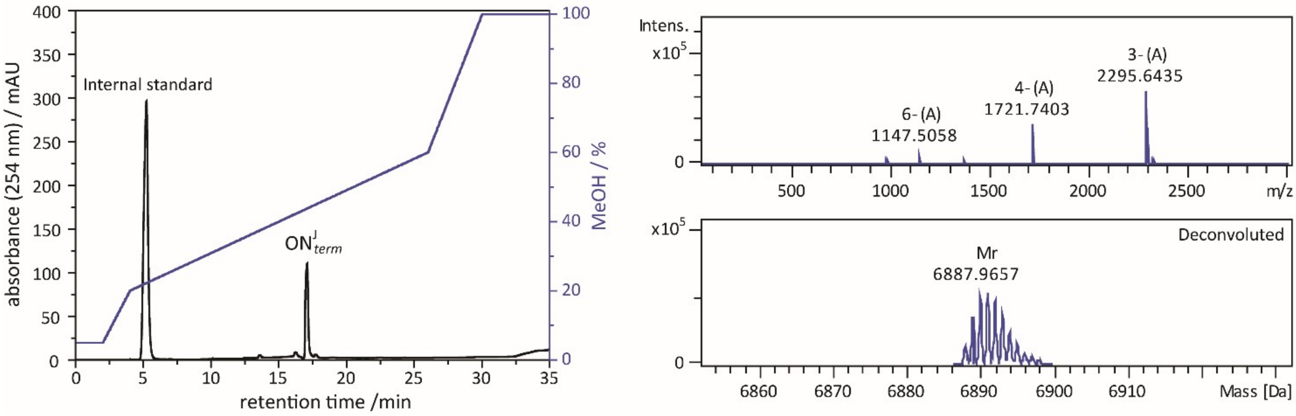

Fig. S60 LC-MS spectra of oligonucleotide $\mathbf{ON}^{\mathbf{J}}_{\boldsymbol{term}}$.

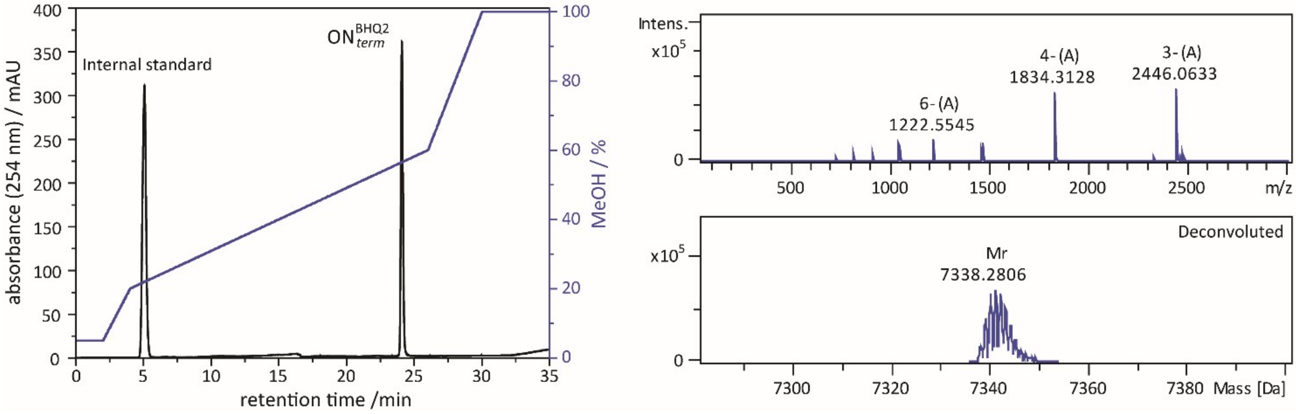

Fig. S61 LC-MS spectra of oligonucleotide $\mathbf{ON}^{\mathbf{BHQ2}}_{\boldsymbol{term}}$.

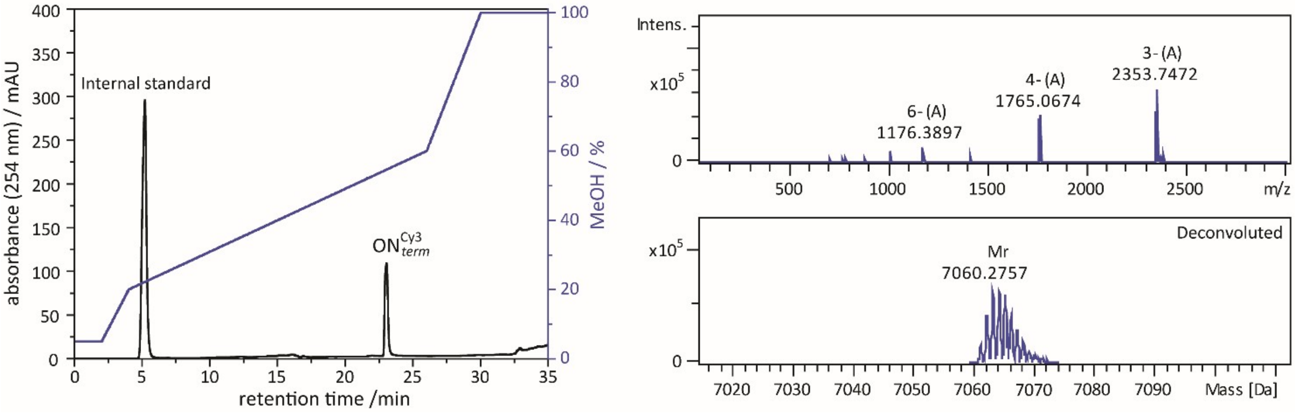

Fig. S62 LC-MS spectra of oligonucleotide ON^{Cy3}_{term}.

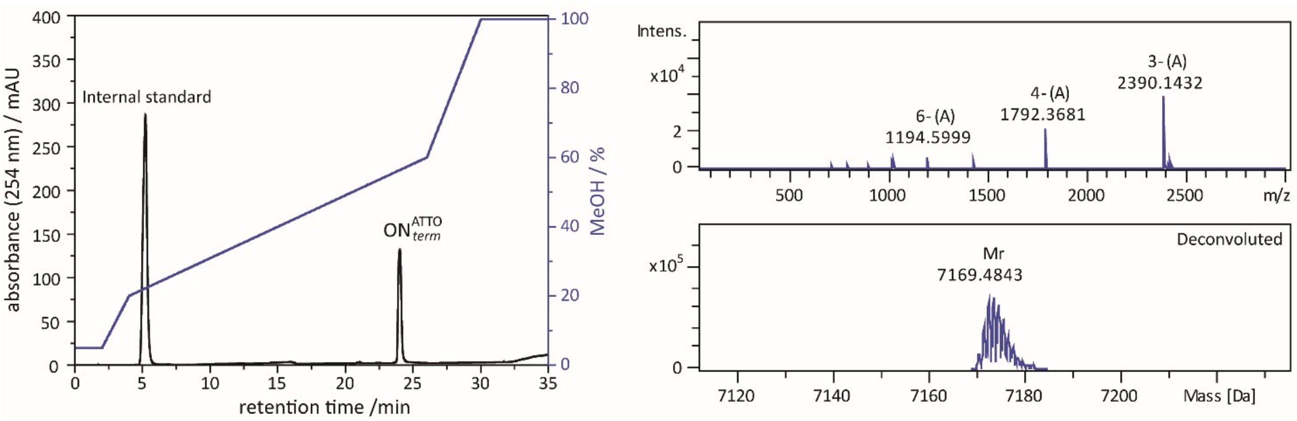

Fig. S63 LC-MS spectra of oligonucleotide ON^{ATTO}_{term}.

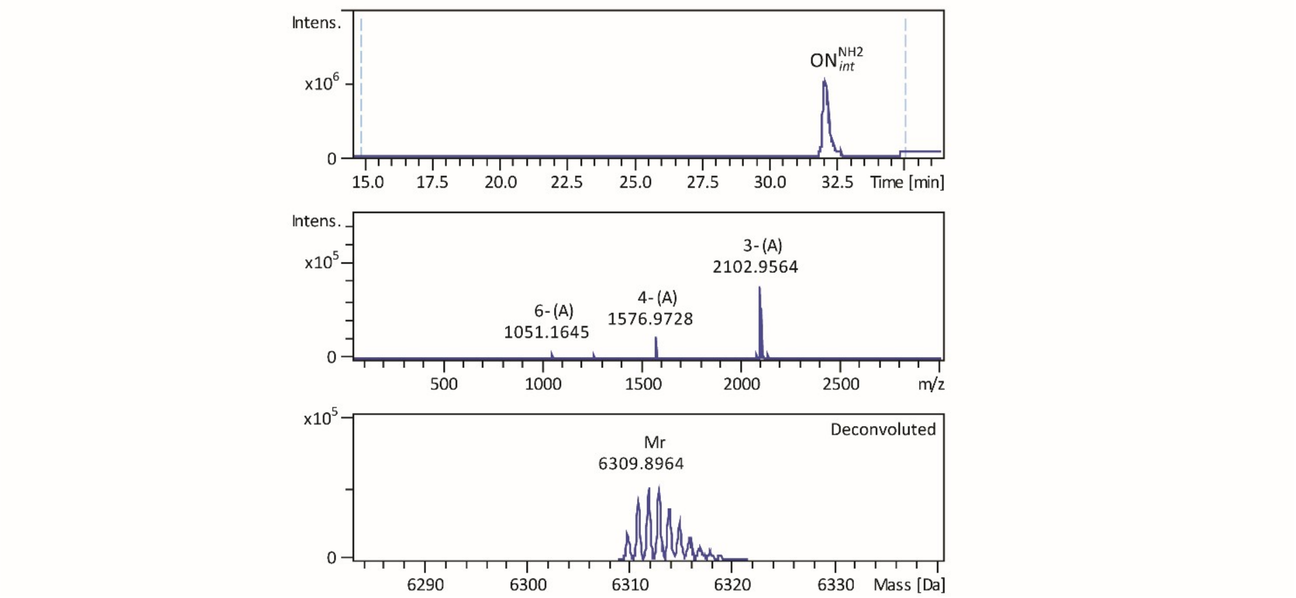

Fig. S64 LC-MS spectra of oligonucleotide ON^{NH2}_{int}.

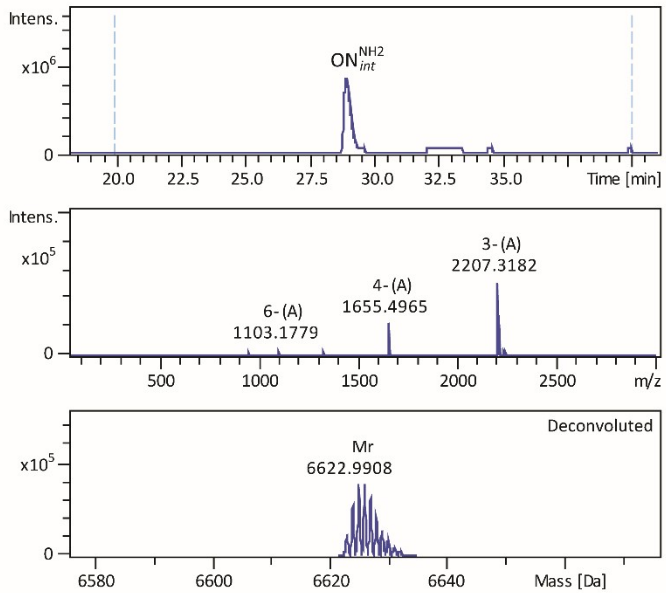

Fig. S65 LC-MS spectra of oligonucleotide $\mathbf{ON}^{\mathbf{NH2}}_{\boldsymbol{term}}$.

14. List of Abbreviations

Abs.	absorption
(Ac)	(acetyl)
ACN	acetonitrile
AcOH	acetic acid
ATTO Rho11	ATTO Rhodamine 11
BTP	benzothiophenone
CPG	controlled pore glass
Cy3	Cyanine 3
dA	desoxyadenosine
dC	deoxycytidine
DEPC	diethyl pyrocarbonate
dG	deoxyguanosine
DMA-	dimethylamino-
DMT (Cl)	dimethoxytrityl (chloride)
dT	deoxythymidine
ESA	excited state absorption
Em.	emission
Fmoc	fluorenylmethyloxycarbonyl
GSB	ground state bleach
HFIP	hexafluoroisopropanol
(*i*Pr-Pac)	isopropyl phenoxyacetyl
ITI	iminothioindoxyl
J-	julolidine
LDA	lifetime distribution analysis
LDM	lifetime density map
mod.	modification
NOPA	noncollinear optical parametric amplifier
ON	oligonucleotide
PA	product absorption
(Pac)	phenoxyacetyl
PBS	phosphate buffered saline
SE	stimulated emission
TBAF	tetrabutylammonium fluoride
TCA	trichloroacetic acid
TIPS (Cl)	triisopropylsilyl (chloride)

15. References

1 T. Yamaguchi, T. Seki, T. Tamaki and K. Ichimura, *Bull. Chem. Soc. Jpn.*, 1992, **65**, 649–656.
2 C. Feid, L. Luma, T. Fischer, J. G. Löffler, N. Grebenovsky, J. Wachtveitl, A. Heckel and J. Bredenbeck, *Angew. Chem. Int. Ed.*, 2023, e202317047.
3 T. Lambert, FPbase Fluorescence Spectra Viewer, https://www.fpbase.org/spectra/, (accessed March 8, 2024).
4 C. Slavov, N. Bellakbil, J. Wahl, K. Mayer, K. Rück-Braun, I. Burghardt, J. Wachtveitl and M. Braun, *Phys. Chem. Chem. Phys.*, 2015, **17**, 14045–14053.
5 T. Wilhelm, J. Piel and E. Riedle, *Opt. Lett.*, 1997, **22**, 1494.
6 E. Riedle, M. Beutter, S. Lochbrunner, J. Piel, S. Schenkl, S. Spörlein and W. Zinth, *Appl. Phys. B*, 2000, **71**, 457–465.
7 C. Slavov, H. Hartmann and J. Wachtveitl, *Anal. Chem.*, 2015, **87**, 2328–2336.
8 S. A. Kovalenko, A. L. Dobryakov, J. Ruthmann and N. P. Ernsting, *Phys. Rev. A*, 1999, **59**, 2369–2384.
9 R. Croce, M. G. Müller, R. Bassi and A. R. Holzwarth, *Biophys. J.*, 2001, **80**, 901–915.
10 H. J. C. Berendsen, D. Van Der Spoel and R. Van Drunen, Computer, *Phys. Commun.*, 1995, **91**, 43–56.
11 R. A. de Groot and J. Nadrchal, Eds., *Physics computing '92: proceedings of the 4th international conference*, Prague, Czechoslovakia, August 24-28, 1992, World Scientific, Singapore, 1993.
12 D. A. Case, H. M. Aktulga, K. Belfon, D. S. Cerutti, G. A. Cisneros, V. W. D. Cruzeiro, N. Forouzesh, T. J. Giese, A. W. Götz, H. Gohlke, S. Izadi, K. Kasavajhala, M. C. Kaymak, E. King, T. Kurtzman, T.-S. Lee, P. Li, J. Liu, T. Luchko, R. Luo, M. Manathunga, M. R. Machado, H. M. Nguyen, K. A. O'Hearn, A. V. Onufriev, F. Pan, S. Pantano, R. Qi, A. Rahnamoun, A. Risheh, S. Schott-Verdugo, A. Shajan, J. Swails, J. Wang, H. Wei, X. Wu, Y. Wu, S. Zhang, S. Zhao, Q. Zhu, T. E. Cheatham, D. R. Roe, A. Roitberg, C. Simmerling, D. M. York, M. C. Nagan and K. M. Merz, *J. Chem. Inf. Model.*, 2023, **63**, 6183–6191.
13 I. Ivani, P. D. Dans, A. Noy, A. Pérez, I. Faustino, A. Hospital, J. Walther, P. Andrio, R. Goñi, A. Balaceanu, G. Portella, F. Battistini, J. L. Gelpí, C. González, M. Vendruscolo, C. A. Laughton, S. A. Harris, D. A. Case and M. Orozco, *Nat. Methods*, 2016, **13**, 55–58.
14 V. Lindahl, A. Villa and B. Hess, *PLoS Comput. Biol.*, 2017, **13**, e1005463.
15 J. Wang, R. M. Wolf, J. W. Caldwell, P. A. Kollman and D. A. Case, *J. Comput. Chem.*, 2004, **25**, 1157–1174.
